# Reproducibility and relative validity of a semi-quantitative food and beverage frequency questionnaire for Spanish children aged 3 to 11 years: the COME-Kids F&B-FQ

**DOI:** 10.1007/s00431-023-05220-9

**Published:** 2023-10-06

**Authors:** Nancy Babio, Sara de Las Heras-Delgado, Pilar De Miguel-Etayo, Belén Pastor-Villaescusa, Rosaura Leis, Tany E. Garcidueñas-Fimbres, Alicia Larruy-García, Santiago Navas-Carretero, Olga Portoles, Katherine Flores-Rojas, Rocío Vázquez-Cobela, Sangeetha Shyam, María L. Miguel-Berges, J. Alfredo Martínez, Pilar Codoñer-Franch, Mercedes Gil-Campos, Luis A. Moreno, Jordi Salas-Salvadó

**Affiliations:** 1 CIBER. Fisiopatología de la Obesidad y Nutrición (CIBEROBN), Instituto de Salud Carlos III (ISCIII), Madrid, Spain; 2 Universitat Rovira i Virgili, Unitat de Nutrició Humana, Grup ANUT-DSM, Departament de Bioquimica i Biotecnologia, Reus, Spain; 3 Institut d’Investigació Sanitària Pere Virgili (IISPV), Reus, Spain; 4 Growth, Exercise, Nutrition and Development (GENUD) Research Group, Instituto Agroalimentario de Aragón (IA2). Faculty of Health Sciences. Universidad de Zaragoza, Instituto de Investigación Sanitaria de Aragón (IIS Aragón), 50009 Zaragoza, Spain; 5 Metabolism and Investigation Unit, Reina Sofia University Hospital, Maimónides Institute of Biomedicine Research of Córdoba (IMIBIC), University of Córdoba, Córdoba, Spain; 6 Unit of Pediatric Gastroenterology, Hepatology and Nutrition, Pediatric Service, Hospital Clínico Universitario de Santiago, 15706 Santiago de Compostela, Spain; 7 Pediatric Nutrition Research Group, Health Research Institute of Santiago de Compostela (IDIS), Unit of Investigation in Nutrition, Growth and Human Development of Galicia-USC, 15706 Santiago de Compostela, Spain; 8 Center for Nutrition Research, University of Navarra, 31008 Pamplona, Spain; 9 Dept Nutr Food Sci & Physiol, Fac Pharm & Nutr, University of Navarra, 31008 Pamplona, Spain; 10 IdisNA, Navarra Institute for Health Research, Pamplona, Spain; 11 Departament of Preventive Medicine and Public Health. Department of Pediatrics, Obstetrics and Gynecology., University of Valencia, Valencia, Spain

**Keywords:** Food frequency questionnaire, Children, Reproducibly, Validity, Dietary assessment

## Abstract

A reliable food and beverage frequency questionnaire (F&B-FQ) to measure dietary intakes for children across Spain is currently unavailable. Thus, we designed and assessed the reproducibility and relative validity of a new F&B-FQ in 210 Spanish children aged 3–11 years. COME-Kids F&B-FQ contained 125 items to assess the usual diet intake in the past year among children. To explore the reproducibility, caregivers answered COME-Kids F&B-FQ twice over a 15-day period (± 1 week). To evaluate the relative validity, estimates from a third COME-Kids F&B-FQ administered at 1 year of follow-up were compared with the mean estimates from 3-day dietary records (3d-DR) collected at baseline, 6 months, and after 1 year of follow-up. Reproducibility and relative validity of the COME-Kids F&B-FQ in estimating food groups and nutrients were assessed using Pearson (*r*) and intra-class (ICC) correlation coefficients. We used the kappa index to evaluate the agreement in repeat administrations or with the 3d-DR. We used Bland–Altman plots to identify bias across levels of intake. A total of 195 children (105 boys, 90 girls) completed the study. The reproducibility of data estimated from COME-Kids F&B-FQ was substantial with mean *r* and ICC being 0.65 and 0.64 for food groups and 0.63 and 0.62 for nutrients, respectively. Validation assessments comparing the FFQ and 3d-DRs showed *r* = 0.36 and ICC = 0.30 for food groups and *r* = 0.29 and ICC = 0.24 for nutrients. The mean agreement for food group reproducibility and relative validity was 86% and 65%, respectively. These estimates were 85% for reproducibility and 64% for relative validity in the case of nutrients. For reproducibility and relative validity, the overall mean kappa index was 63% and 37% for all food groups and 52% and 27% for nutrients, respectively. Bland–Altman plots showed no specific bias relating to the level of intake of nutrients and several food groups.

*Conclusion*: COME-Kids F&B-FQ showed substantial reproducibility and acceptable relative validity to assess food and beverage intake in Spanish children aged 3 to 11 years. Most children were correctly classified in relation to the intake of food groups and nutrients, and misclassification was unlikely with reference to 3d-DR.

**Table:** 

**What is Known:** *• The estimation of dietary intake in children is complex, especially in large cohorts.* *• The food frequency questionnaire is a well-recognized and the most frequently used method for assessing food consumption.*	
**What is New:** *• A new food and beverage frequency questionnaire including a beverage section and novel plant-based food items has been validated in Spanish children aged 3–11 years.*

**Supplementary Information:**

The online version contains supplementary material available at 10.1007/s00431-023-05220-9.

## Introduction

Establishing healthy eating habits in early childhood is crucial as they are determinants of health and disease in adulthood [1]. However, the estimation of dietary intake in children is complex, especially when dietary intake has to be evaluated in large prospective cohorts. The food frequency questionnaire (FFQ) is a well-recognized and most frequently used method to assess food intake in large population studies evaluating associations between diet and health-related outcomes [2] even in cohorts of children [3, 4] and adolescents [5]. Unfortunately, dietary intake assessments using FFQs are usually subject to systematic and random errors [6, 7] affecting their accuracy and relative validity. However, after energy adjustments, these errors are generally reduced, making FFQ-derived data useful to suitably rank study participants according to dietary consumption. Furthermore, FFQs assess dietary intake without altering routine eating habits and are a relatively low-cost method. Nevertheless, FFQs are only valid for the population for which they were developed [3], as they avoid measurement errors and improve the accuracy of dietary estimations. Thus, in recent years, there have been several FFQ validation studies in specific populations, according to the country of residence, age, and cultural factors, which may influence food consumption [2].

In the last decade, several FFQs have been validated in children from different at different growth stages and cultural contexts including preschoolers from China [8], Malaysia [9], Greece [10], Australia [11], Bangladesh [12], and several European countries [13, 14]. In addition, validations have been performed in pre-adolescents or adolescents from China [15], Vietnam [16], Italy [17], and Brazil [18]. Other validations including a wide age range have also been conducted in South America [19], Denmark [20], and Europe [21].

To the best of our knowledge, in Spain, only three FFQs have been validated in healthy children aged 4–5 years [22], 4–7 years [23], and 7–9 years [24]. These validations have only been performed in children residing in two cities in Spain (Valencia and Navarra), while foods consumed largely vary across different regions of Spain. Additionally, in these FFQs, beverages and fluid items have not been exhaustively considered. Furthermore, nutrient intakes estimated from these Spanish FFQs did not always show an acceptable correlation with estimates using 24-h dietary recalls or dietary records and were validated for a very narrow age range and did not include children aged 3 or those age above 9 years [22–24].

Therefore, the objective of the present study was to assess the reproducibility and relative validity of a food and beverage frequency questionnaire (F&B-FQ) in children aged 3 to 11 years from several Spanish cities.

## Materials and methods

### Design and sample

A F&B-FQ named COME-Kids has been designed to assess the usual dietary and beverage intake. This questionnaire has been derived from a previously validated FFQ in Spain [25]. In order to assess its reproducibility and relative validity, a total of 210 healthy participants aged 3 to 11 years were recruited for the present study (see flowchart in Supplementary material). The objective of the study required a sample size between 100 and 200 participants, in accordance with the recommendation of Willett and Lenhart (1998) [6].

Participants aged 3 to 6 years were recruited from two studies: the CORALS (Children Obesity Risk Assessment Longitudinal Study, https://corals.es) [26] and the MELI-POP (Mediterranean Lifestyle in Pediatric Obesity Prevention) study control group. Participants aged 7 to 11 years old were recruited from the same schools participating in the CORALS study (more information in Supplementary material). Participants for the present study were recruited between March 2019 and November 2019.

The research protocol of this study was approved by all the ethic committees of the centers involved in the study (CEIC Córdoba: Acta 284/Ref. 4155), CEIC Navarra (2019/18), CEIm Institut d’Investigació Sanitària Pere Virgili (CEIm-IISPV 051/2019), CEIC Santiago-Lugo (2019/131), CEIm Hospital Universitario Dr. Peset (CEIm: 9/19), and CEIC Aragón (09/2019), which was conducted following the standards of the Declaration of Helsinki.

All families or caregivers who received a detailed description of the study were asked to fill in and sign the informed consent forms for participation and were given the chance to withdraw the children from the study at any point.

### COME-Kids food and beverage frequency questionnaire

The COME-Kids F&B-FQ is an adapted, semi-quantitative questionnaire with a total of 125 items. The questionnaire was filled in approximately 20 min in a face-to-face interview by trained registered dietitians. For children aged 3 to 9 years, parents or caregivers were asked how often, on average, the participant had consumed the specified serving of each item during the last year. Children aged 10 or above were personally interviewed because they can provide reliable information. Nevertheless, their parents confirmed or helped them provide additional details of the recipes of dishes consumed.

The questionnaire allows for the collection of detailed information on the variety and quantity of food and beverages habitually consumed by children in the last year. COME-Kids F&B-FQ included an item that assesses the frequency of eating in fast food restaurants and 3 open items to document usual food or dietary supplements consumed beyond other items included in the questionnaire (see more details in Supplementary material).

The COME-Kids F&B-FQ was developed in an optically readable form format (Supplementary material), which was scanned using Evaldara^®^ software. The software automatically exports the indicated consumption frequency for the 125 items, which was incorporated into the e-Diet Base URV software [27]. It computes nutrient and food group intake estimates by multiplying the frequency of consumption of each item by the nutrient content of the portion specified in the F&B-FQ. The total energy and nutrient intakes were estimated according to the Spanish CESNID [28] database, the Veggie Base [29] and CELIAC Base [30] for some special food or beverages, or from nutrient facts labels from the food packages. To minimize misreporting, participants who reported energy intake below the 5th percentile or above the 95th percentile from F&B-FQ3 and 3d-DR were excluded from the present analysis [31].

### Reproducibility and relative validity

To explore the COME-Kids F&B-FQ reproducibility, the parents or caregivers answered the F&B-FQ twice (F&B-FQ_1_ and F&B-FQ_2_) over 15 days (± 1 week) period about the children’s usual diet in the past year.

**Fig. 1:**
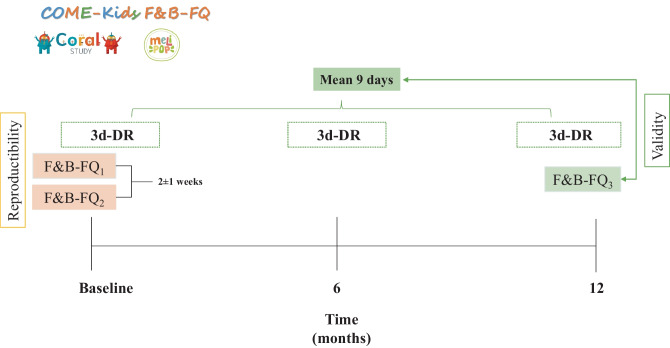
Design of the COME-Kids F&B-FQ reproducibility and relative validity assessments. 3d-DR, 3-day dietary records; F&B-FQ, food and beverage frequency questionnaire

To evaluate the relative validity, a 3-day dietary record (3d-DR) including two weekdays and one holiday (non-consecutive) was used as the reference method. Three 3d-DRs were collected at baseline, 6 months, and 1-year follow-up. Then, the mean daily intake of 9 days was computed. To conduct the validation, a third questionnaire (F&B-FQ_3_) was administered at a 1-year follow-up, which was compared with the mean daily intake computed from three questionnaires of 3d-DR collected over the last year in order to capture the seasonally and intraindividual variation without causing fatigue or lowering motivation to fill out the information. Figure 1 shows the design of the COME-Kids F&B-FQ reproducibility and relative validity assessments. The study procedure is described in the Supplementary material.

### Statistical analysis

Quantitative variables were expressed as median (interquartile range) and qualitative variables as a percentage (*n*). Imputation was required only for missing anthropometric data. To impute the missing data of height (*n* = 5) and weight (*n* = 6), the mean value for weight or height by age according to the growth curves of Hernández et al. was used [32].

To assess the reproducibility and relative validity of the F&B-FQ for food groups and nutrients, the Pearson (*r*) and intra-class (ICC) correlation coefficients were estimated and adjusted by dietary energy intake using the residual method [6]. Additionally, we log-transformed the data (log10) to improve the normality for nutrient and food group consumption. The final analysis used both log transformation and energy adjustment. Guidelines for the statistical interpretation of the ICC are based on values 0.81–1.00 (almost perfect), 0.61–0.80 (substantial), 0.41–0.60 (moderate), 0.21–0.40 (slight), and < 0.21 (poor). The *r* is interpreted as follows: “0” = no association, “1” = positive linear association, and “ −1” = negative linear association [33].

Paired *t*-tests were used to compare the means between F&B-FQ_1_ and F&B-FQ_2_, as well as between F&B-FQ_3_ and the mean intake of 9 days calculated from the three 3d-DR.

Food groups and nutrients from the FFQs and 3d-DR were categorized in quintiles. We then evaluated the agreement as those placed in the same quintile (kappa index) and, additionally, as those placed in the same or adjacent quintiles. We examined Bland–Altman plots for each of the food groups and nutrients to explore the direction of bias across levels of intake and determine the agreement between the F&B-FFQ and 3d-DR.

All statistical analyses were performed using Stata 14 software program (StataCorp), and statistical significance was set at a two-tailed *p*-value < 0.05.

## Results

Of the 210 participants who agreed to participate in the study, 195 children (105 boys, 90 girls) completed the study protocol. A total of 28 participants were excluded as they did not complete the three F&B-FFQ and 3d-DR (Supplementary material). The baseline characteristics of the participants included in the reproducibility and validation studies are shown in Table 1.

**Table 1:** Baseline characteristics of children who participated in the COME-Kids food frequency questionnaire reproducibility and validation studies

	**Reproducibility sample (*n* = 195)**	**Validation sample (*n* = 167)**
Age (years)	6.5 (4.5–9.4)	6.7 (4.5–9.0)
Sex (%) (*n*)		
Boys	54 (105)	50 (84)
Girls	46 (90)	50 (83)
Weight (kg)	23.7 (17.7–32.3)	23.3 (17.8–32.3)
Height (cm)	121 (107–139)	121.5 (107.0–138.4)
BMI (kg/m^2^)	16.3 (15.1–17.4)	16.3 (15.1–17.4)
**Weigh status** (%) (*n*)		
Underweight	13 (26)	14 (23)
Normal	72 (140)	71 (119)
Overweight	11 (22)	11 (19)
Obesity	3.6 (7)	3.6 (6)

Data expressed in median (IQR (p25–p75))

*BMI* body mass index

### Reproducibility

The mean daily food groups consumption, *r* and ICC, percentage of agreement, and grossly misclassified between F&B-FQ_1_ and F&B-FQ_2_ are shown in Table 2. Compared to the F&B-FQ_1_, the consumption of cereals, dairy products, meat or meat products, potatoes, sweetened beverages, and vegetables reported in the F&B-FQ_2_ was significantly lower. The overall *r* and ICC were 0.65 and 0.64, respectively. Similar ICC was found after adjusting by energy. The unadjusted *r* between the first two F&B-FQs for the food groups ranged from 0.40 to 0.99, and the ICC ranged from 0.39 to 0.99. For log-transformed energy-adjusted intakes, the mean *r* and ICC were 0.72 and 0.70, respectively. The mean percentage of agreement for all food groups between the first two F&B-FQs showed that 86% of intakes were classified in the same or adjacent quintiles. The kappa index had an average of 63% for all food groups. The percentage of misclassified intakes ranged from 0 (for fish and seafood, vegetables, nuts, legumes, sweets, chocolate, and sugars) to 7.7% (sugar-free beverages), with an average of < 2%The mean daily nutrient intake coefficients (Pearson and intra-class), percentage of agreement, and grossly misclassified between F&B-FQ_1_ and F&B-FQ_2_ are shown in Table 3. The consumption of protein, calcium, phosphorus, magnesium, potassium, thiamine, riboflavin, niacin, vitamin B6, and cobalamin in the F&B-FQ_2_ was significantly lower than in F&B-FQ_1_. The overall mean *r* and ICC were 0.63 and 0.62, respectively. However, after energy adjustments, slight increases in the coefficients were observed (*r* = 0.66 and ICC = 0.65). For log-transformed intakes, overall *r* and ICC slightly increased to *r* = 0.71 and ICC = 0.70 after energy adjustments. The unadjusted *r* ranged from 0.52 (potassium) to 0.77 (vitamin A) and the ICC from 0.50 (zinc) to 0.77 (monounsaturated fatty acids (MUFA)). The percentage of agreement for nutrient intakes classified in the same or adjacent quintile was 85%. Approximately 52% (kappa index) of nutrients were classified in the same quintile. The average percentage of misclassified nutrients was 1.4%, ranging from 0.5 (fiber, folic acid, and vitamin C) to 2.6% (calcium).

**Table 2:** Assessment of the reproducibility for food group intakes: mean intakes, Pearson and intra-class correlation coefficients, and percentage of agreement between the mean of daily intakes from the first two COME-Kids F&B-FQs (*n* = 195)

**Reproducibility**												
**Food groups (g)**	**F&B-FQ**_**1**_	**F&B-FQ**_**2**_	***p*-value**	**Pearson correlation coefficient**			**Intra-class correlation coefficient**			**% of agreement**	**Agreement (kappa index)**	**Grossly misclassified (%)**
	**Mean (SD)**	**Mean (SD)**		***r***	***r***^**1**^	***r***^**2**^	**ICC**	**ICC**^**1**^	**ICC**^**2**^			
Dairy products	514.5 (284.2)	476.9 (232.0)	**0.02**	**0.66**	**0.71**	**0.80**	**0.65**	**0.68**	**0.79**	84	59	2.6
Eggs	24.0 (8.9)	24.5 (8.7)	0.42	**0.54**	**0.56**	**0.61**	**0.55**	**0.56**	**0.62**	95	95	4.6
Meat and meat products	69.5 (28.9)	65.4 (23.8)	**0.01**	**0.63**	**0.66**	**0.70**	**0.61**	**0.64**	**0.69**	86	49	0.5
Fish and seafood	36.0 (23.5)	34.9 (15.7)	0.46	**0.55**	**0.55**	**0.77**	**0.51**	**0.51**	**0.76**	82	57	0
Vegetables	124.6 (75.4)	87.61 (67.4)	**< 0.01**	**0.79**	**0.79**	**0.68**	**0.69**	**0.69**	**0.50**	83	49	0
Potatoes	36.4 (18.3)	31.7 (19.0)	**0.04**	**0.50**	**0.48**	**0.63**	**0.50**	**0.48**	**0.62**	80	40	1.5
Fresh fruits	196.6 (132.8)	190.4 (155.3)	0.54	**0.53**	**0.57**	**0.78**	**0.52**	**0.57**	**0.78**	83	57	1
Other fruits	5.5 (6.4)	5.4 (6.4)	0.59	**0.77**	**0.74**	**0.74**	**0.77**	**0.74**	**0.74**	85	61	1.0
Nuts	2.7 (3.4)	2.6 (3.3)	0.84	**0.76**	**0.77**	**0.71**	**0.77**	**0.76**	**0.72**	82	54	0
Legumes	15.9 (7.5)	15.7 (7.9)	0.68	**0.65**	**0.66**	**0.70**	**0.66**	**0.65**	**0.70**	88	50	0
Cereals	112.2 (45.6)	91.1 (40.0)	**< 0.01**	**0.63**	**0.61**	**0.61**	**0.56**	**0.52**	**0.51**	79	52	1.0
Oils and fats	27.4 (15.5)	27.5 (15.8)	0.93	**0.75**	**0.75**	**0.77**	**0.75**	**0.74**	**0.77**	85	67	1.5
Pastries and cakes	35.5 (36.7)	37.9 (41.3)	0.36	**0.56**	**0.60**	**0.82**	**0.56**	**0.60**	**0.82**	87	56	1.0
Snacks	11.3 (10.0)	11.4 (9.1)	0.83	**0.66**	**0.63**	**0.77**	**0.66**	**0.62**	**0.77**	84	51	0.5
Sweets, chocolate, and sugars	13.3 (9.0)	13.6 (9.6)	0.45	**0.77**	**0.76**	**0.83**	**0.77**	**0.76**	**0.83**	90	59	0
Precooked food	10.9 (22.4)	12.8 (24.3)	0.21	**0.56**	**0.58**	**0.64**	**0.57**	**0.57**	**0.64**	85	62	3.6
Sauces and seasonings	3.0 (1.9)	3.1 (1.9)	0.48	**0.70**	**0.71**	**0.70**	**0.70**	**0.71**	**0.69**	86	61	1.5
Water	963.4 (341.5)	935.1 (332.5)	0.21	**0.57**	**0.57**	**0.43**	**0.57**	**0.56**	**0.41**	77	63	2.6
Juices	52.1 (73.4)	61.3 (111.3)	0.13	**0.65**	**0.64**	**0.80**	**0.60**	**0.58**	**0.80**	91	61	0.5
Plant-based beverages	8.3 (46.5)	8.6 (46.6)	0.11	**0.99**	**0.99**	**0.95**	**0.99**	**0.99**	**0.95**	99	99	1.5
Sweetened beverages	15.6 (22.6)	11.6 (23.9)	**< 0.01**	**0.60**	**0.60**	**0.64**	**0.60**	**0.59**	**0.62**	76	69	7.2
Sugar-free beverages	3.6 (11.1)	3.8 (10.0)	0.72	**0.72**	**0.71**	**0.73**	**0.71**	**0.71**	**0.73**	92	92	7.7
Coffee, tea, and infusions	5.6 (21.4)	5.4 (19.5)	0.94	**0.40**	**0.39**	**0.74**	**0.39**	**0.39**	**0.74**	94	92	5.6
**Average of correlation coefficients**				0.65	0.65	0.72	0.64	0.64	0.70	86	63	1.9

ICC^2^ correlation coefficients after food group intakes were log-transformed and energy-adjusted

*p*-value from paired *t*-tests. % of agreement, percentage of children classified into the same or an adjacent quintile; agreement kappa index, percentage of children classified into the same quintile; Grossly misclassified (%), percentage of children classified in the opposite quintile. Significant correlations are in bold

F&B-FQ_1_ was determined at baseline. F&B-FQ_2_ was determined at 15 days (± 1 week) period

*r*: unadjusted correlation coefficient; *r*^1^ correlation coefficient using energy-adjusted food group intakes; *r*^2^ correlation coefficients after food group intakes were log-transformed and energy-adjusted. ICC: unadjusted correlation coefficient; ICC^1^ correlation coefficient using energy-adjusted food group intakes

*F&B-FQ* food and beverage frequency questionnaire, *ICC* intra-class correlation coefficients, *r* Pearson correlation

**Table 3:** Assessment of reproducibility for energy and nutrient intakes: mean intakes, Pearson and intra-class correlation coefficients, and percentage of agreement between the mean of daily intakes from the first two COME-Kids F&B-FQ (*n* = 195)

**Reproducibility**												
**Energy and nutrients**	**F&B-FQ**_**1**_	**F&B-FQ**_**2**_	***p*-value**	**Pearson correlation coefficient**			**Intra-class correlation coefficient**			**% of agreement**	**Agreement (kappa index)**	**Grossly misclassified (%)**
	**Mean (SD)**	**Mean (SD)**		***r***	***r***^**1**^	***r***^**2**^	**ICC**	**ICC**^**1**^	**ICC**^**2**^			
Energy (Kcal)	1668.2 (412.3)	1630.2 (393.2)	0.12	**0.62**	-	**-**	**0.62**	-	-	86	51	2.0
Carbohydrates (g)	178.0 (51.3)	174.1 (50.0)	0.26	**0.57**	**0.61**	**0.65**	**0.57**	**0.60**	**0.64**	87	54	1.0
Sugar (g)	88.9 (28.8)	88.1 (33.7)	0.70	**0.54**	**0.62**	**0.75**	**0.54**	**0.60**	**0.74**	89	59	1.0
Fiber (g)	14.4 (4.8)	14.0 (4.7)	0.23	**0.63**	**0.75**	**0.78**	**0.63**	**0.75**	**0.78**	82	50	0.5
Protein (g)	63.4 (16.3)	60.6 (13.4)	**< 0.01**	**0.55**	**0.62**	**0.67**	**0.53**	**0.57**	**0.65**	80	47	1.5
Fat (g)	78.1 (23.3)	76.8 (22.4)	0.32	**0.70**	**0.65**	**0.66**	**0.70**	**0.64**	**0.66**	85	56	1.5
SFA (g)	25.5 (8.3)	25.0 (8.9)	0.36	**0.64**	**0.59**	**0.59**	**0.64**	**0.58**	**0.59**	86	48	2.1
MUFA (g)	35.7 (12.6)	35.2 (11.6)	0.40	**0.77**	**0.76**	**0.74**	**0.77**	**0.76**	**0.74**	86	52	1.0
PUFA (g)	11.1 (3.9)	10.9 (3.8)	0.43	**0.61**	**0.56**	**0.63**	**0.61**	**0.56**	**0.62**	83	50	1.0
Cholesterol (mg)	259.9 (69.4)	253.5 (62.7)	0.16	**0.56**	**0.58**	**0.67**	**0.55**	**0.57**	**0.67**	82	52	1.5
Calcium (mg)	963.3 (357.1)	912.7 (309.3)	**0.02**	**0.60**	**0.68**	**0.72**	**0.61**	**0.65**	**0.70**	83	52	2.6
Phosphorus (mg)	1139.1 (321.7)	1085.5 (273.0)	**< 0.01**	**0.67**	**0.67**	**0.69**	**0.58**	**0.63**	**0.67**	82	41	2.1
Iron (mg)	9.4 (2.5)	9.1 (2.3)	0.07	**0.58**	**0.72**	**0.70**	**0.66**	**0.71**	**0.69**	85	49	1.5
Magnesium (mg)	269.6 (63.3)	260.4 (59.1)	**0.02**	**0.58**	**0.69**	**0.74**	**0.58**	**0.68**	**0.73**	84	51	1.5
Potassium (mg)	2666.8 (700.6)	2560.4 (710.0)	**0.03**	**0.52**	**0.61**	**0.70**	**0.52**	**0.60**	**0.69**	83	54	1.5
Sodium (mg)	2323.6 (710.3)	228.5 (682.0)	0.41	**0.64**	**0.65**	**0.69**	**0.64**	**0.65**	**0.69**	80	46	1.0
Zinc (mg)	8.5 (2.6)	8.2 (2.4)	0.08	**0.50**	**0.57**	**0.65**	**0.50**	**0.55**	**0.64**	81	51	1.5
Thiamin (Vit. B1) (mg)	1.1 (0.3)	1.1 (0.3)	**< 0.01**	**0.70**	**0.74**	**0.76**	**0.70**	**0.72**	**0.74**	86	46	1.5
Riboflavin (Vit. B2) (mg)	1.8 (0.6)	1.7 (0.5)	**< 0.01**	**0.67**	**0.73**	**0.75**	**0.66**	**0.71**	**0.74**	84	49	1.5
Niacin (Vit. B3) (mg)	13.4 (4.5)	12.9 (3.6)	**0.05**	**0.67**	**0.67**	**0.73**	**0.65**	**0.65**	**0.73**	88	55	1.5
Pyridoxine (Vit. B6) (mg)	1.6 (0.5)	1.5 (0.5)	**0.04**	**0.59**	**0.63**	**0.74**	**0.58**	**0.62**	**0.72**	89	56	1.0
Folic acid (Vit. B9) (mg)	247.6 (91.3)	239.1 (87.0)	0.07	**0.72**	**0.74**	**0.79**	**0.72**	**0.73**	**0.78**	90	46	0.5
Cobalamin (Vit. B12) (µg)	4.5 (1.7)	4.2 (1.3)	**< 0.01**	**0.62**	**0.60**	**0.66**	**0.59**	**0.55**	**0.64**	87	50	1.5
Vitamin C (mg)	88.4 (48.0)	89.8 (72.9)	0.72	**0.66**	**0.68**	**0.79**	**0.60**	**0.62**	**0.79**	89	59	0.5
Vitamin A (µg)	718.0 (296.6)	690.1 (302.9)	0.06	**0.77**	**0.78**	**0.77**	**0.76**	**0.78**	**0.77**	89	56	1.0
Vitamin D (µg)	2.7 (2.0)	2.5 (1.4)	0.20	**0.69**	**0.66**	**0.73**	**0.65**	**0.63**	**0.73**	84	55	1.5
Vitamin E (mg)	9.0 (3.3)	8.9 (3.3)	0.59	**0.69**	**0.67**	**0.69**	**0.69**	**0.67**	**0.69**	85	57	1.5
**Average of correlation coefficients**				0.63	0.66	0.71	0.62	0.65	0.70	85	52	1.4

*p*-value from paired *t*-tests. % of agreement, percentage of children classified into the same or an adjacent quintile; agreement kappa index, percentage of children classified into the same quintile; grossly misclassified (%), percentage of children classified in the opposite quintile. Significant correlations are in bold

F&B-FQ_1_ was determined at baseline. F&B-FQ_2_ was determined at 15 days (± 1 week) period. *r*: unadjusted correlation coefficient; *r*^1^ correlation coefficient using energy-adjusted food group intakes; *r*^2^ correlation coefficients after food group intakes were log-transformed and energy-adjusted. ICC: unadjusted correlation coefficient; ICC^1^ correlation coefficient using energy-adjusted food group intakes; ICC^2^ correlation coefficients after food groups intakes were log-transformed and energy-adjusted

*F&B-FQ* food and beverage frequency questionnaire, *ICC* intra-class correlation coefficients, *r* Pearson correlation

### Relative validity

Table 4 shows the average intake of daily food groups, *r*, ICC, percentage of agreement, and items grossly misclassified between the F&B-FQ_3_ and the average of the 3d-DR.

**Table 4:** Assessment of relative validity for food group intakes: mean intakes, Pearson and intra-class correlation coefficients, and percentage of agreement between the mean of daily consumption of food groups from the COME-Kids F&B-FQ at 1-year of follow-up and the average of the three 3d-DR

**Relative validity**	**Assessment**											
**Daily food group intakes (g)**	**F&B-FQ**_**3**_	**3d-DR average**	***p*-value**	**Pearson correlation coefficient**			**Intra-class correlation coefficient**			**% of agreement**	**Agreement (kappa index)**	**Grossly misclassified (%)**
	**Mean (SD)**	**Mean (SD)**		***r***	***r***^**1**^	***r***^**2**^	**ICC**	**ICC**^**1**^	**ICC**^**2**^			
Dairy products	462.4 (218.5)	350.1 (136.5)	**< 0.01**	**0.60**	**0.61**	**0.72**	**0.45**	**0.47**	**0.65**	73	34	0.6
Eggs	26.8 (16.0)	26.6 (15.0)	0.89	**0.11**	0.08	**0.40**	**0.11**	0.09	**0.38**	44	24	17
Meat and meat products	70.4 (22.5)	96.0 (39.9)	**< 0.01**	**0.24**	**0.28**	**0.32**	**0.15**	**0.18**	**0.24**	64	23	3.6
Fish and seafood	35.5 (15.5)	43.7 (24.6)	**< 0.01**	**0.28**	**0.34**	**0.43**	**0.23**	**0.28**	**0.40**	63	26	4.2
Vegetables	86.2 (58.3)	109.7 (55.7)	**< 0.01**	**0.32**	**0.32**	**0.47**	**0.30**	**0.30**	**0.42**	67	30	3.6
Potatoes	31.8 (17.6)	15.0 (16.3)	**< 0.01**	0.07	0.08	0.05	0.05	0.05	0.02	52	19	8.4
Fresh fruits	189.7 (106.0)	171.7 (86.5)	**0.01**	**0.56**	**0.57**	**0.76**	**0.55**	**0.55**	**0.75**	76	38	1.2
Other fruits	5.1 (5.5)	1.3 (5.2)	**< 0.01**	0.07	0.04	0.002	0.06	0.04	0.001	49	20	20
Nuts	3.0 (3.5)	2.1 (4.7)	**< 0.01**	**0.43**	**0.45**	**0.44**	**0.41**	**0.42**	**0.41**	57	25	0.6
Legumes	16.0 (7.0)	21.3 (14.9)	**< 0.01**	**0.30**	**0.32**	**0.37**	**0.21**	**0.22**	**0.33**	57	28	5.4
Cereals	92.8 (37.0)	141.9 (64.8)	**< 0.01**	**0.28**	**0.32**	**0.43**	**0.17**	**0.18**	**0.25**	64	29	2.4
Oils and fats	30.3 (14.0)	17.2 (10.7)	**< 0.01**	**0.40**	**0.39**	**0.35**	**0.24**	**0.23**	**0.22**	64	31	5.4
Pastries and cakes	32.5 (25.5)	35.2 (24.8)	0.12	**0.50**	**0.48**	**0.47**	**0.50**	**0.48**	**0.46**	70	35	1.8
Snacks	11.2 (10.5)	4.7 (6.1)	**< 0.01**	**0.24**	**0.21**	**0.25**	**0.16**	**0.14**	**0.15**	52	21	3.0
Sweets, chocolate, and sugars	13.7 (9.0)	13.2 (12.6)	0.66	**0.39**	**0.38**	**0.56**	**0.37**	**0.34**	**0.51**	74	36	2.4
Precooked food	14.7 (27.4)	56.9 (39.5)	**< 0.01**	**0.27**	**0.27**	**0.18**	**0.14**	**0.14**	**0.08**	63	21	4.8
Sauces and seasonings	3.7 (2.4)	8.2 (7.6)	**< 0.01**	0.10	0.13	**0.20**	0.06	0.06	**0.14**	61	23	4.8
Water	928.9 (369.3)	507.3 (261.1)	**< 0.01**	**0.33**	**0.31**	**0.34**	**0.16**	**0.15**	**0.18**	61	30	4.8
Juices	52.7 (68.5)	23.0 (45.8)	**< 0.01**	**0.52**	**0.53**	**0.39**	**0.43**	**0.43**	**0.32**	56	31	5.4
Plant-based beverages	6.9 (31.8)	6.7 (30.8)	0.91	**0.81**	**0.81**	**0.64**	**0.81**	**0.81**	**0.64**	90	90	10
Sweetened beverages	11.2 (17.1)	10.1 (21.3)	0.48	**0.46**	**0.45**	**0.46**	**0.46**	**0.44**	**0.44**	64	57	6.0
Sugar-free beverages	2.4 (6.9)	0.7 (5.2)	**< 0.01**	**0.36**	**0.35**	**0.38**	**0.33**	**0.33**	**0.29**	90	90	10
Coffee, tea, and infusions	7.6 (30.0)	1.9 (13.3)	**< 0.01**	**0.74**	**0.74**	**0.52**	**0.53**	**0.53**	**0.40**	84	84	16
**Average of correlation coefficients**				0.36	0.37	0.40	0.30	0.30	0.33	65	37	6.2

F&B-FQ_1_ was determined at baseline. F&B-FQ_2_ was determined at 15 days (± 1 week) period. *r*: unadjusted correlation coefficient; *r*^1^ correlation coefficient using energy-adjusted food group intakes; *r*^2^ correlation coefficients after food group intakes were log-transformed and energy-adjusted. ICC: unadjusted correlation coefficient; ICC^1^ correlation coefficient using energy-adjusted food group intakes; ICC^2^ correlation coefficients after food groups intakes were log-transformed and energy-adjusted

*p*-value from paired *t*-tests. % of agreement, percentage of children classified into the same or an adjacent quintile; agreement kappa index, percentage of children classified into the same quintile; grossly misclassified (%), percentage of children classified in the opposite quintile. Significant correlations are in bold

*F&B-FQ* food and beverage frequency questionnaire, *ICC* intra-class correlation coefficients, *r* Pearson correlation

Compared to the F&B-FQ_3_, the 3d-DR showed a significantly lower consumption of several food groups (dairy products, potatoes, fresh and other fruits, nuts, oils and fats, snacks, water, juices, sugar-free beverages and coffee, tea, and infusions) and higher consumption of other groups (meat and meat products, fish and seafood, vegetables, legumes, cereals, precooked food, sauces, and seasonings).

The average coefficients of relative validity for food groups were *r* = 0.36 and ICC = 0.30. When the estimates were adjusted for energy intake, the results were *r* = 0.37 and ICC = 0.30. Unadjusted *r* ranged between 0.07 (potatoes) and 0.81 (plant-based beverages) and ICC between 0.05 (potatoes) and 0.81 (plant-based beverages). For the correlations of log-transformed and energy-adjusted intakes, we observed the average *r* = 0.40 and ICC = 0.33. The correlations for food groups ranged between *r* = 0.002 (other fruits) and 0.76 (fresh fruits) and ICC between 0.001 (other fruits) and 0.75 (fresh fruits). The mean concordance was 65% for classifying food consumption in the same or adjacent quintiles. Overall, 37% of food consumption was classified in the same quintile. The average percentage of misclassified food groups was 6.2% and ranged from 0.6 to 20%.

Table 5 shows the average daily energy and nutrient intake, *r*, ICC, the percentage of agreement, and items grossly misclassified between the F&B-FQ_3_ and the average of the 3d-DRs. The means of the dietary energy and intake of most nutrients from the 3d-DRs were significantly lower than those from F&B-FQ_3_.

**Table 5:** Assessment of relative validity for energy and nutrient intakes: mean intakes, Pearson and intra-class correlation coefficients, and percentage of agreement between the mean of daily intakes from the COME-Kids F&B-FQ at 1-year of follow-up and the average of the three 3-day DR (*n* = 167)

**Relative validity**	**Assessment**											
**Daily intake of energy and nutrients**	**F&B-FQ**_**3**_	**3d-DR average**	***p*-value**	**Pearson correlation coefficient**			**Intra-class correlation coefficient**			**% of agreement**	**Agreement (kappa index)**	**Grossly misclassified (%)**
	**Mean (SD)**	**Mean (SD)**		***r***	***r***^**1**^	***r***^**2**^	**ICC**	**ICC**^**1**^	**ICC**^**2**^			
Energy (Kcal)	1636.4 (281.8)	1579.4 (273.7)	**0.04**	**0.21**	-	**-**	**0.20**	-	-	58	25	6.6
Carbohydrates (g)	167.3 (36.0)	169.5 (32.0)	0.50	**0.17**	**0.34**	**0.38**	**0.17**	**0.34**	**0.37**	62	22	6.0
Sugar (g)	83.5 (23.7)	76.5 (16.1)	**< 0.01**	**0.40**	**0.57**	**0.60**	**0.35**	**0.50**	**0.56**	65	29	3.6
Fiber (g)	14.0 (3.4)	14.4 (3.9)	0.13	**0.42**	**0.49**	**0.53**	**0.41**	**0.49**	**0.53**	66	32	1.8
Protein (g)	61.6 (11.2)	70.2 (14.0)	**< 0.01**	0.13	**0.23**	**0.22**	0.10	**0.15**	**0.14**	59	29	6.0
Fat (g)	80.1 (17.8)	68.9 (16.5)	**< 0.01**	**0.35**	**0.36**	**0.37**	**0.29**	**0.20**	**0.21**	67	26	4.8
SFA (g)	25.5 (6.2)	23.2 (5.5)	**< 0.01**	**0.37**	**0.52**	**0.58**	**0.35**	**0.43**	**0.48**	68	35	4.2
MUFA (g)	37.4 (10.5)	27.9 (8.5)	**< 0.01**	**0.33**	**0.31**	**0.30**	**0.21**	**0.15**	**0.14**	67	27	4.2
PUFA (g)	11.3 (2.9)	9.5 (3.4)	**< 0.01**	**0.44**	**0.33**	**0.29**	**0.37**	**0.26**	**0.19**	66	24	2.4
Cholesterol (mg)	266.6 (78.3)	265.6 (77.7)	0.90	0.13	0.07	**0.17**	**0.13**	0.07	**0.16**	61	28	6.6
Calcium (mg)	900.1 (251.9)	761.6 (181.5)	**< 0.01**	**0.42**	**0.52**	**0.53**	**0.34**	**0.40**	**0.43**	68	26	1.8
Phosphorus (mg)	1097.5 (233.6)	1051.7 (209.8)	**0.03**	**0.24**	**0.32**	**0.34**	**0.23**	**0.31**	**0.33**	64	28	4.2
Iron (mg)	9.1 (1.8)	9.3 (2.4)	0.34	**0.20**	**0.18**	**0.20**	**0.20**	**0.16**	**0.19**	56	25	2.4
Magnesium (mg)	258.4 (45.4)	230.3 (51.8)	**< 0.01**	**0.35**	**0.50**	**0.53**	**0.30**	**0.38**	**0.39**	64	33	4.8
Potassium (mg)	2538.4 (482.2)	2315.3 (466.5)	**< 0.01**	**0.27**	**0.44**	**0.46**	**0.24**	**0.38**	**0.39**	59	26	4.8
Sodium (mg)	2385.6 (640.3)	2388.6 (686.0)	0.96	**0.35**	**0.35**	**0.34**	**0.35**	**0.35**	**0.34**	63	25	1.8
Zinc (mg)	8.2 (1.8)	7.0 (1.5)	**< 0.01**	0.13	0.13	0.13	**0.11**	**0.09**	**0.09**	55	21	6.0
Thiamin (Vit. B1) (mg)	1.1 (0.2)	1.1 (0.3)	0.17	0.14	**0.31**	**0.29**	**0.13**	**0.28**	**0.27**	62	28	9.6
Riboflavin (Vit. B2) (mg)	1.6 (0.4)	1.6 (0.6)	0.78	**0.16**	0.11	**0.23**	**0.15**	0.10	**0.22**	64	22	1.8
Niacin (Vit. B3) (mg)	12.7 (2.8)	15.8 (4.5)	**< 0.01**	**0.27**	**0.41**	**0.40**	**0.18**	**0.23**	**0.25**	59	23	4.2
Pyridoxine (Vit. B6) (mg)	1.5 (0.3)	1.6 (0.4)	**0.01**	**0.29**	**0.43**	**0.46**	**0.27**	**0.39**	**0.42**	65	24	4.8
Folic acid (Vit. B9) (mg)	237.0 (61.0)	210.2 (64.5)	**< 0.01**	**0.42**	**0.51**	**0.57**	**0.39**	**0.46**	**0.49**	71	31	1.2
Cobalamin (Vit. B12) (µg)	4.4 (1.4)	5.1 (3.3)	**0.01**	**0.17**	0.12	**0.22**	0.12	0.08	**0.19**	62	20	5.4
Vitamin C (mg)	86.4 (43.0)	77.7 (34.6)	**0.01**	**0.49**	**0.52**	**0.55**	**0.46**	**0.50**	**0.54**	74	41	3.6
Vitamin A (µg)	693.4 (245.1)	591.2 (200.8)	**< 0.01**	**0.26**	**0.28**	**0.32**	**0.23**	**0.25**	**0.28**	59	28	4.2
Vitamin D (µg)	2.5 (1.3)	3.5 (3.7)	**< 0.01**	**0.21**	**0.23**	**0.24**	**0.12**	**0.13**	**0.21**	61	27	3.6
Vitamin E (mg)	9.3 (2.7)	6.7 (2.4)	**< 0.01**	**0.41**	**0.39**	**0.41**	**0.27**	**0.23**	**0.22**	69	29	2.4
**Average of correlation coefficients**				0.29	0.34	0.37	0.24	0.27	0.30	64	27	4.2

F&B-FQ_1_ was determined at baseline. F&B-FQ_2_ was determined at 15 days (± 1 week) period. *r*: unadjusted correlation coefficient; *r*^1^ correlation coefficient using energy-adjusted food group intakes; *r*^2^ correlation coefficients after food group intakes were log-transformed and energy-adjusted. ICC: unadjusted correlation coefficient; ICC^1^ correlation coefficient using energy-adjusted food group intakes; ICC^2^ correlation coefficients after food groups intakes were log-transformed and energy-adjusted

*p*-value from paired *t*-tests. % of agreement, percentage of children classified into the same or an adjacent quintile; agreement kappa index, percentage of children classified into the same quintile; grossly misclassified (%), percentage of children classified in the opposite quintile. Significant correlations are in bold

*F&B-FQ* food and beverage frequency questionnaire, *ICC* intra-class correlation coefficients, *r* Pearson correlation

The average *r* and ICC of the relative validity for nutrients were 0.29 and 0.24, respectively. Results after adjustment for energy were *r* = 0.34 and ICC = 0.27. Energy-adjusted *r* ranged from 0.11 (riboflavin) to 0.52 (saturated fatty acid (SFA)) and for ICC from 0.07 (cholesterol) to 0.46 (folic acid). The average log-transformed values for *r* and ICC after energy adjustments were 0.37 and 0.30, respectively. Approximately 64% of daily energy and nutrient intakes were classified in the same or adjacent quintiles. Approximately 27% of these intakes were classified in the same quintile. The average percentage of misclassified nutrients was 4.2% and ranged from 1.8 (fiber) to 9.6% (thiamin).

**Fig. 2:**
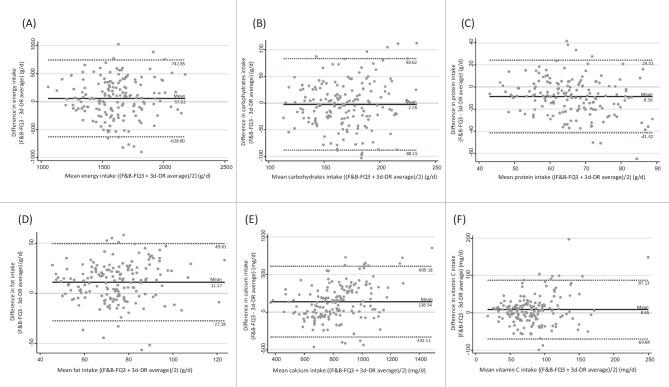
Bland–Altman plots showing the relationship between the mean and the differences in the daily intake of **A** energy, **B** carbohydrates, **C** protein, **D** total fat, **E** calcium, and **F** vitamin C with the mean of the COME-Kids F&B-FQ3 and the average of the three 3d-DR. Lines are the mean difference (—), and the lower and upper 95% show the limits of agreement (- - -). F&B-FQ, food and beverage frequency questionnaire; F&B-FQ_3_ was determined at 1 year of follow-up; 3d-DR average, average of the 3-day dietary records

Figure 2 shows the relationship between the average and differences between the daily intakes of dietary energy and several nutrients and the mean of the F&B-FQ3 and the average of the three 3d-DR (Bland–Altman plots). The analysis confirmed that discrepancies between F&B-FQ3 and 3d-DR were equally likely in both directions. Only protein intake was underestimated by an average of 9 g/day. Mean daily fat (11 g/day) and calcium (9 mg/day) intakes were overestimated by the F&B-FQ_3_ in relation to the 3d-DR. Differences in the intake estimates between the two instruments were not related to the level of consumption. Bland–Altman plots for all food groups and nutrients are shown in the Supplementary material. Based on the average discrepancy (bias) and the limits of agreement, the Bland–Altman plots showed a good agreement between COME-Kids F&B-FQ and 3d-DR in cereals, eggs, fish or seafood intake, fresh fruit, meat or meat products, nuts, pastries and cakes intake, sauces and seasonings, sweets, chocolate and sugars, and vegetable intake. The data from these food groups exhibited relatively narrowed limits of agreement and a centered mean difference line (close to zero), indicating minimal systematic differences between the two questionnaires. However, legumes, juice and other fruits, oils and fats, potatoes, precooked food, snacks, and water exhibited considerable variability in their differences, suggesting a potential bias due to the wide dispersion of the data and the limits of agreement.

The intake differences between COME-Kids F&B-FQ and 3d-DR show a good agreement in cereals, fish or seafood, fresh fruits, meat or meat products, pastries and cakes, sauces and seasonings, and vegetables (< 1 portion/day) and eggs, nuts, and sweets (< 1 g/day). However, poor agreement was observed in the dairy products (almost a half portion/day), juices (due to added sugar), legumes, potatoes, snacks and precooked foods (high spread and low bias), oils and fats, and water (> 1 portion/day).

## Discussion

This validation study supported the use of the 125-item COME-Kids F&B-FQ as an acceptable method for dietary assessment in preschool and school children aged 3–11 years from several Spanish cities. Furthermore, it showed an acceptable reproducibility for most of the food groups and nutrients and a relative validity in comparison with the three 3d-DRs.

In general, epidemiological evidence has rarely considered the intake of water and beverages, and to date, no FFQ has been validated for this purpose in children. It is the first FFQ including a beverage section, which showed acceptable levels of reproducibility and relative validity in relation to fluid intake. The importance of estimating fluid intake, specifically in children, is increasingly recognized as hydration status affects memory and cognitive performance [34, 35].

Additionally, with rapid changes in the food market [36], it is important to consider novel (whole grain cereals, lactose-free milk, isotonic and energy beverages) and emerging plant-based beverages (rice, oat, almond, and soy drinks) items which are becoming more popular. Regarding plant-based beverages, COME-Kids F&B-FQ showed the highest coefficients of reproducibility and agreement and a moderate relative validity.

In addition, especially among children prone to consume high amounts of added sugar, it is imperative that a distinction between foods with and without added sugar (i.e., dairy, beverages, and breakfast cereals) has to be made. Therefore, COME-Kids F&B-FQ, which included an extended number of food items, is more likely to assess Spanish children’s diet accurately.

The delicate desired balance between having an exhaustive estimation and reducing respondent burden guided the number of items in FFQ. The number of food items in previous validated FFQs for children ranged from 17 [11] to 183 [20] items; meanwhile, Spanish-validated FFQs ranged from 105 [22] to 138 [23] items for preschool children, and about 46 items were specific for school-aged children [24]. In this sense, COME-Kids F&B-FQ which included 125 items is adequately comprehensive without being extremely limited or extensive. Given the results of the study, it is highly likely that COME-Kids F&B-FQ achieved the right balance between being sufficiently complete and avoiding respondent fatigue to ensure reliability.

The present validation study is in line with previous studies, in which four weighed 3d-DR [23], three 24-h dietary recall (24-h recall) [22, 24], or 7-day estimated dietary records [37] were also used as a reference. Even though it has been well recognized that the 3d-DR method may lead to misreporting [38], it is important to highlight that the relative validity agreement across the same or adjacent quintiles was in an acceptable range for food groups and nutrient intakes, indicating that gross misclassification was very unlikely.

Regarding correlation coefficients to assess the reproducibility of food group consumption in children, the literature is scarce [20, 22–24, 39]. Taking into consideration other European studies, our correlation coefficients are higher than those obtained in Danish children [20] and Flemish preschoolers [39]. Moreover, those corresponding to the intake of dairy products, eggs, vegetables, potatoes, fruit, legumes, oils and fats, sweets, chocolates, and sugars were higher than those observed in previous Spanish studies in preschool children that were log-transformed and adjusted by energy [22–24]. The higher reproducibility of dietary estimates from COME-Kids F&B-FQ could be due to the sampling of participants from several provinces of Spain and the items in this FFQ better accounting for regional variations in gastronomic culture. Regarding the correlation coefficients to assess the reproducibility of nutrient intake in children, the positive correlations indicated moderate to high consistency between COME-Kids F&B-FQs. Thus, similar to other efforts to validate FFQs developed in previous European projects [37] and in Asian preschoolers [9], our study showed the relevance of conducting local adaptations to improve the assessments.

It is noted that there were significant discrepancies between COME-Kids F&B-FQ and 3d-DR for estimated intakes of a few food groups. Consumption of dairy, snacks, and potatoes was overreported, while juices, legumes, and precooked food were under-reported in the COME-Kids F&B-FQ in comparison to 3d-DR. It is likely that the intakes of more frequently consumed foods are overestimated and those of less frequently consumed foods underestimated by the parents/caregivers while reporting using an FFQ. This discrepancy may be avoided in the 3d-DR where the parent/caregiver records the actual intake. In contrast, oils and fats and water tend to be overlooked when parents/caregivers are capturing dietary data using 3d-DR. However, when these are itemized in the FFQ, they tend to not forget and report their consumption. Hence, the COME-Kids F&B-FQ provides an adequate estimation of energy and macronutrients and nutrients of concern such as fat, salt, and sugar intakes.

This study has several strengths that deserve to be mentioned for efforts made to improve the quality of dietary data and enhance our ability to collect complex information. First, we have standardized and automated data collection and transcription procedures using the e-Diet Base software. Second, newer techniques, such as optical scanning, and the use of food pictures helped dietitians to ensure the accuracy of reporting by parents/caregivers, simplify their reporting, and minimized the load in the fieldwork of researchers. More importantly, our target population consumed several beverages including water, plant-based drinks, juices, and sugar-free and sugar-sweetened beverages. The currently available FFQs for measuring dietary intakes in Spanish children do not include many of these items. Most current FFQs are restricted to capturing sugar-sweetened beverages or orange juice. The COME-Kids F&B-FQ, therefore, provides a better estimate of beverage intake in this target population. In fact, it is as good as 3d-DR for estimating sugar-free and sugar-sweetened beverages and plant-based drinks and better captures water intakes. Given the health implications of beverage consumption, specifically in this age group [22–24], therefore, COME-Kids F&B-FQ has filled a much-needed method gap in the area.

Nevertheless, some limitations have to be acknowledged. First, although the interviewers and the parents/caregivers were well-trained, some sources of measurement error in assessing dietary intakes cannot be ruled out, and for the moment, the estimation of intakes could not be validated by biochemical biomarkers. However, it is important to highlight that studies using biomarkers as the only reference method [40] or combined with 24-h recall or dietary records [18] did not find better correlations than those observed in the present study. Furthermore, some authors consider that the use of biomarkers may not be always an appropriate reference method for comparison given that blood concentrations depend on absorption and metabolism and may not always reflect dietary intake [40]. Secondly, in this study, some visits (10.3%) occurred during the confinement period due to COVID-19 pandemic. Data from these visits were collected using video conferences or telephone calls. However, sensitivity analyses stratifying according to the type of visit (in-person and by video conference/calls) showed similar results, indicating that the visit type may have no impact on the results.

## Conclusion

The COME-Kids F&B-FQ is a valid tool to assess food and nutrient intake in children aged between 3 and 11 years. The intakes of most food groups and nutrients estimated using this FFQ were correctly classified, and misclassification was unlikely. Therefore, the COME-Kids F&B-FQ could be a useful tool to help improve dietary assessment in pediatric epidemiological studies in Spain.

### Supplementary Information

Below is the link to the electronic supplementary material.

Supplementary file1 (PDF 1543 KB)

## Data Availability

The datasets generated and analyzed during the current study are not publicly available due to data regulations and for ethical reasons, considering that this information might compromise research participants’ consent because our participants only gave their consent for the use of their data by the original team of investigators. However, collaboration for data analyses can be requested by sending a letter to the CORALS Steering Committee (estudiocoral@corals.es). The request will then be passed to all the members of the CORALS Steering Committee for deliberation.

## Abbreviations

3d-DR: 3-Day dietary record
F&B-FQ: Food and beverage frequency questionnaire
FFQ: Food frequency questionnaire
ICC: Intra-class correlation coefficient
MUFA: Monounsaturated fatty acids
*r*: Pearson correlation coefficient
