# Supplementary material for: Reproducibility and relative validity of a semi-quantitative food and beverage frequency questionnaire for Spanish children aged 3 to 11 years: the COME-Kids F&B-FQ
Source: Eur J Pediatr. 2023 Oct 6;182(12):5577–89. doi: 10.1007/s00431-023-05220-9 (PMC10746573; doi:10.1007/s00431-023-05220-9)
Supplement: Supplementary file 1 — Supplementary file1 (PDF 1543 KB) [file 431_2023_5220_MOESM1_ESM.pdf]

Supplemental material

Index

Methods 2

Sample 2

COME-Kids food and beverages frequency questionnaire. 2

Study procedure 3

Flow chart 5

COME-KIDS Questionnaire 6

Bland-Altman plots for all food groups and nutrients 9

Methods

Sample

Briefly, CORALS is a prospective ongoing multicentre cohort study conducted in preschool children from 7 Spanish cities, which aims to identify and assess the risk factors that contribute to the development of obesity in childhood. MELIPOP is a multicentre parallel, randomized controlled intervention trial including children aged 3 to 6 years-old at risk of obesity. MELIPOP aims to assess whether a healthy lifestyle intervention during childhood, based on the promotion of a Mediterranean diet and regular physical activity decreases the incidence of obesity at long term.

COME-Kids food and beverages frequency questionnaire.

The COME-Kids F&B-FQ was designed based on the adaptation of the validated adult FFQ used in the PREDIMED study (PREvención con DIeta MEDiterránea) [1], considering also the amounts and types of food specifically consumed by children aged between 3 and 11 years.

From the original version, which included 146 items, relevant modifications were made especially in relation to the serving size of usual consumption and the number of items of vegetables and fruits that were reduced in a few categories (PREDIMED version n=35 vs COME-Kids version n=16), grouping them according to their nutritional composition and avoiding potential overestimation due to a high number of items [2]. Some food items more commonly consumed by children (for instance: jellybeans, popcorn, bread sticks) and food items that have gained popularity recently (plant-based beverages, lactose-free milk; energy drinks) were included in the new questionnaire. In addition, in contrast to the original version, the present questionnaire separately captures yogurt and cereals with

and without added sugar and their whole or refined versions to facilitate discrimination in their nutrient content.

The items corresponding to alcoholic beverages from the original version were removed and a beverage section was added including those that are usually consumed by children, including bottled and tap water.

The frequency response options consisted as follows: never; 1-3 times per month; 1 time per week; 2-4 times per week; 5-6 times/week; 1 time per day; 2-3 times per day; 4-6 times per day and more than 6 times per day.

Study procedure

For recruitment, parents or caregivers were invited to participate through a letter sent by the staff of each school informing them about the study. Those who accepted to participate were interviewed by telephone or in-person and asked to attend the baseline visit.

Registered dietitians from each recruitment centre attended group training sessions to harmonize criteria and minimize potential inter-interviewer bias, recognized as a major contributor to variation in the data collected [3].

Baseline visit: Face-to-face, trained registered dietitians administered the F&B-FQ_1 to the participant's parents/caregivers in case of children <10 years old and handed over the first 3d-DR. For that, they were instructed to provide a detailed description of the type of consumed food, recipes, brand names and servings in grams or household measures (spoon, glass, cups, etc.). In addition, parents/caregivers were also advised to take pictures of the plate prior to food intake, to improve the accuracy of the amount of food and beverage consumed.

When registered dietitians explained the procedure to complete the 3d-DR, they emphasized that the objective of this record was to capture the children's usual diet so they were requested not to change the child's standard diet. Furthermore, to estimate the

food intake from different days of the week and to avoid systematic errors arising from repeated intakes on the same days of the week, a sequence of weekly days was indicated to the parents in order to collect the dietary records according to the participant's coding. Parents or caregivers also completed self-administered questionnaires assessing sociodemographic and education data of the family. The weight and height of participants were measured by trained registered dietitians. Weight status was estimated according to body mass index (BMI) and categorized in underweight, normal weight, overweight or obesity according to the cut-off points defined by Cole et al [4].

Visit at 2 ± 1 week and at 6 months: The second questionnaire (F&B-FQ_2) was administered as well as the instructions to complete the second 3d-DR provided. Around one week before the 6-month visit, parents/caregivers were reminded to complete this 3d-DR for the next scheduled visit.

One-year visit. Around one week before the one-year visit parents/caregivers were contacted again and reminded to complete the 3d-DR for the scheduled visit. The F&B-FQ_3 was administered, and the third 3d-DR was accurately reviewed as previously described.

Given the COVID-19 pandemic, those visits that concurred with the lockdown period were facilitated through videoconference or telephone calls being the 3d-DR template sent by e-mail to be completed.

1. Fernández-Ballart JD, Piñol JL, Zazpe I, et al (2010) Relative validity of a semi-quantitative food-frequency questionnaire in an elderly Mediterranean population of Spain. Br J Nutr 103:1808–1816. https://doi.org/10.1017/S0007114509993837
2. Willet W (1998) Reproducibility and validity of food-frequency questionnaires, 2nd editio. New York: Oxford University Press
3. Gavrieli A, Trichopoulou A, Valsta LM, et al (2019) Identifying sources of measurement error in assessing dietary intakes - Results of a multi-country ring-trial. Nutr Metab Cardiovasc Dis 29:127–134. https://doi.org/10.1016/J.NUMECD.2018.10.011
4. Cole TJ, Lobstein T (2012) Extended international (IOTF) body mass index cut-offs for thinness, overweight and obesity. Pediatr Obes 7:284–294. https://doi.org/10.1111/j.2047-6310.2012.00064.x

Flow chart

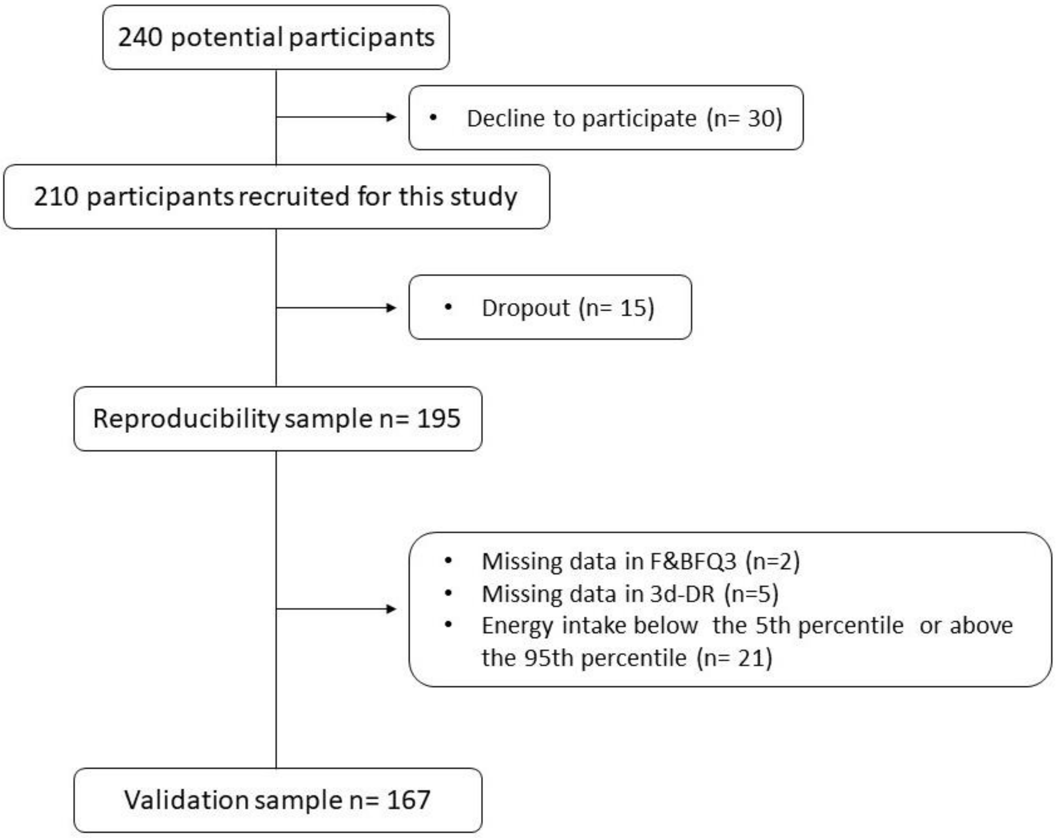

Figure 2. Flow chart of the study sample.

COME-KIDS Questionnaire

Coräl STUDY

meli POP

Frequency food consumption for children from 3 to 11 years-old

Center	
0	0
1	1
2	2
3	3
4	4
5	5
6	6
7	7

Participant		
0	0	0
1	1	1
2	2	2
3	3	3
4	4	4
5	5	5
6	6	6
7	7	7
8	8	8
9	9	9

Visit	
0	0
1	1
	2
	3
	4
	5
	6
	7
	8
	9
1	0

Current data					
Día		Mes		Año	
0	0	0	0	0	0
1	1	1	1	1	1
2	2		2	2	2
3	3		3		3
	4		4		4
	5		5		5
	6		6		6
	7		7		7
	8		8		8
	9		9		9

Please check only one option for each food.

		For each food, check the box indicating the frequency of consumption on **average over** the **past year**. The summer/winter variation should also be taken into account. For example, if you eat ice-cream 4 times/week only during the 3 summer months, the average consumption per year is 1/week.	AVERAGE CONSUMPTION OVER THE PAST YEAR								
			NEVER OR ALMOST NEVER	TIMES PER MONTH	TIMES PER WEEK			TIMES PER DAY			
				1-3	1	2-4	5-6	1	2-3	4-6	6+
I. DAIRY PRODUCTS	1	Whole milk (1 glass 200cc)									
	2	Semi-skimmed milk (1 glass 200cc)									
	3	Skimmed milk (1 glass 200cc)									
	4	Condensed milk (1 tablespoon)									
	5	Lactose-free milk (1 glass 200cc)									
	6	Cream or milk cream (1 tablespoonful)									
	7	Milk shakes (1 brick 200cc)									
	8	Natural unsweetened yogurt (1u, 125g)									
	9	Natural sweetened yogurt (1u, 125g)									
	10	Other whole-fat yogurt (1u, 125g)									
	11	Skimmed yogur (1u, 125g)									
	12	Fermented milk (100 cc)									
	13	Petit suisse (1u, 55g)									
	14	Ricotta or curd (1 tablespoon)									
	15	Cheese in portions or creamy (1 portion, 25g)									
	16	Other cheeses: cured, semi-cured (Manchego, Emmental...) (25g)									
	17	Cottage cheese (Burgos, goat...) (1/2 tub, 30g)									
	18	Custard, flan, pudding (1, 130cc)									
	19	Ice-cream (1 cone)									

		One plate or portion of 40-70g, except when another quantity is indicated.	NEVER OR ALMOST NEVER	TIMES PER MONTH	TIMES PER WEEK			TIMES PER DAY			
				1-3	1	2-4	5-6	1	2-3	4-6	6+
II. EGGS, MEATS, FISH	20	Chicken eggs (1 unit)									
	21	Chicken or turkey WITH skin (1 serving or piece)									
	22	Chicken or turkey WITHOUT skin (1 serving or piece)									
	23	Beef or veal (1 serving)									
	24	Pork (1 serving)									
	25	Lamb (1 serving)									
	26	Rabbit (1 serving)									
	27	Liver (beef, pork, chicken) (1 portion)									
	28	Other viscera (brains, heart, sweetbreads) (1 serving)									
	29	Serrano ham or shoulder ham (1 thin slice, 15g)									
	30	York ham, cooked ham (1 thin slice, 15g)									
	31	Processed meats: sausage, chorizo, blood sausage, mortadella, frankfurt, sobrasada... (15g)									
	32	Pâté, foie gras (1 spoonful)									
	33	Hamburger (1 unit, 50g) meatballs (2 units)									
	34	Bacon (1 slice, 30g)									
	35	White fish: halibut, sole, hake, whiting... (1 portion)									
	36	Oily fish: sardines, tuna, bonito, mackerel, salmon... (1 piece or portion)									
	37	Salted fish: cod, salted fish... (1 portion, 30g dry)									
	38	Oysters, clams, mussels and similar (3 units)									
	39	Squid, octopus, cuttlefish (1 serving, 40-70g)									
	40	Crustaceans: shrimps, prawns, langoustines, crayfish... (2-3 pieces, 30-40g)									
	41	Fish by-products: crab sticks (surimi) (2 pieces)									
	42	Fish and seafood in oil: sardines, anchovies, bonito, tuna... (1/2 can, 25g)									

Please check only one option for each food.

			AVERAGE CONSUMPTION OVER THE PAST YEAR								
		One 50-80g plate or portion, except when another quantity is indicated.	NEVER OR ALMOST NEVER	TIMES PER MONTH	TIMES PER WEEK			TIMES PER DAY			
				1-3	1	2-4	5-6	1	2-3	4-6	6+
III.VEGETABLES	43	Vegetables A* (chard, spinach, endive, lettuce,...)									
	44	Vegetables B** (pumpkin, beans, tomato, carrots,...)									
	45	Garlic (1/2 clove)									
	46	Mushrooms, chanterelles									
	47	Baked or boiled potatoes									
	48	Homemade fried potato chips (1 portion, 80g)									
	49	Potato crisps (1 bag, 50g)									
	50	Vegetable soup (1 glass, 200cc)									

* A vegetables: Chard, artichoke, celery, watercress, broccoli, zucchini, cardoon, cauliflower (frozen or raw), endive, endive, white asparagus (canned or peeled and raw), spinach (raw or frozen), fennel, lettuce, cucumber, bell bell pepper, radish, bean sprouts.

** B vegetables: Eggplant, squash, white onion, Brussels sprouts, red cabbage, cabbage, green beans (raw and frozen), turnip, hearts of palm (canned), red bell pepper, leek, tomato, carrot.

			AVERAGE CONSUMPTION OVER THE PAST YEAR								
		One 100g piece or portion, except where otherwise indicated.	NEVER OR ALMOST NEVER	TIMES PER MONTH	TIMES PER WEEK			TIMES PER DAY			
				1-3	1	2-4	5-6	1	2-3	4-6	6+
IV. FRUITS	51	Citrus fruits (tangerine, orange, grapefruit)									
	52	Banana (1unit)									
	53	Fresh fruits (all except banana or citrus fruits)									
	54	Kiwi (1 piece, 100g)									
	55	Fruit in syrup (peach, pear) (1/2 piece)									
	56	Dates, dried figs, raisins, prunes (20g)									
	57	Olives (10 units)									
	58	Nuts (almonds, hazelnuts, walnuts) (15-20g) 1 handful the size of the child's hand									

			AVERAGE CONSUMPTION OVER THE PAST YEAR								
		1/2 small cup (uncooked weight, 40g)	NEVER OR ALMOST NEVER	TIMES PER MONTH	TIMES PER WEEK			TIMES PER DAY			
				1-3	1	2-4	5-6	1	2-3	4-6	6+
V.LEGUMES AND CEREALS	59	Legumes (lentils, white beans, chickpeas)									
	60	Peas									
	61	White bread (loaf, baguette...) (1 slice, 25g)									
	62	White bread, sandwich loaf (1 piece, 25g)									
	63	Breadsticks, donuts, bagels and similar (3-4 pieces or 1 bagel)									
	64	Brown or whole-wheat bread (1 slice, 25g)									
	65	Unsweetened breakfast cereals (30g)									
	66	Sweetened breakfast cereals (30g)									
	67	Chocolate-filled sweetened breakfast cereals (30g)									
	68	Whole grain cereals: muesli, oat flakes, all-bran (30g)									
	69	White rice (40g raw)									
	70	Brown rice (40g raw)									
	71	Pasta: noodles, macaroni, spaghetti... (40g raw)									
	72	Whole-wheat pasta (40g raw)									
	73	Pizza (60-80g portion)									

			AVERAGE CONSUMPTION OVER THE PAST YEAR								
		How often do you consume...?	NEVER OR ALMOST NEVER	TIMES PER MONTH	TIMES PER WEEK			TIMES PER DAY			
				1-3	1	2-4	5-6	1	2-3	4-6	6+
	74	In fast food restaurants									

Please check only one option for each food.

			AVERAGE CONSUMPTION OVER THE PAST YEAR								
		**One tablespoon or individual serving. For frying, spreading, dipping in bread, dressing or for salads, use in total:**	NEVER OR ALMOST NEVER	TIMES PER MONTH	TIMES PER WEEK			TIMES PER DAY			
				1-3	1	2-4	5-6	1	2-3	4-6	6+
VI. OILS AND FATS	75	Olive oil (10cc, 1 tablespoonful)									
	76	Virgin olive oil (10cc, 1 tablespoonful)									
	77	Pomace olive oil (10cc, 1 tablespoonful)									
	78	Corn oil (10cc, 1 tablespoonful)									
	79	Sunflower oil (10cc, 1 tablespoonful)									
	80	Soybean oil (10cc, 1 tablespoonful)									
	81	Mixture of the above (10cc, 1 tablespoonful)									
	82	Margarine (single serving, 12g)									
	83	Butter (single serving, 12g)									

		AVERAGE CONSUMPTION OVER THE PAST YEAR	NEVER OR ALMOST NEVER	TIMES PER MONTH	TIMES PER WEEK			TIMES PER DAY			
				1-3	1	2-4	5-6	1	2-3	4-6	6+
VII. PASTRIES AND CAKES	84	Biscuits or plain cookies (3 units, 30g)									
	85	Whole-wheat or fiber cookies (3 units, 30g)									
	86	Chocolate cookies (3 units, 30g)									
	87	Croissant, sponge cake or other pastries ... (1 piece, 30g)									
	88	Donut (1 piece)									
	89	Cupcakes (1 piece)									
	90	Cakes (1, 50g)									
	91	Churros, porras and similar (1 unit, 20-30g)									
	92	Chocolates (15g)									
	93	Marzipan (1/2 unit, 45g)									

		AVERAGE CONSUMPTION OVER THE PAST YEAR	NEVER OR ALMOST NEVER	TIMES PER MONTH	TIMES PER WEEK			TIMES PER DAY			
				1-3	1	2-4	5-6	1	2-3	4-6	6+
VIII. MISCELLANEOUS	94	Croquettes, dumplings, pre-cooked food (1 unit, 20-30g)									
	95	Soups and creams in sachets (1 dish)									
	96	Mustard (1 dessert spoon)									
	97	Commercial mayonnaise (1/2 tablespoon = 10g)									
	98	Fried tomato sauce, ketchup (1 teaspoon)									
	99	Salt (1 pinch)									
	100	Iodized salt (1 pinch)									
	101	Soluble cocoa powder (1 dessert spoon)									
	102	Sugar (1 dessert spoon, 5g)									
	103	Honey/jam (1 tablespoon, 15g)									
	104	Cocoa butter, Nutella or similar (1 dessert spoon, 10g)									
	105	Snacks - other than potato chips: doritos, popcorn, nachos... (1 bag, 50g)									
	106	Sweets, lollipops, jelly beans .... 2-3 units (10-15g)									
	107	Other frequently consumed foods									
	108										
	109										

		Please check only one option for each beverage.	AVERAGE CONSUMPTION OVER THE PAST YEAR								
			NEVER OR ALMOST NEVER	TIMES PER MONTH	TIMES PER WEEK			TIMES PER DAY			
				1-3	1	2-4	5-6	1	2-3	4-6	6+
IX. BEVERAGES	110	Tap water (1 glass, 200cc)									
	111	Bottled water (1 glass, 200cc)									
	112	High sodium sparkling water (1 glass, 200 cc)									
	113	Flavored water (1 glass, 200 cc)									
	114	Fresh orange juice (1 glass, 200cc)									
	115	Fresh juices of other fruits (1 glass, 200 cc)									
	116	Bottled or canned fruit juices (1 glass, 200 cc)									
	117	Nectars (200cc)									
	118	Plant-based beverage (soy beverage, almond drink...) (1 glass, 200cc)									
	119	Carbonated beverages with sugar: cola drinks, lemonades, tonics... (1 bottle, 200cc)									
	120	Low-calorie carbonated beverages, light drinks (1 bottle, 200cc)									
	121	Coffee (1 small cup, 100 cc)									
	122	Tea (1 cup, 200 cc)									
	123	Other infusions (1 cup, 200 cc)									
	124	Isotonic beverages (1 glass, 200 cc)									
	125	Energy beverages (1 glass, 200 cc)									

If during the past year you took vitamins and/or minerals (including calcium) or special dietary products (bran, evening primrose oil, milk with omega-3 fatty acids, flavonoids, etc.), please indicate the brand and how often you took them:

	Brand names of vitamin or mineral supplements or dietary products	AVERAGE CONSUMPTION OVER THE PAST YEAR								
		NEVER OR ALMOST NEVER	TIMES PER MONTH	TIMES PER WEEK			TIMES PER DAY			
			1-3	1	2-4	5-6	1	2-3	4-6	6+
	125.1									
	125.2									
	125.3									

Bland-Altman plots for all food groups and nutrients

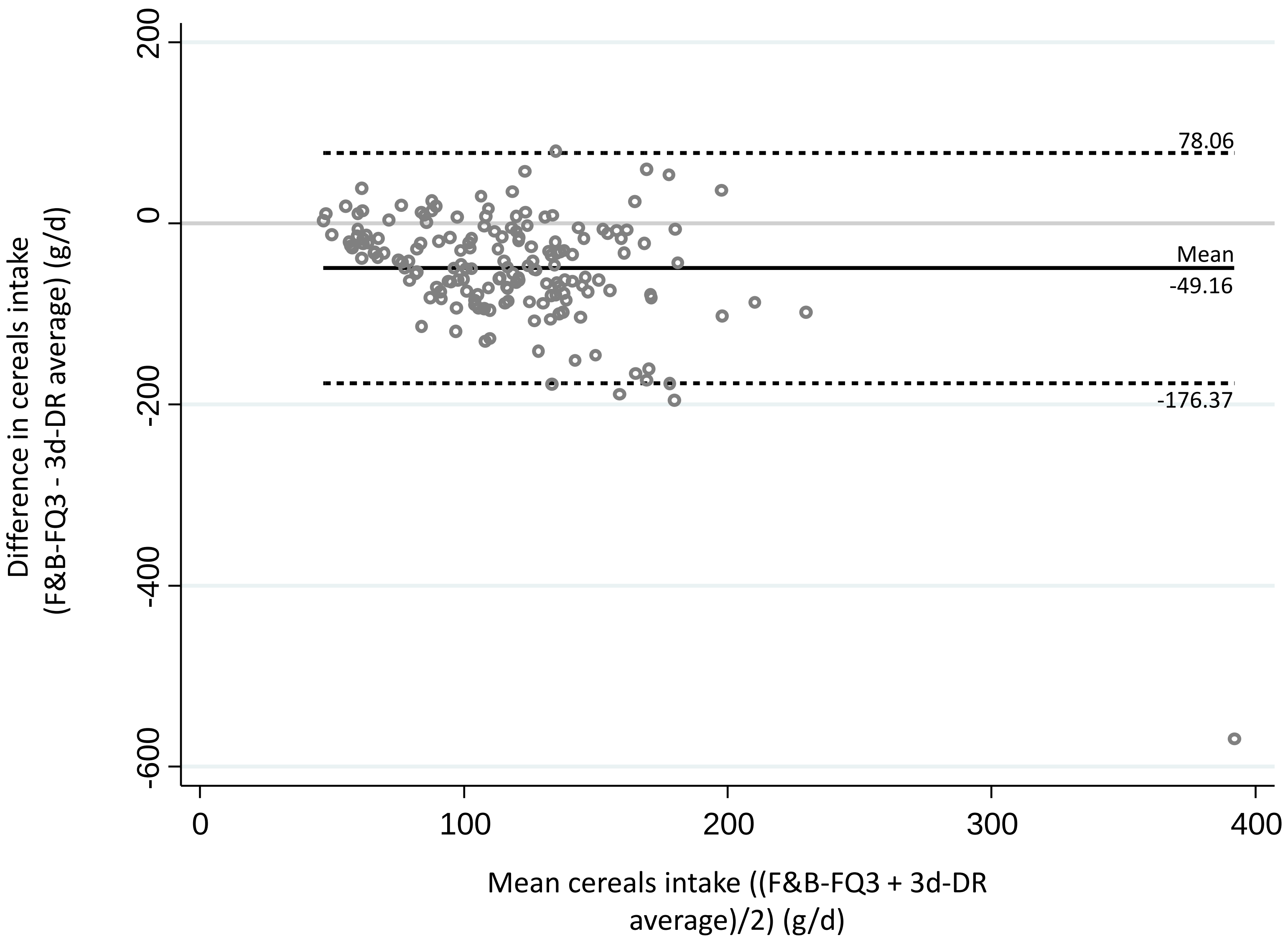

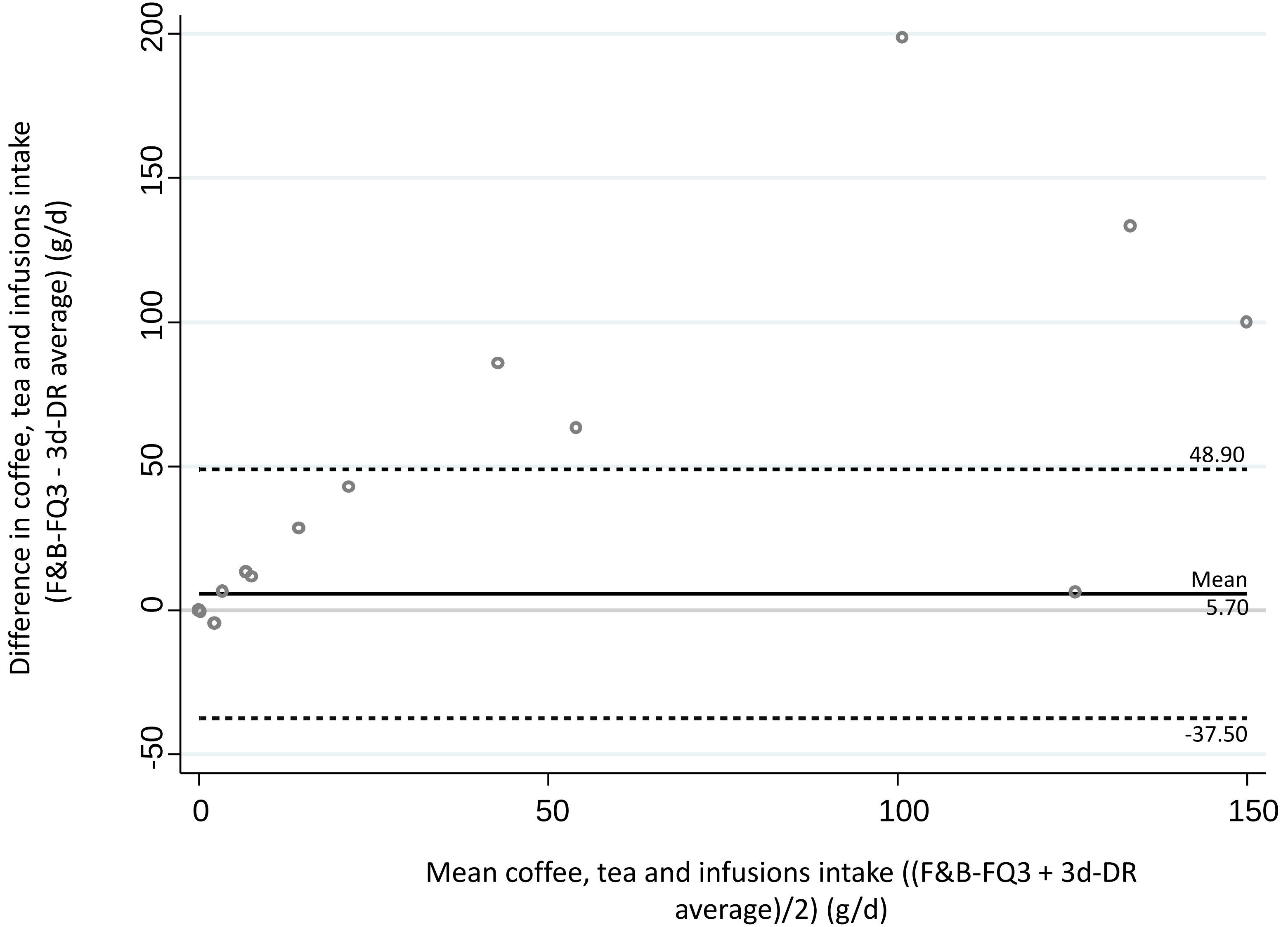

Difference in coffee, tea and infusions intake
(F&B-FQ3 - 3d-DR average) (g/d)
200
150
100
50
0
-50
48.90
Mean
5.70
-37.50
0
50
100
150
Mean coffee, tea and infusions intake ((F&B-FQ3 + 3d-DR average)/2) (g/d)

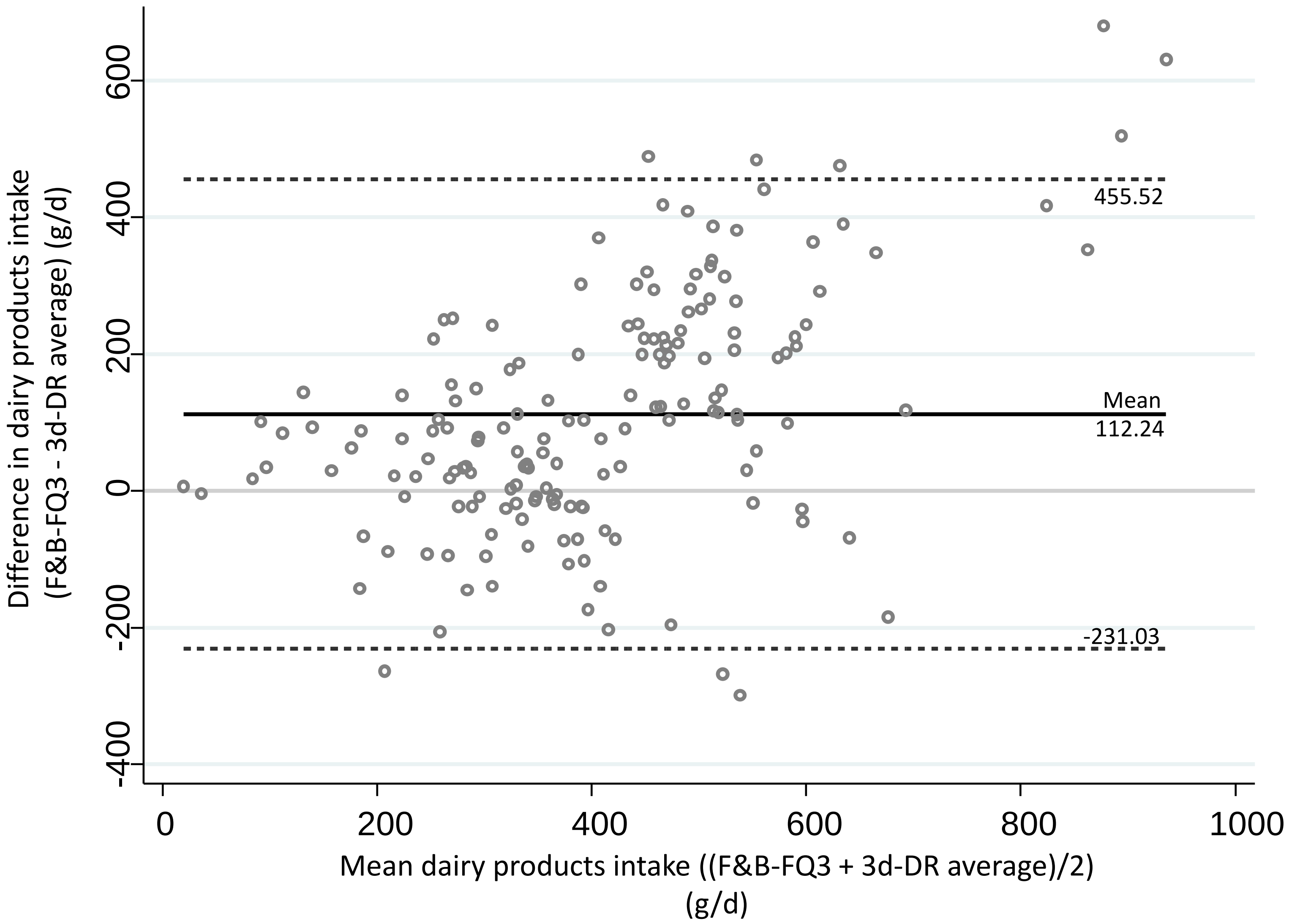

Difference in dairy products intake
(F&B-FQ3 - 3d-DR average) (g/d)
600
400
200
0
-200
-400
455.52
Mean
112.24
-231.03
0
200
400
600
800
1000
Mean dairy products intake ((F&B-FQ3 + 3d-DR average)/2)
(g/d)

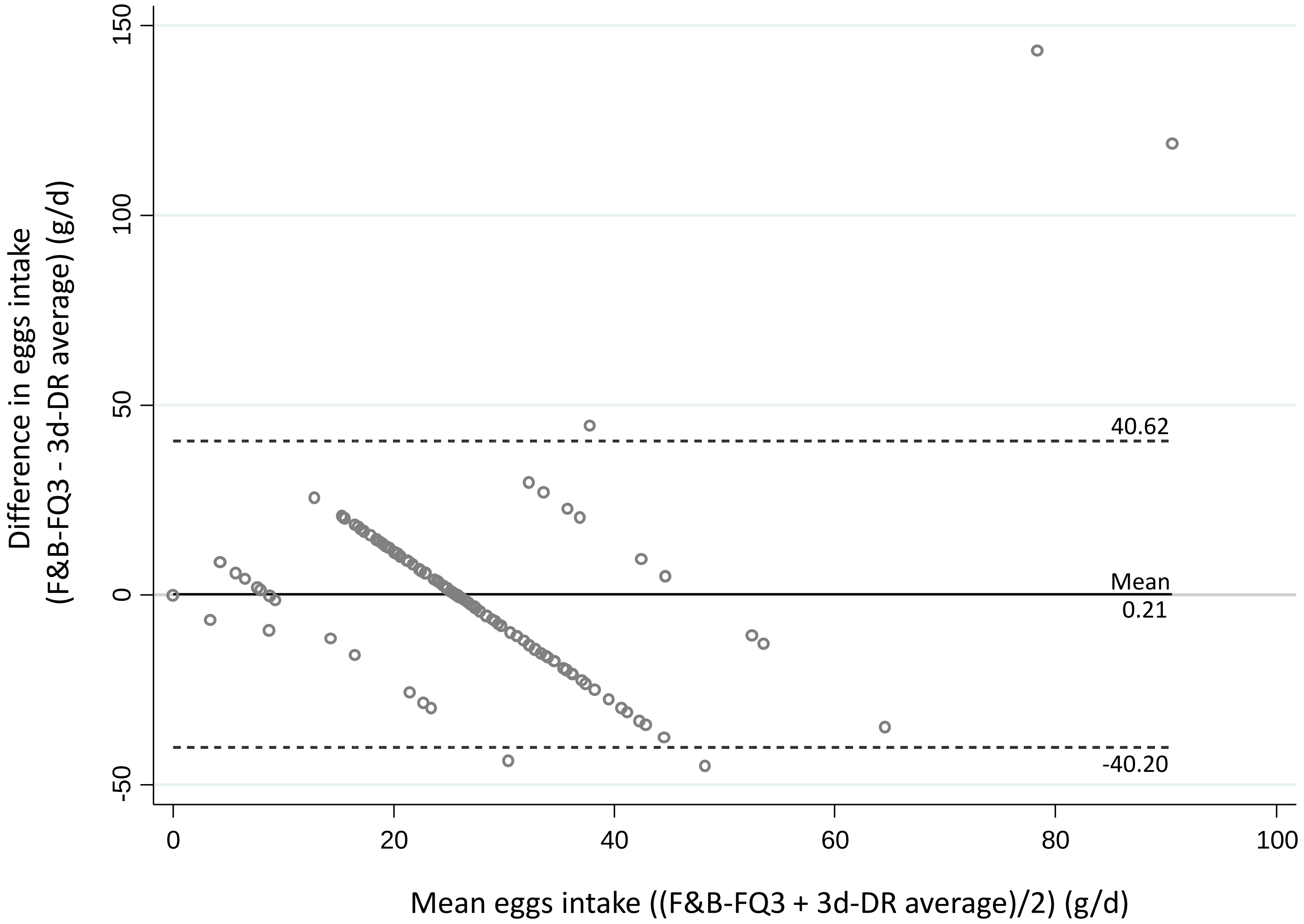

150
100
50
0
-50
Difference in eggs intake
(F&B-FQ3 - 3d-DR average) (g/d)
40.62
Mean
0.21
-40.20
0
20
40
60
80
100
Mean eggs intake ((F&B-FQ3 + 3d-DR average)/2) (g/d)

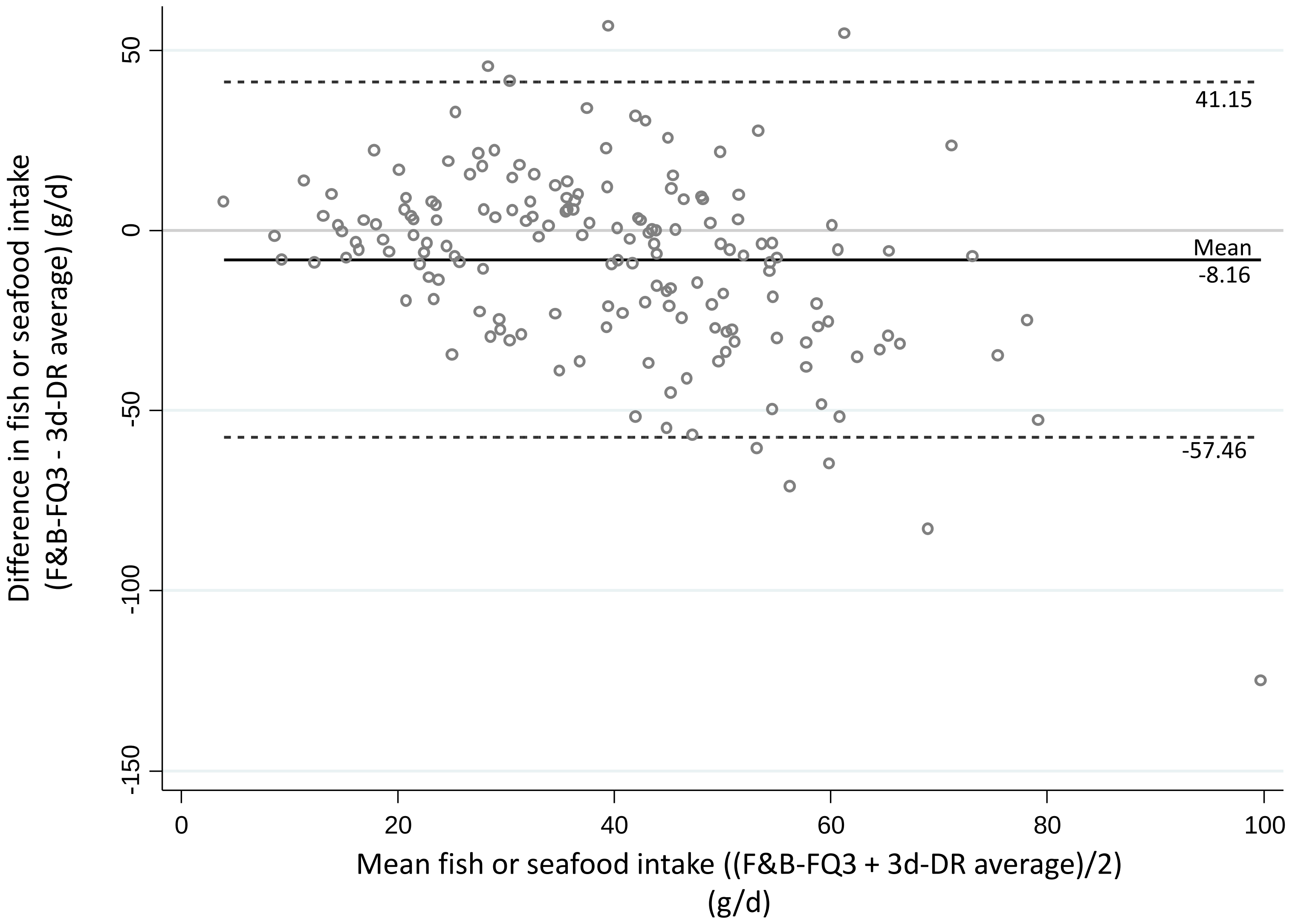

Difference in fish or seafood intake
(F&B-FQ3 - 3d-DR average) (g/d)
50
0
-50
-100
-150
41.15
Mean
-8.16
-57.46
0
20
40
60
80
100
Mean fish or seafood intake ((F&B-FQ3 + 3d-DR average)/2)
(g/d)

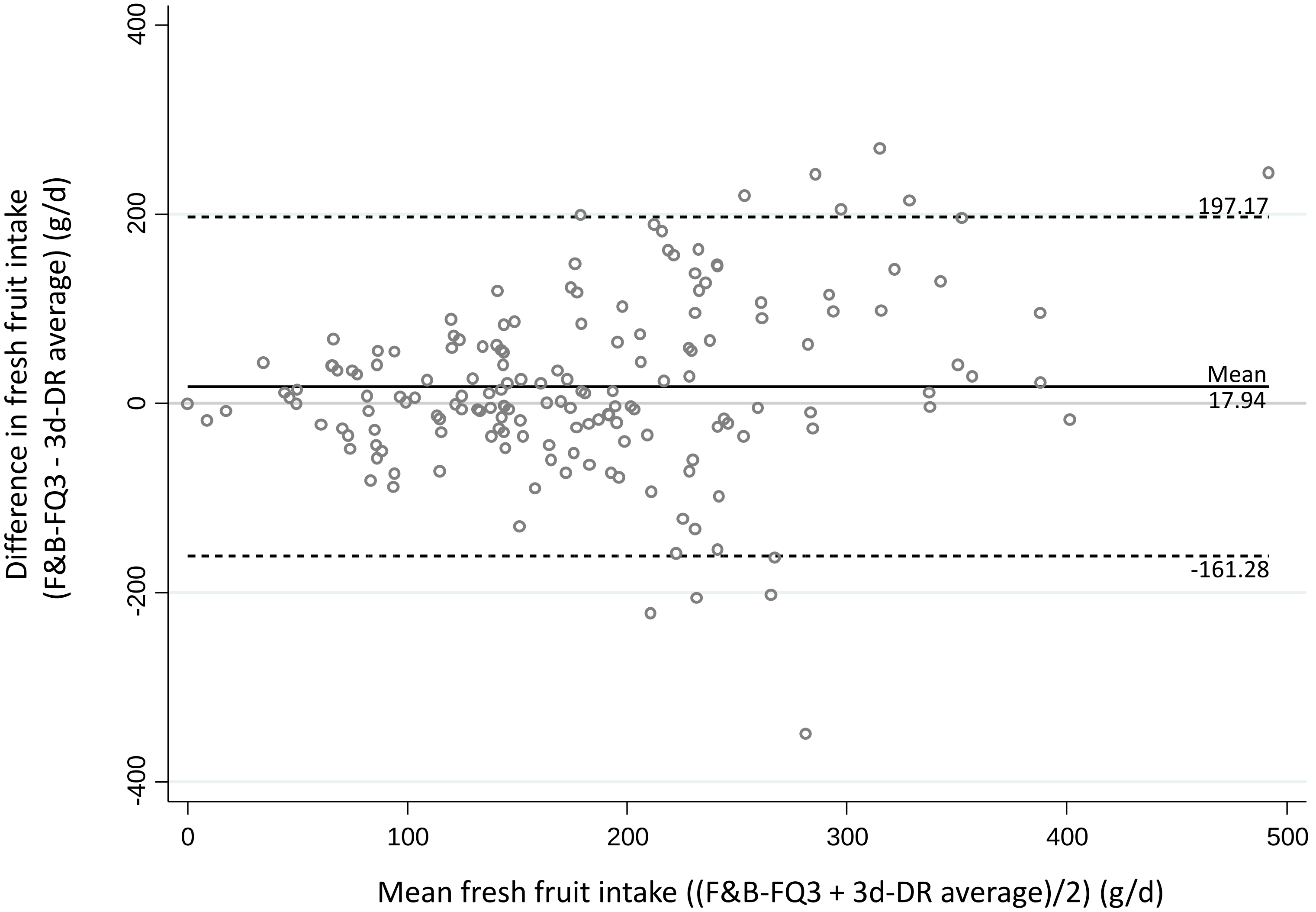

Difference in fresh fruit intake (F&B-FQ3 - 3d-DR average) (g/d)
400
200
0
-200
-400
197.17
Mean
17.94
-161.28
0
100
200
300
400
500
Mean fresh fruit intake ((F&B-FQ3 + 3d-DR average)/2) (g/d)

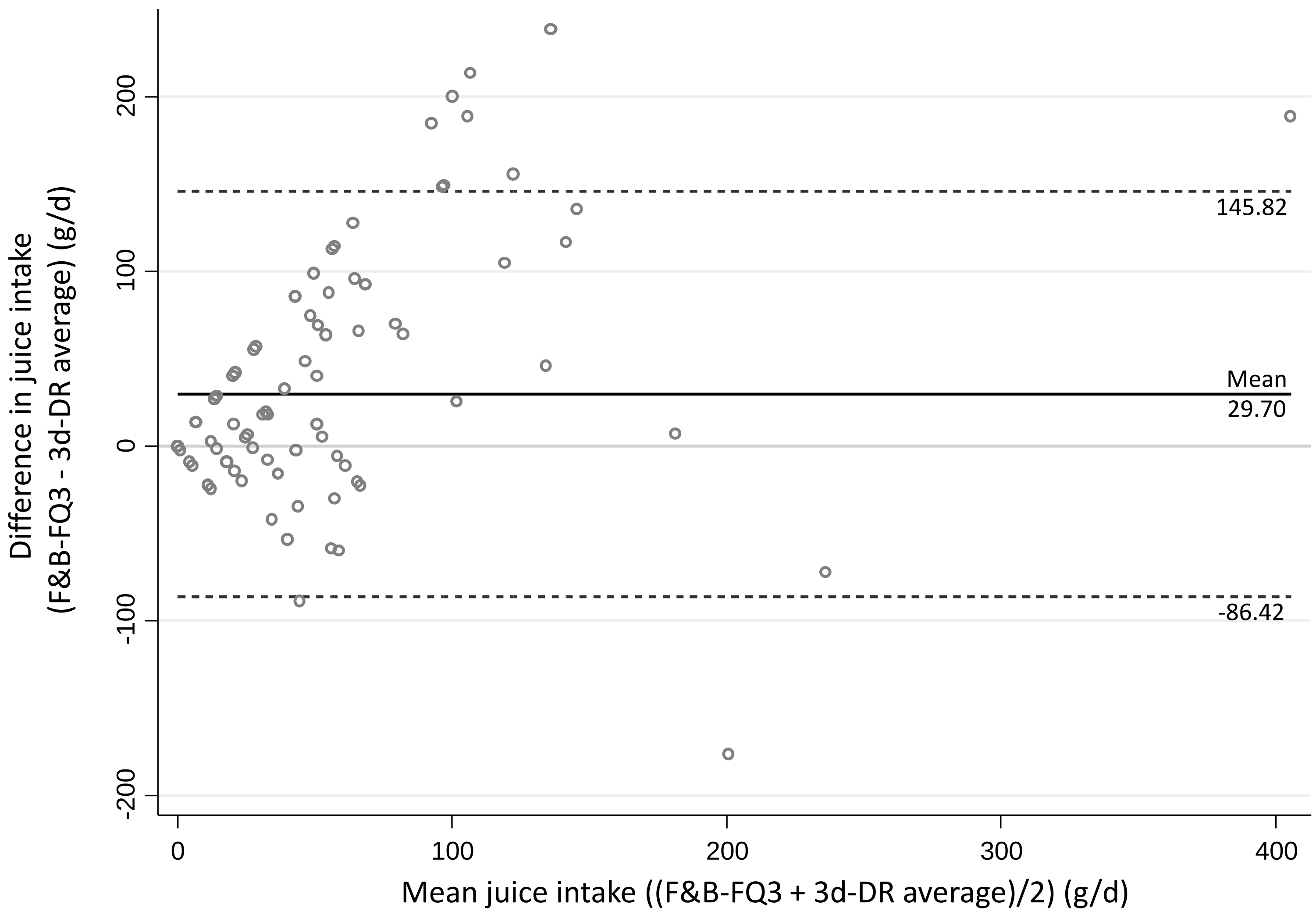
Difference in juice intake
(F&B-FQ3 - 3d-DR average) (g/d)
200
100
0
-100
-200
145.82
Mean
29.70
-86.42
0
100
200
300
400
Mean juice intake ((F&B-FQ3 + 3d-DR average)/2) (g/d)

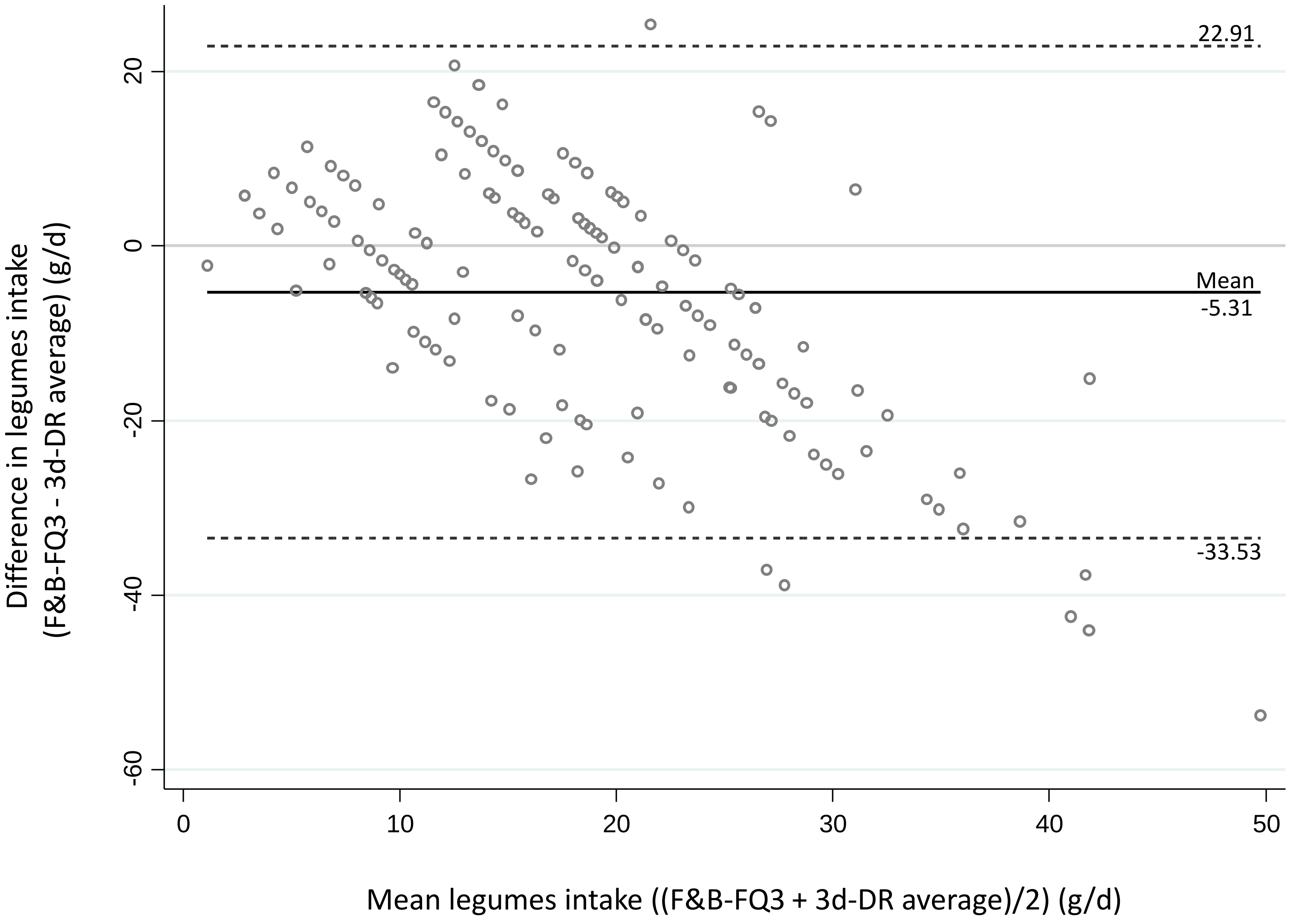

22.91
Mean
-5.31
-33.53
20
0
-20
-40
-60
0
10
20
30
40
50
Difference in legumes intake
(F&B-FQ3 - 3d-DR average) (g/d)
Mean legumes intake ((F&B-FQ3 + 3d-DR average)/2) (g/d)

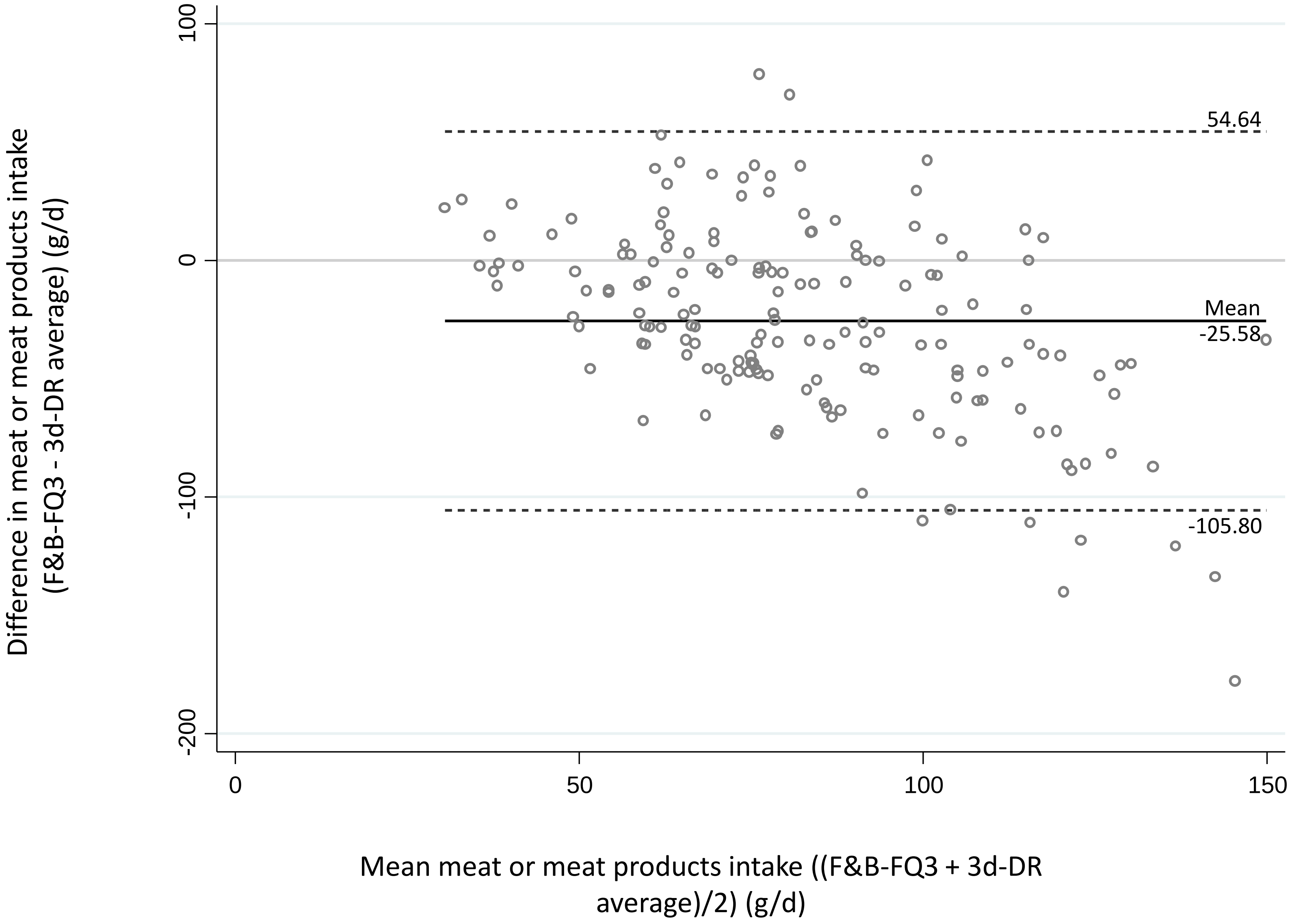

Difference in meat or meat products intake
(F&B-FQ3 - 3d-DR average) (g/d)
100
0
-100
-200
54.64
Mean
-25.58
-105.80
0
50
100
150
Mean meat or meat products intake ((F&B-FQ3 + 3d-DR
average)/2) (g/d)

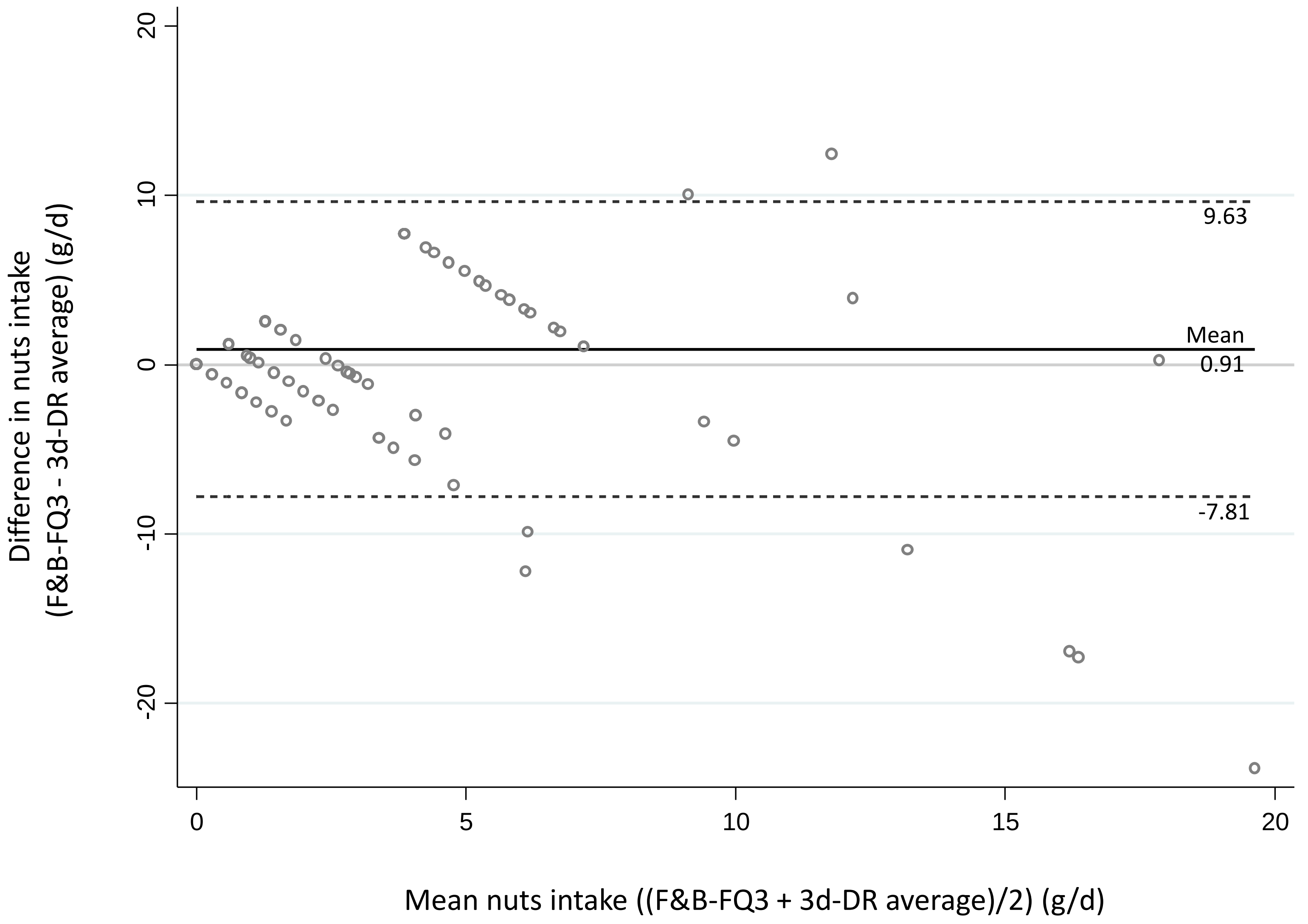

Difference in nuts intake
(F&B-FQ3 - 3d-DR average) (g/d)
20
10
0
-10
-20
9.63
Mean
0.91
-7.81
0
5
10
15
20
Mean nuts intake ((F&B-FQ3 + 3d-DR average)/2) (g/d)

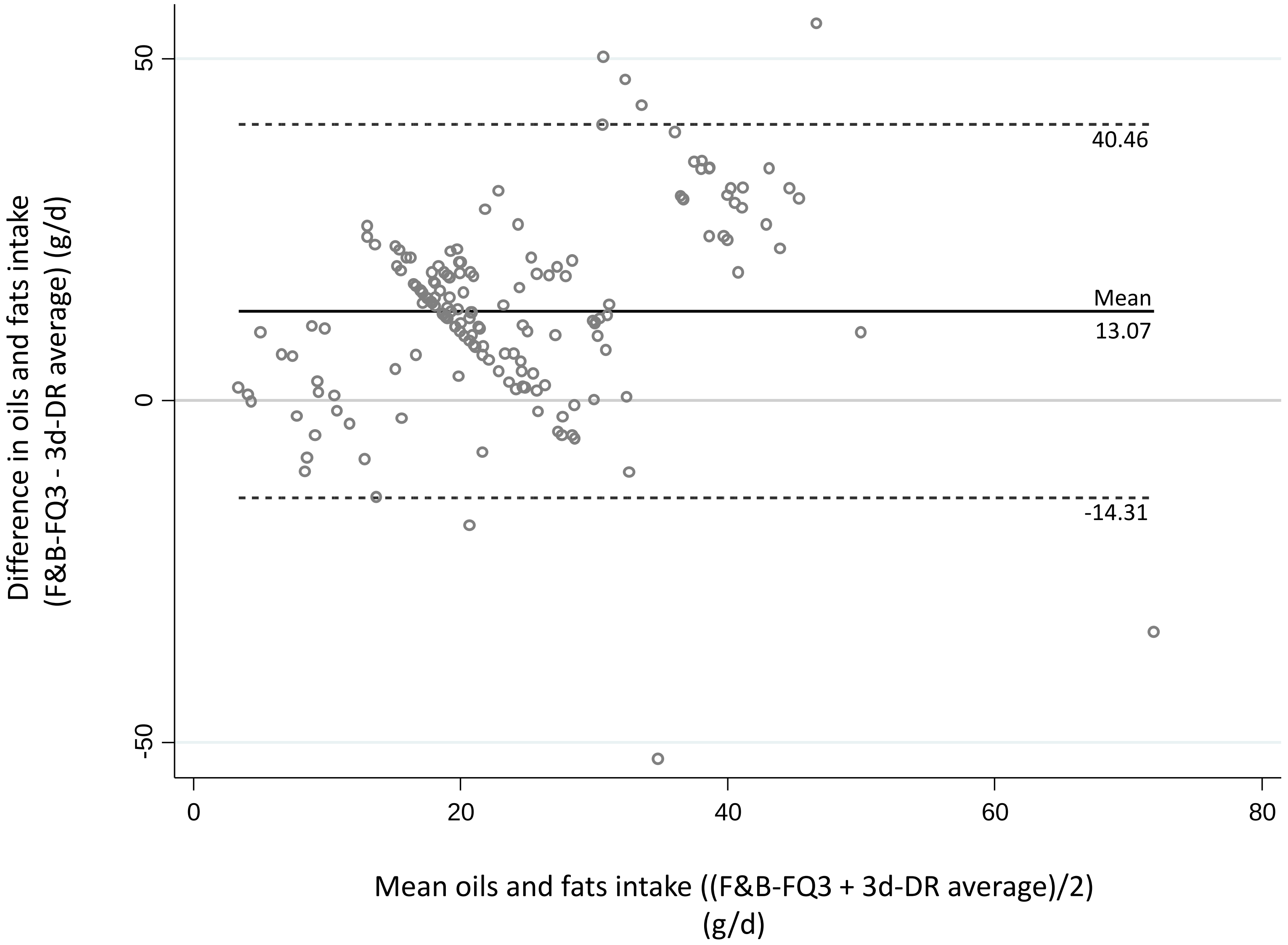

Difference in oils and fats intake
(F&B-FQ3 - 3d-DR average) (g/d)
50
0
-50
40.46
Mean
13.07
-14.31
0
20
40
60
80
Mean oils and fats intake ((F&B-FQ3 + 3d-DR average)/2)
(g/d)

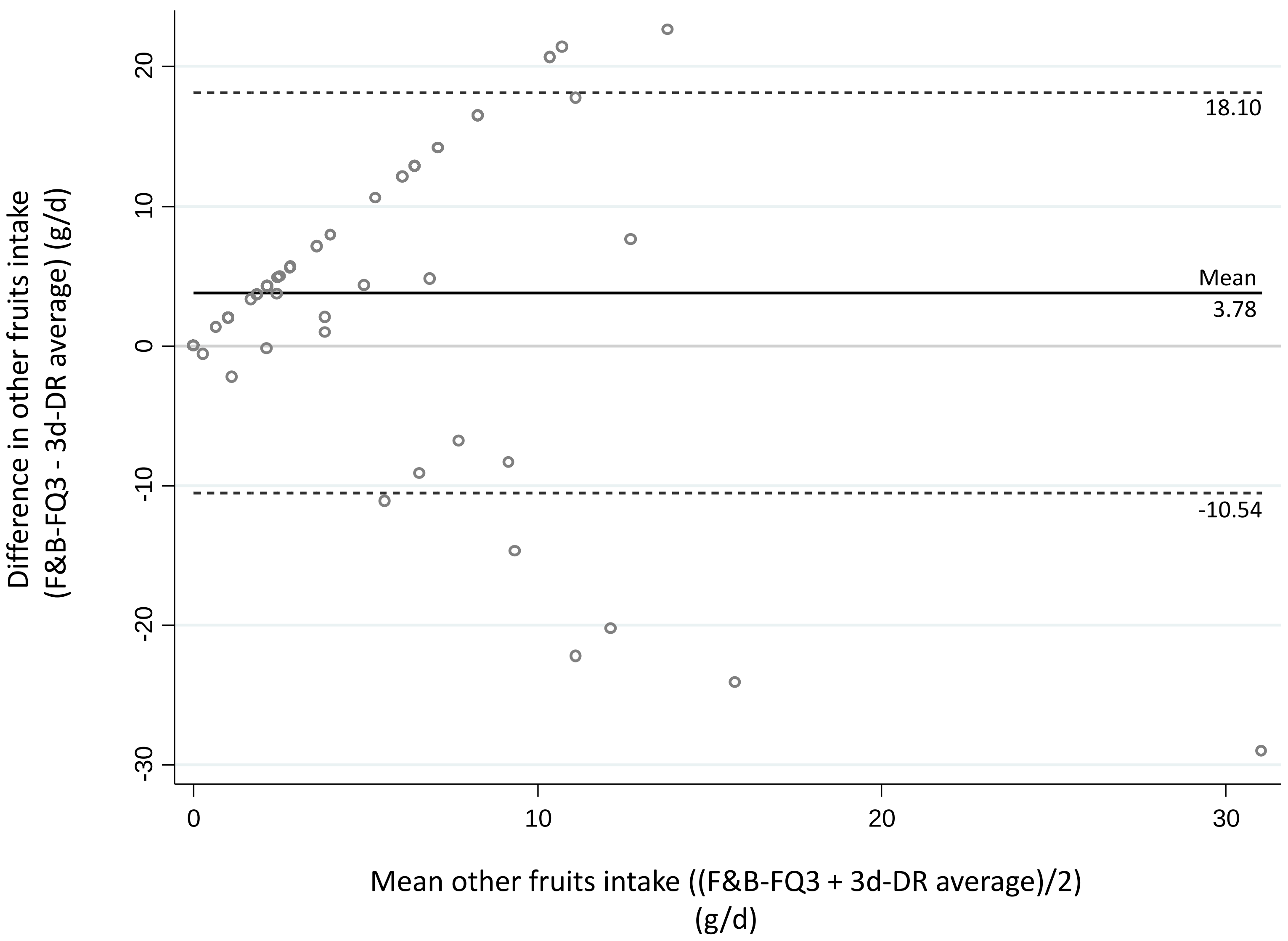
Difference in other fruits intake
(F&B-FQ3 - 3d-DR average) (g/d)
20
10
0
-10
-20
-30
18.10
Mean
3.78
-10.54
0
10
20
30
Mean other fruits intake ((F&B-FQ3 + 3d-DR average)/2)
(g/d)

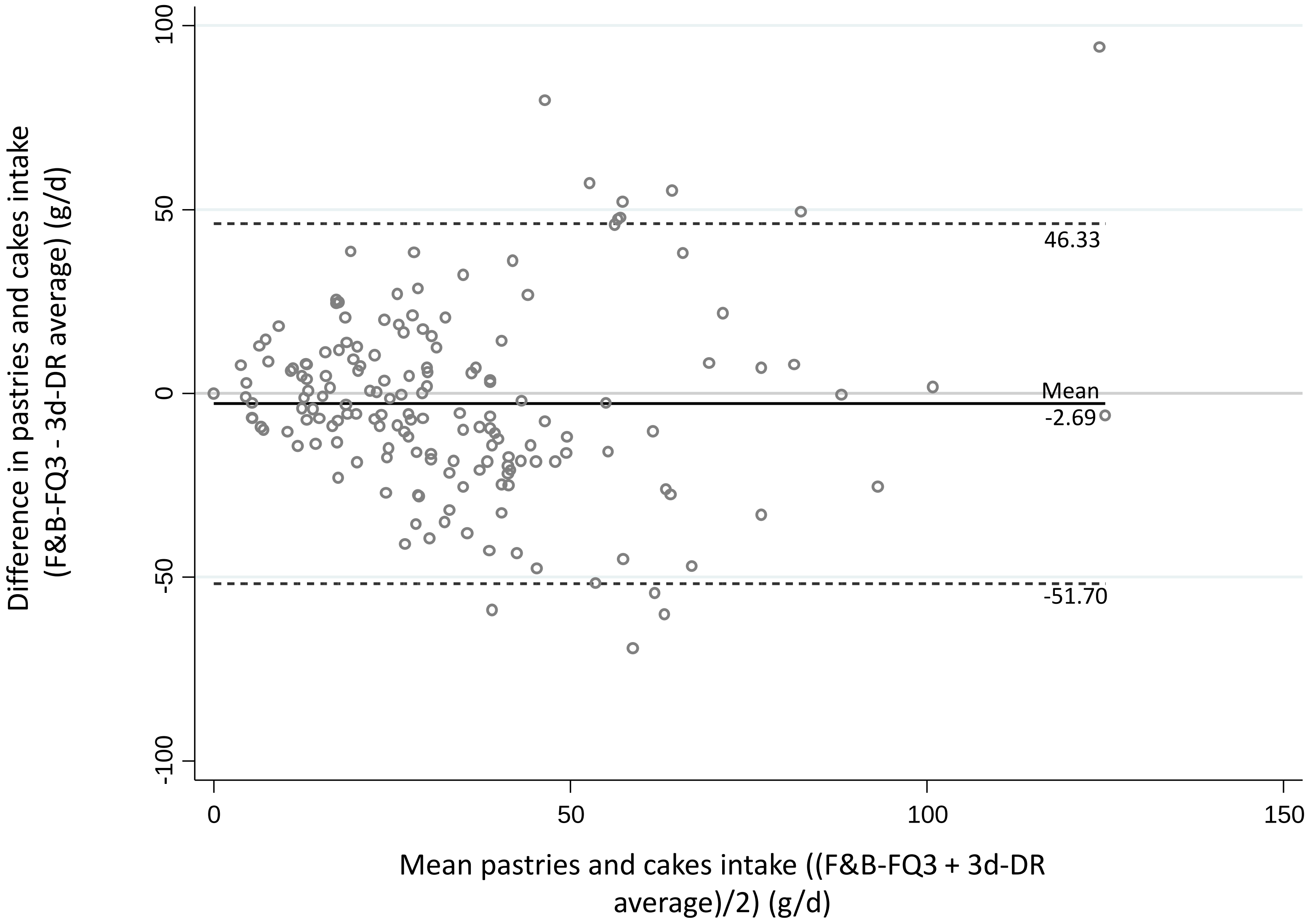

Difference in pastries and cakes intake
(F&B-FQ3 - 3d-DR average) (g/d)
100
50
0
-50
-100
46.33
Mean
-2.69
-51.70
0
50
100
150
Mean pastries and cakes intake ((F&B-FQ3 + 3d-DR
average)/2) (g/d)

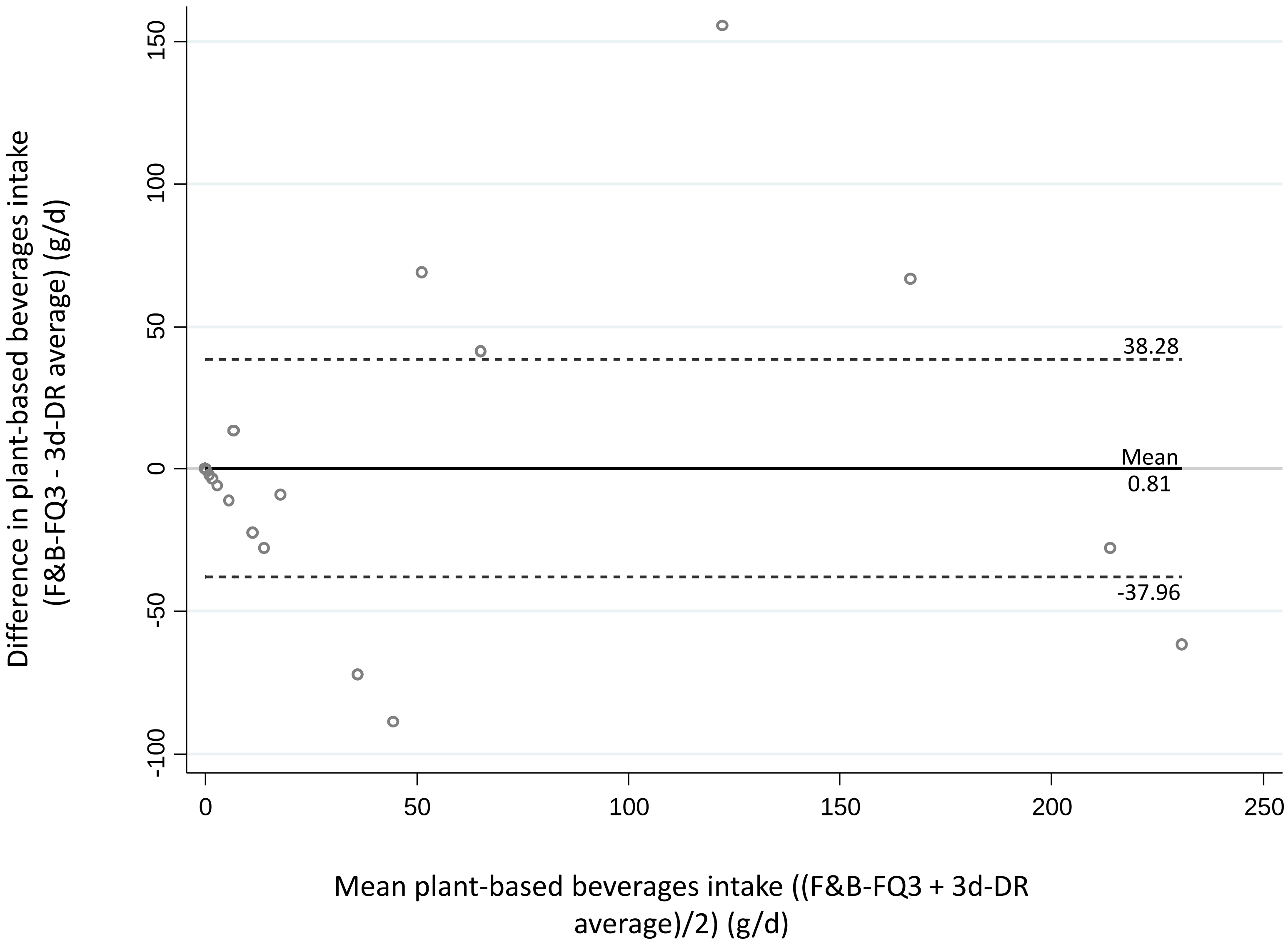

Difference in plant-based beverages intake (F&B-FQ3 - 3d-DR average) (g/d)
150
100
50
0
-50
-100
38.28
Mean
0.81
-37.96
0
50
100
150
200
250
Mean plant-based beverages intake ((F&B-FQ3 + 3d-DR average)/2) (g/d)

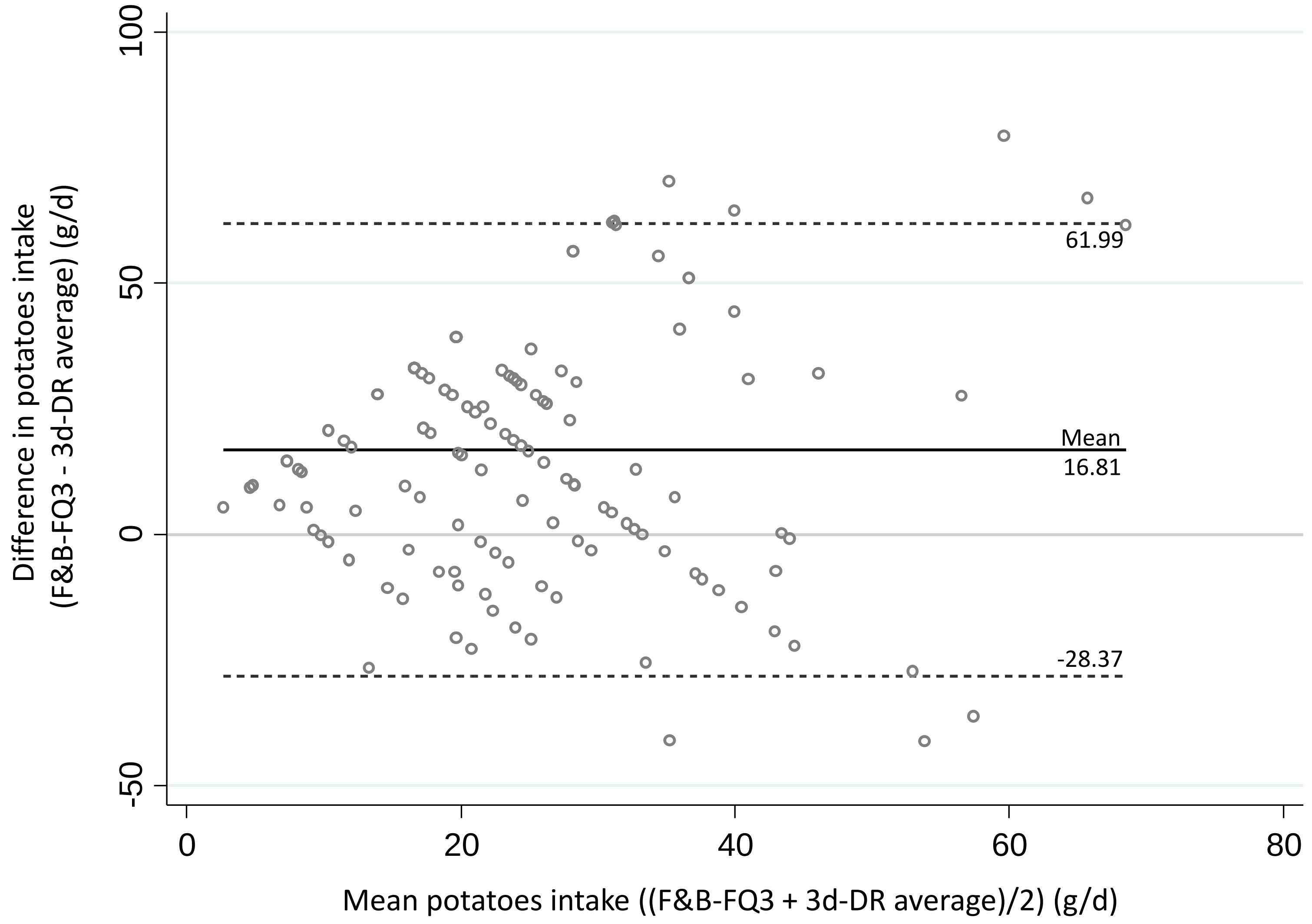

Difference in potatoes intake
(F&B-FQ3 - 3d-DR average) (g/d)
100
50
0
-50
61.99
Mean
16.81
-28.37
0
20
40
60
80
Mean potatoes intake ((F&B-FQ3 + 3d-DR average)/2) (g/d)

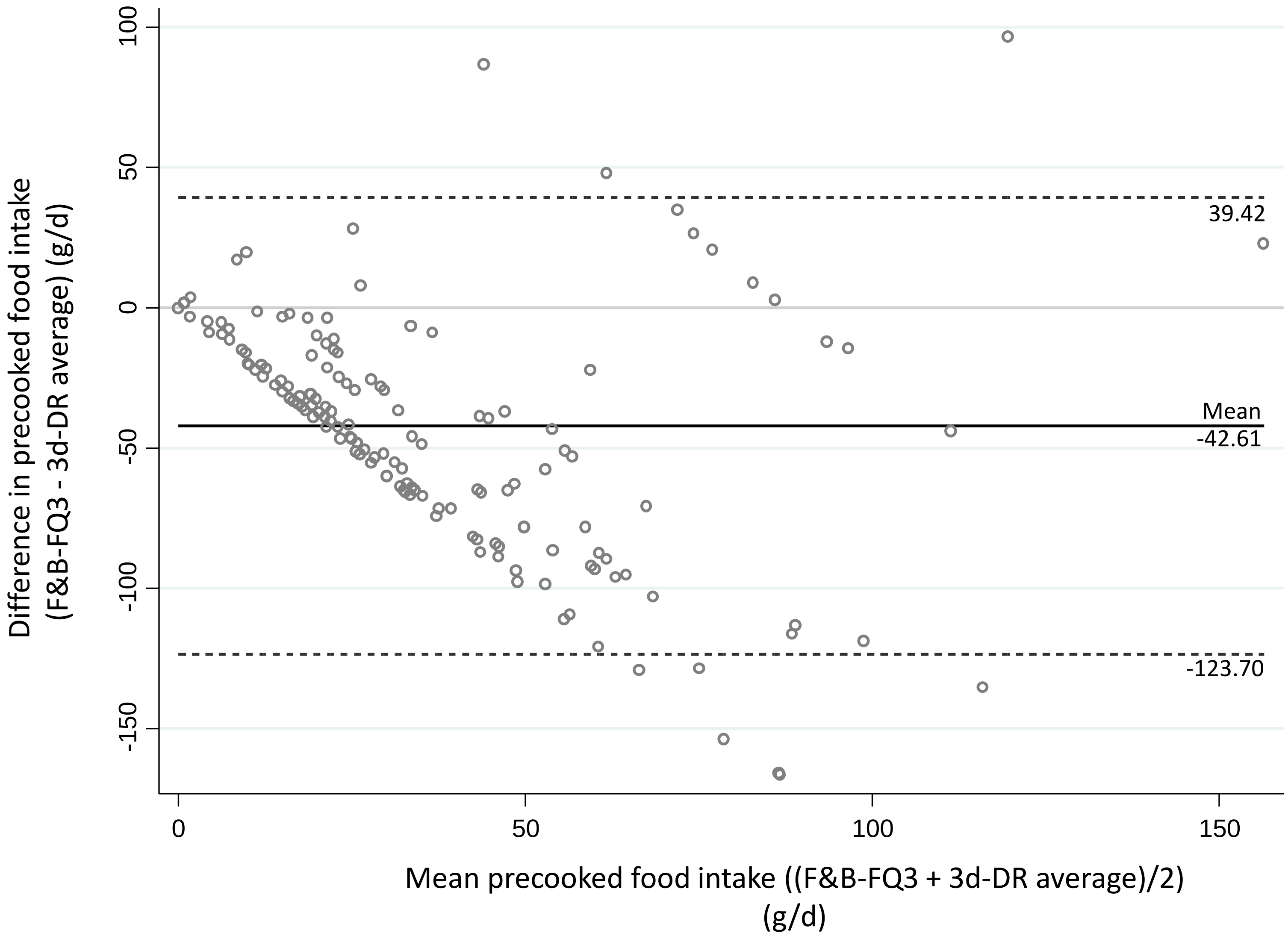

Difference in precooked food intake
(F&B-FQ3 - 3d-DR average) (g/d)
100
50
0
-50
-100
-150
39.42
Mean
-42.61
-123.70
0
50
100
150
Mean precooked food intake ((F&B-FQ3 + 3d-DR average)/2)
(g/d)

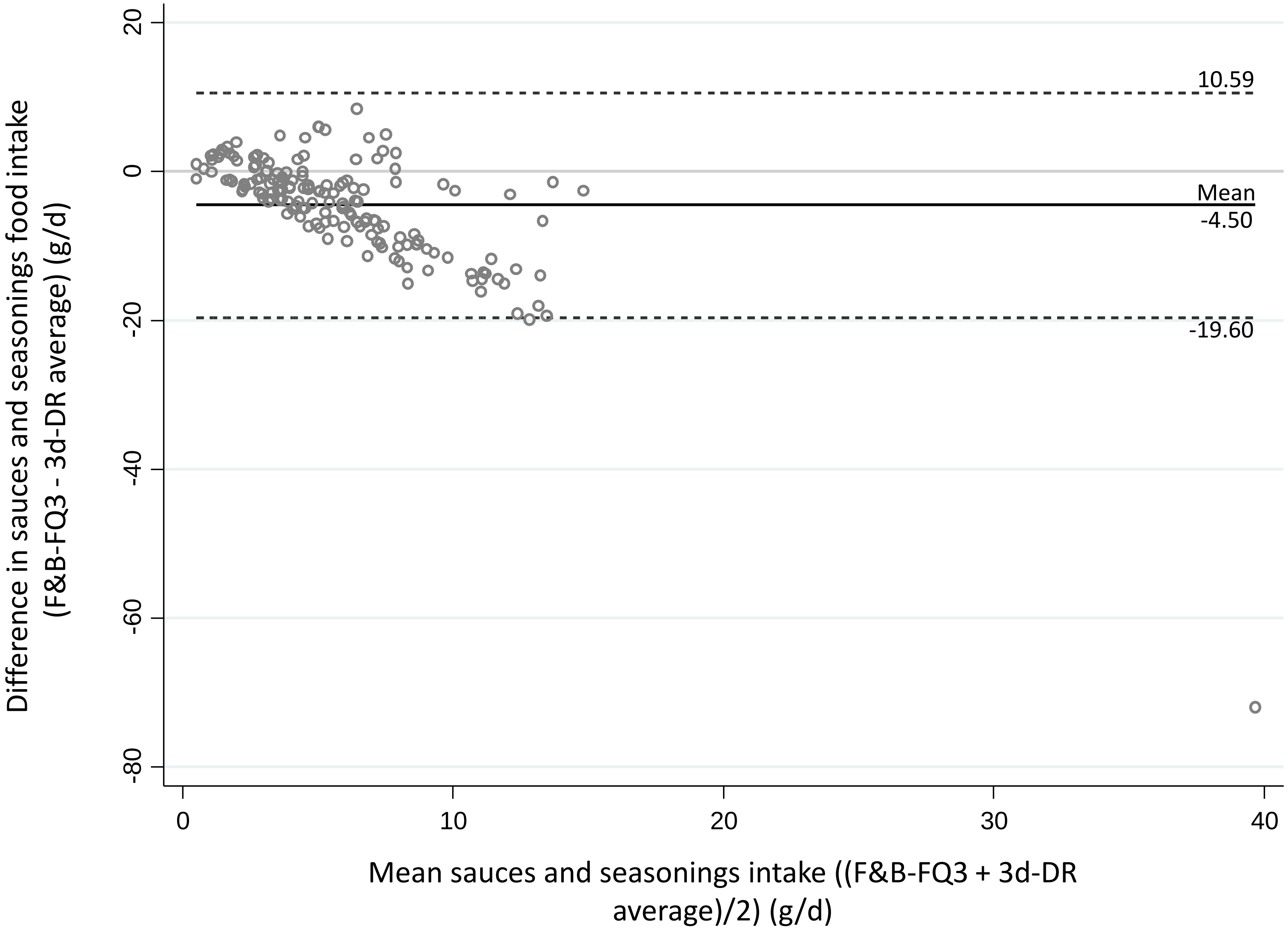

10.59
Mean
-4.50
-19.60
Difference in sauces and seasonings food intake (F&B-FQ3 - 3d-DR average) (g/d)
Mean sauces and seasonings intake ((F&B-FQ3 + 3d-DR average)/2) (g/d)
20
0
-20
-40
-60
-80
0
10
20
30
40

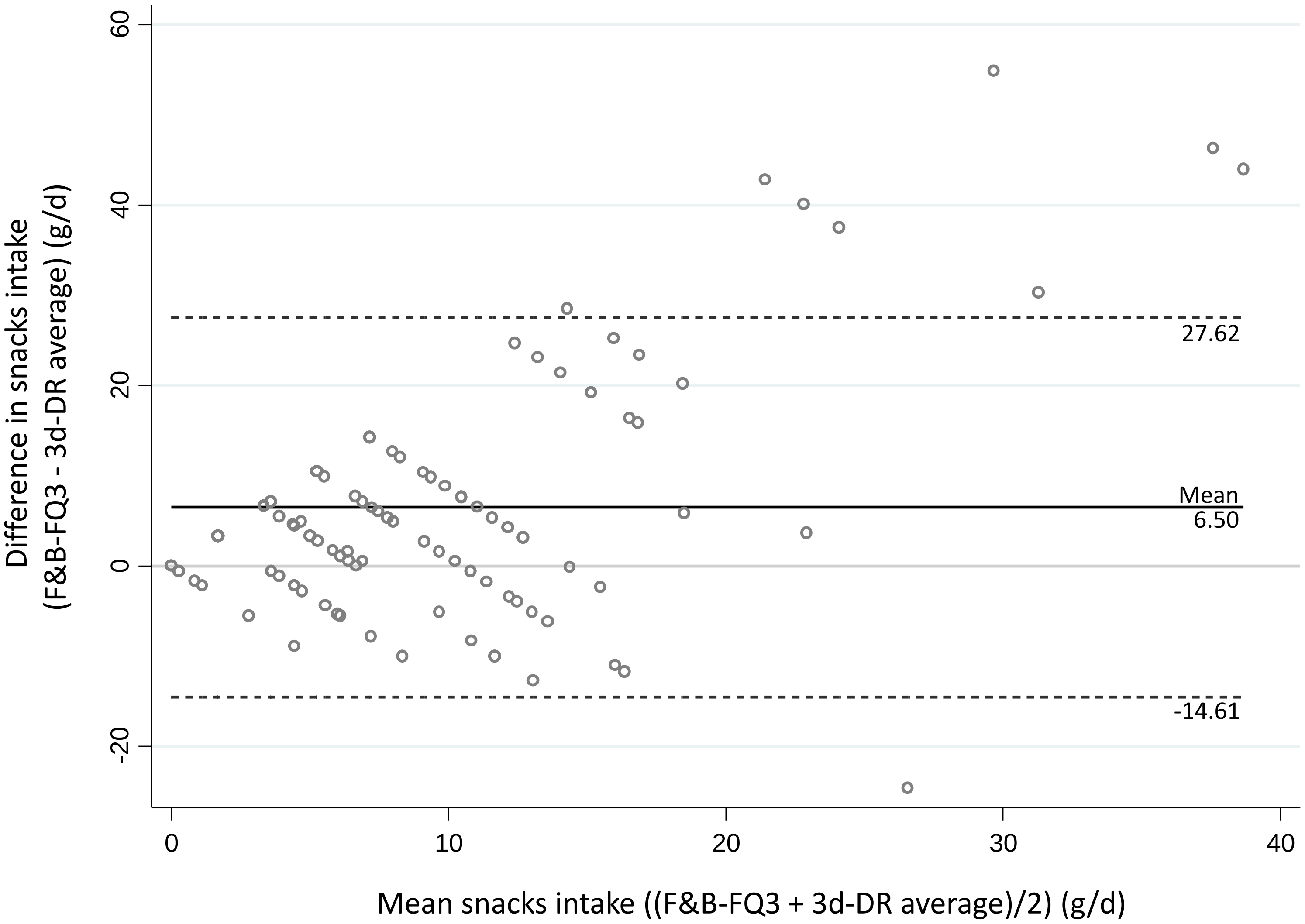

Difference in snacks intake
(F&B-FQ3 - 3d-DR average) (g/d)
60
40
20
0
-20
27.62
Mean
6.50
-14.61
0
10
20
30
40
Mean snacks intake ((F&B-FQ3 + 3d-DR average)/2) (g/d)

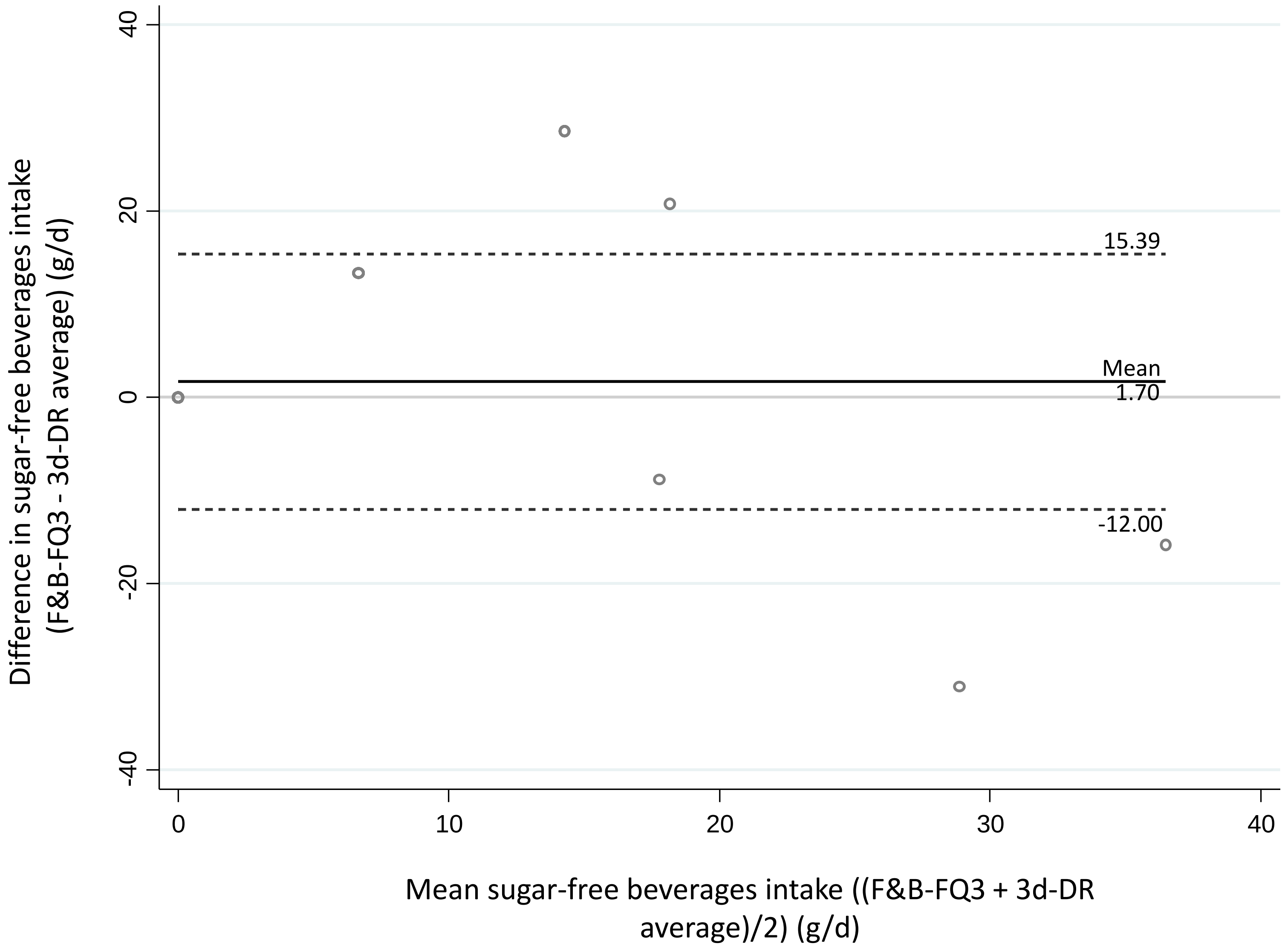

Difference in sugar-free beverages intake (F&B-FQ3 - 3d-DR average) (g/d)
Mean sugar-free beverages intake ((F&B-FQ3 + 3d-DR average)/2) (g/d)
15.39
Mean
1.70
-12.00
40
20
0
-20
-40
0
10
20
30
40

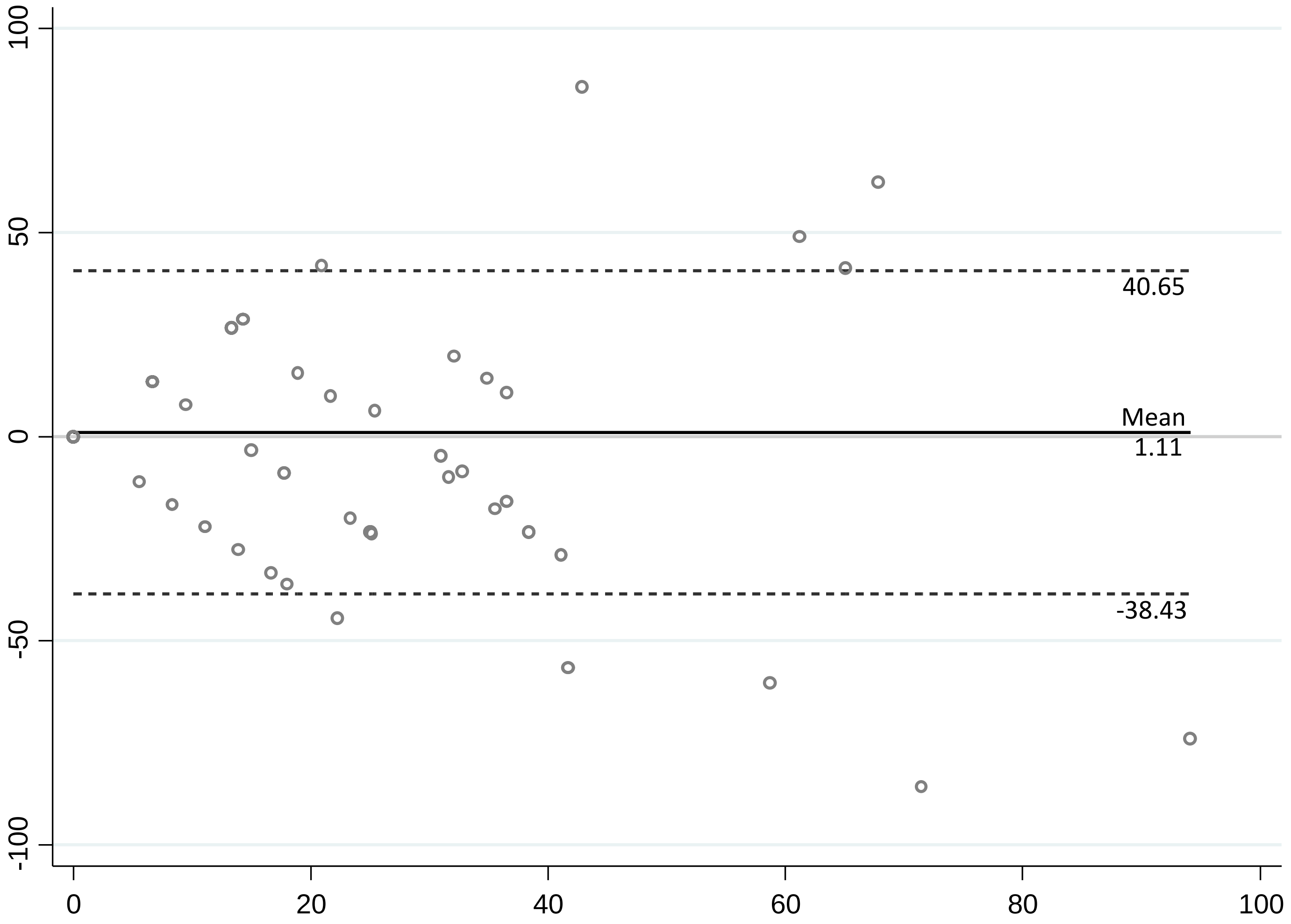

Difference in sweetened beverages intake (F&B-FQ3 - 3d-DR average) (g/d)

Mean sweetened beverages intake ((F&B-FQ3 + 3d-DR average)/2) (g/d)

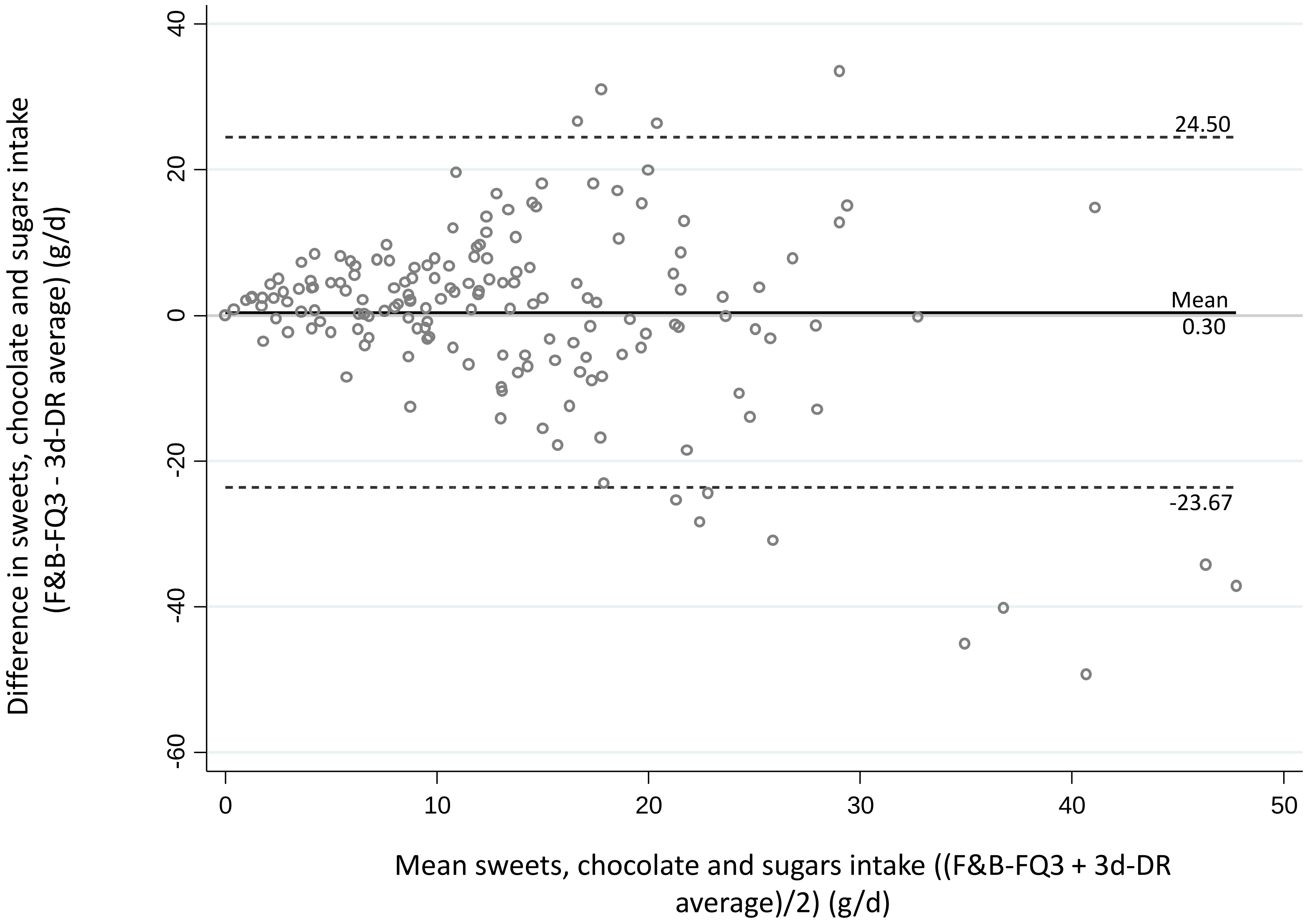

Difference in sweets, chocolate and sugars intake (F&B-FQ3 - 3d-DR average) (g/d)
40
20
0
-20
-40
-60
24.50
Mean
0.30
-23.67
0
10
20
30
40
50
Mean sweets, chocolate and sugars intake ((F&B-FQ3 + 3d-DR average)/2) (g/d)

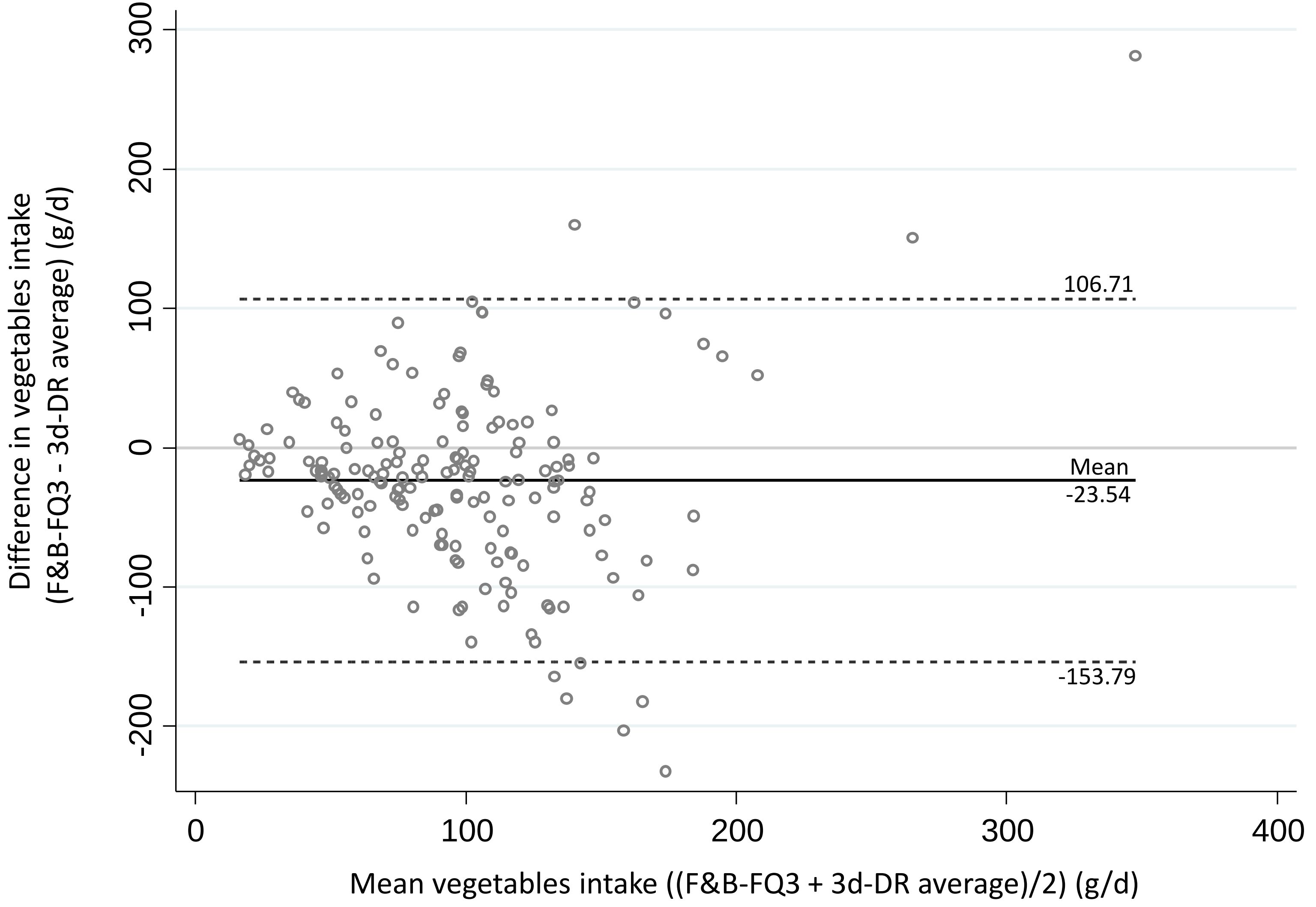

Difference in vegetables intake
(F&B-FQ3 - 3d-DR average) (g/d)
300
200
100
0
-100
-200
106.71
Mean
-23.54
-153.79
0
100
200
300
400
Mean vegetables intake ((F&B-FQ3 + 3d-DR average)/2) (g/d)

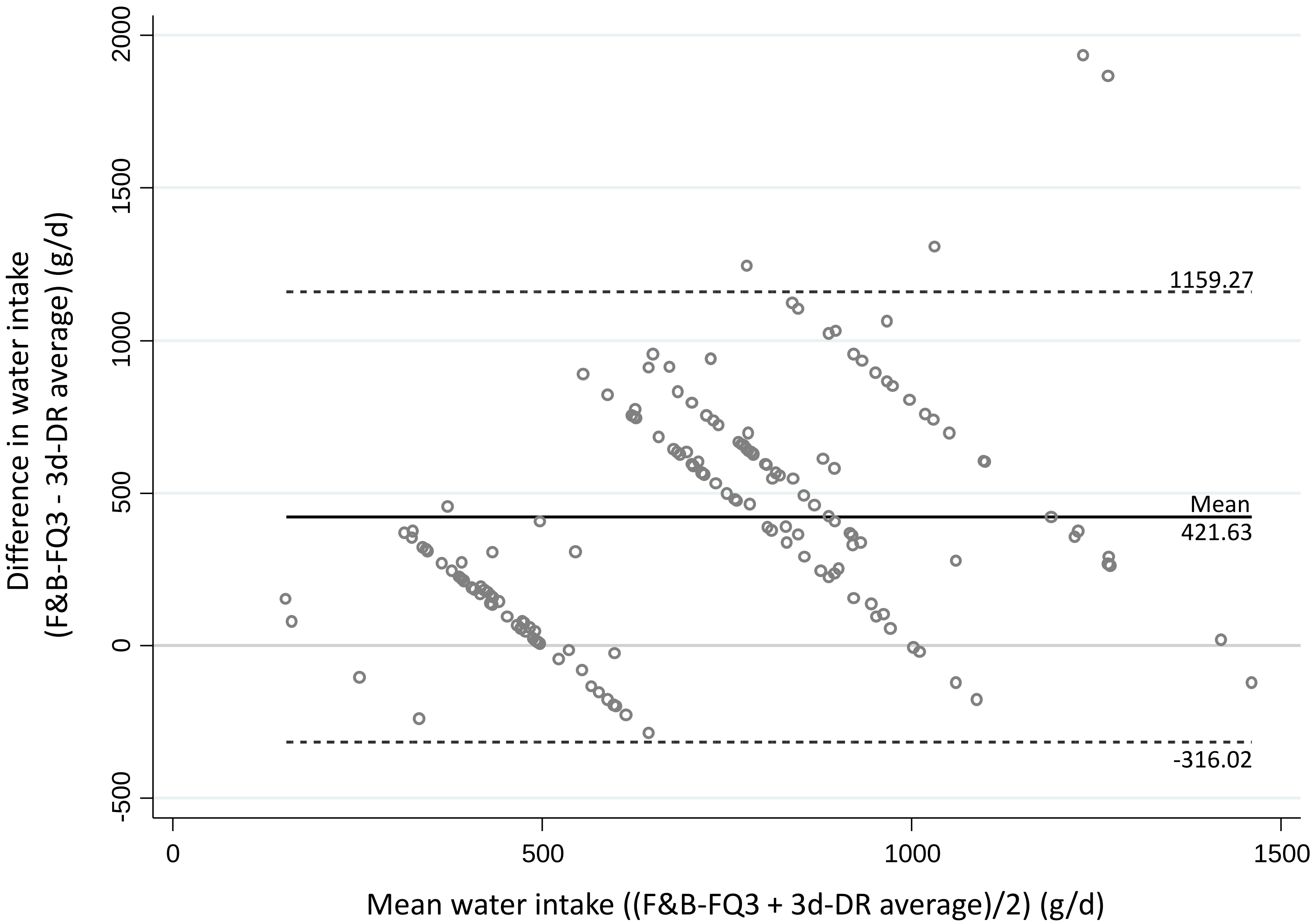

Difference in water intake
(F&B-FQ3 - 3d-DR average) (g/d)
2000
1500
1000
500
0
-500
1159.27
Mean
421.63
-316.02
0
500
1000
1500
Mean water intake ((F&B-FQ3 + 3d-DR average)/2) (g/d)

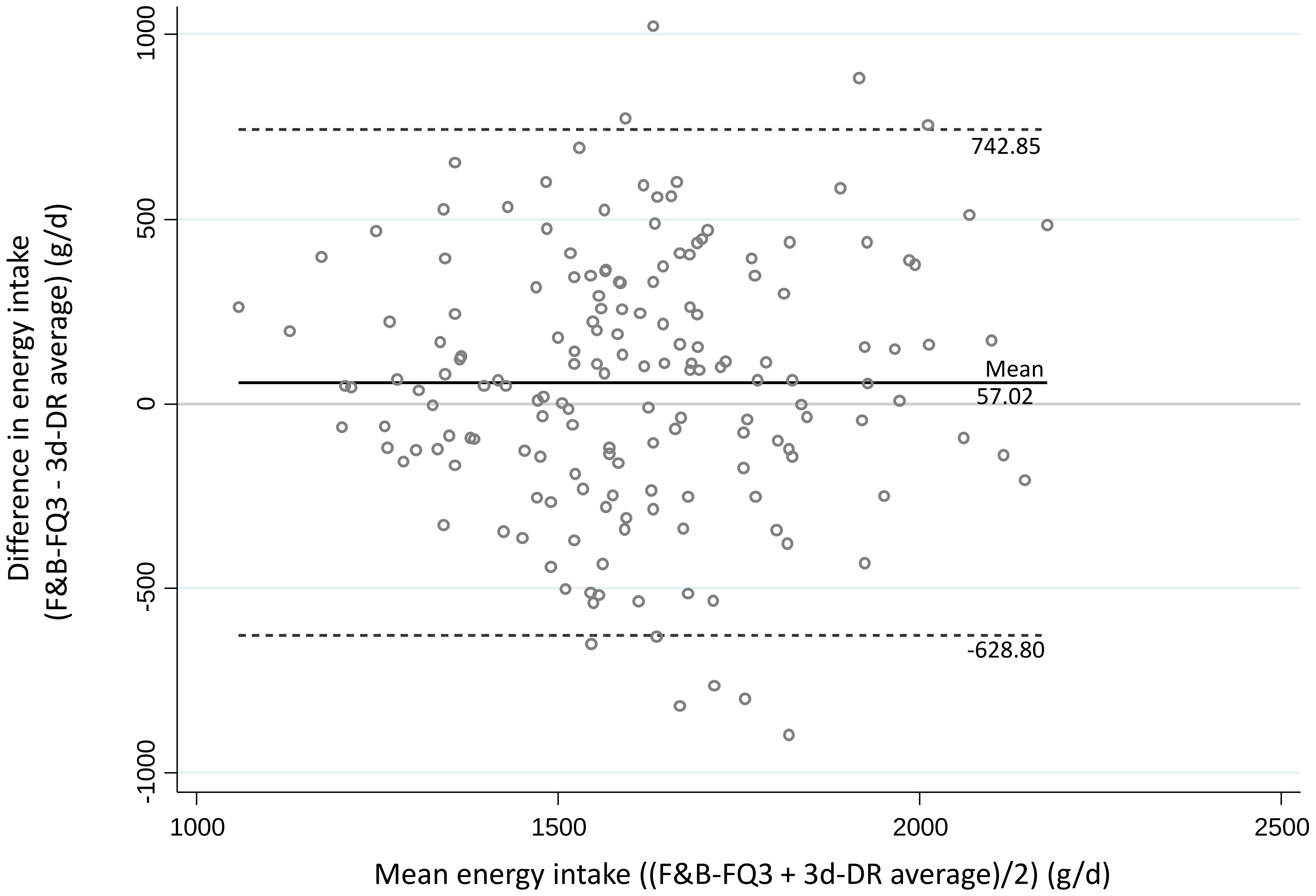

Difference in energy intake
(F&B-FQ3 - 3d-DR average) (g/d)
1000
500
0
-500
-1000
742.85
Mean
57.02
-628.80
1000
1500
2000
2500
Mean energy intake ((F&B-FQ3 + 3d-DR average)/2) (g/d)

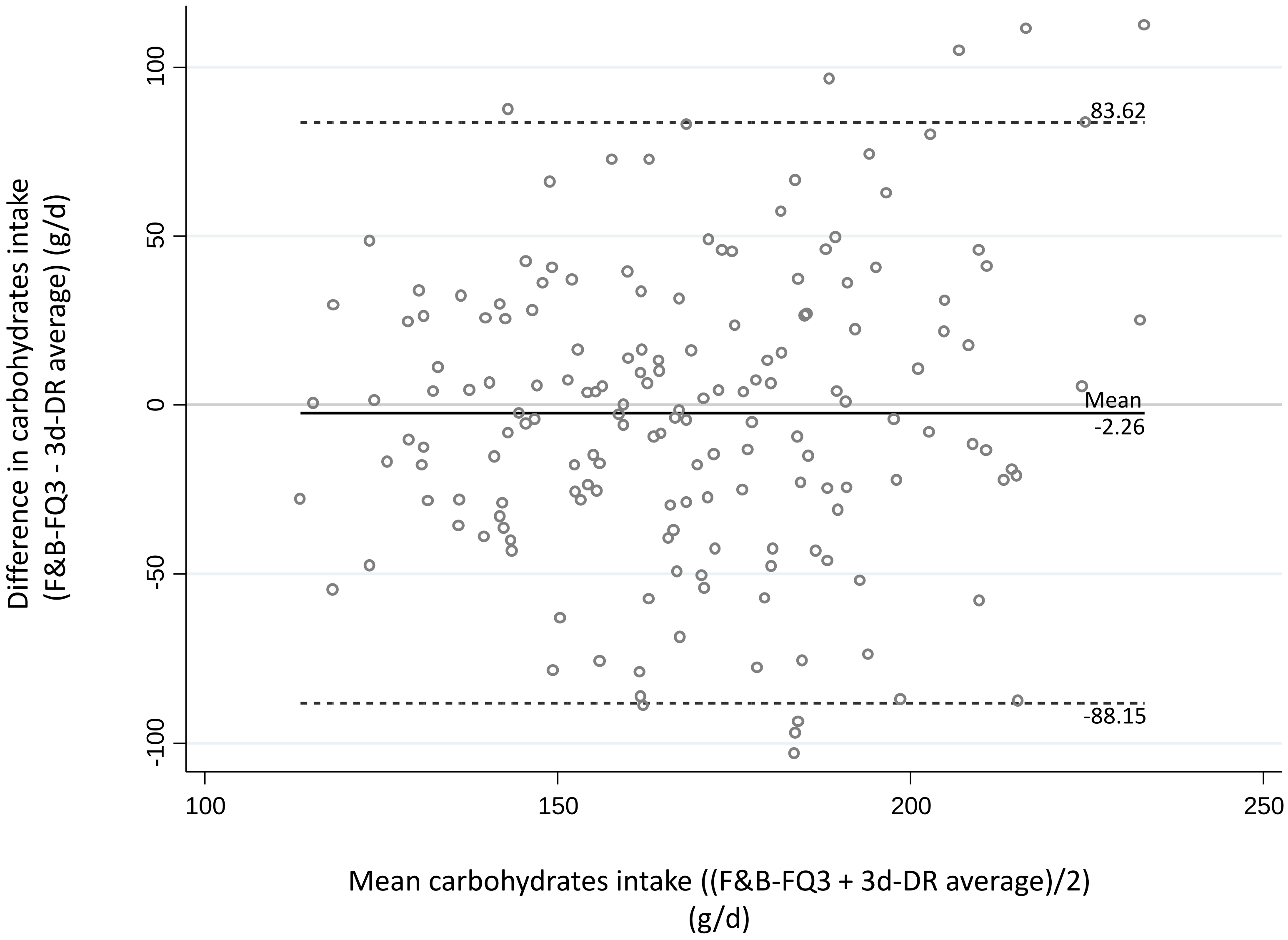

Difference in carbohydrates intake (F&B-FQ3 - 3d-DR average) (g/d)
100
50
0
-50
-100
83.62
Mean
-2.26
-88.15
100
150
200
250
Mean carbohydrates intake ((F&B-FQ3 + 3d-DR average)/2) (g/d)

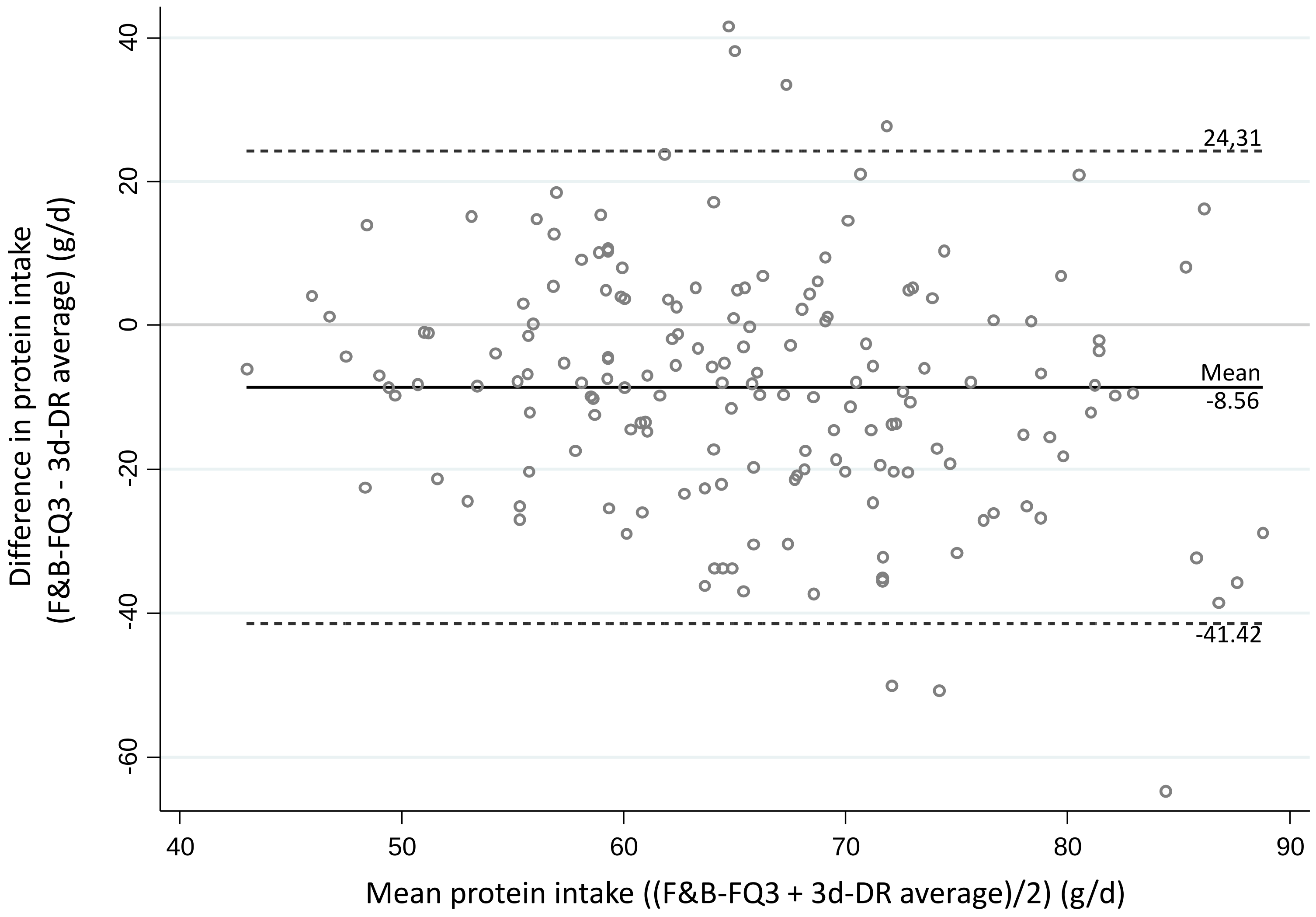

40
20
0
-20
-40
-60
Difference in protein intake
(F&B-FQ3 - 3d-DR average) (g/d)
24,31
Mean
-8.56
-41.42
40
50
60
70
80
90
Mean protein intake ((F&B-FQ3 + 3d-DR average)/2) (g/d)

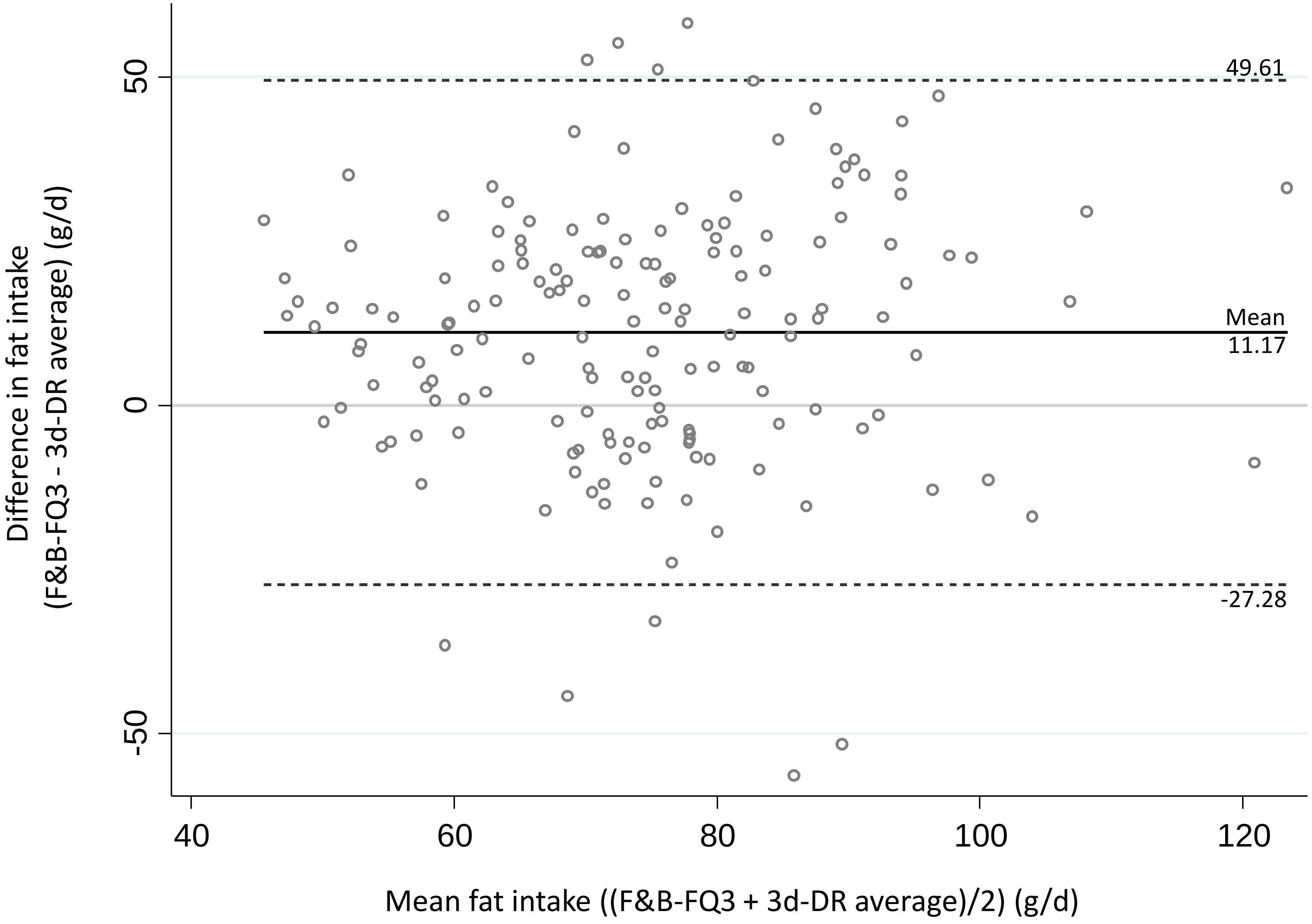

49.61
Mean
11.17
-27.28
50
0
-50
40
60
80
100
120
Difference in fat intake
(F&B-FQ3 - 3d-DR average) (g/d)
Mean fat intake ((F&B-FQ3 + 3d-DR average)/2) (g/d)

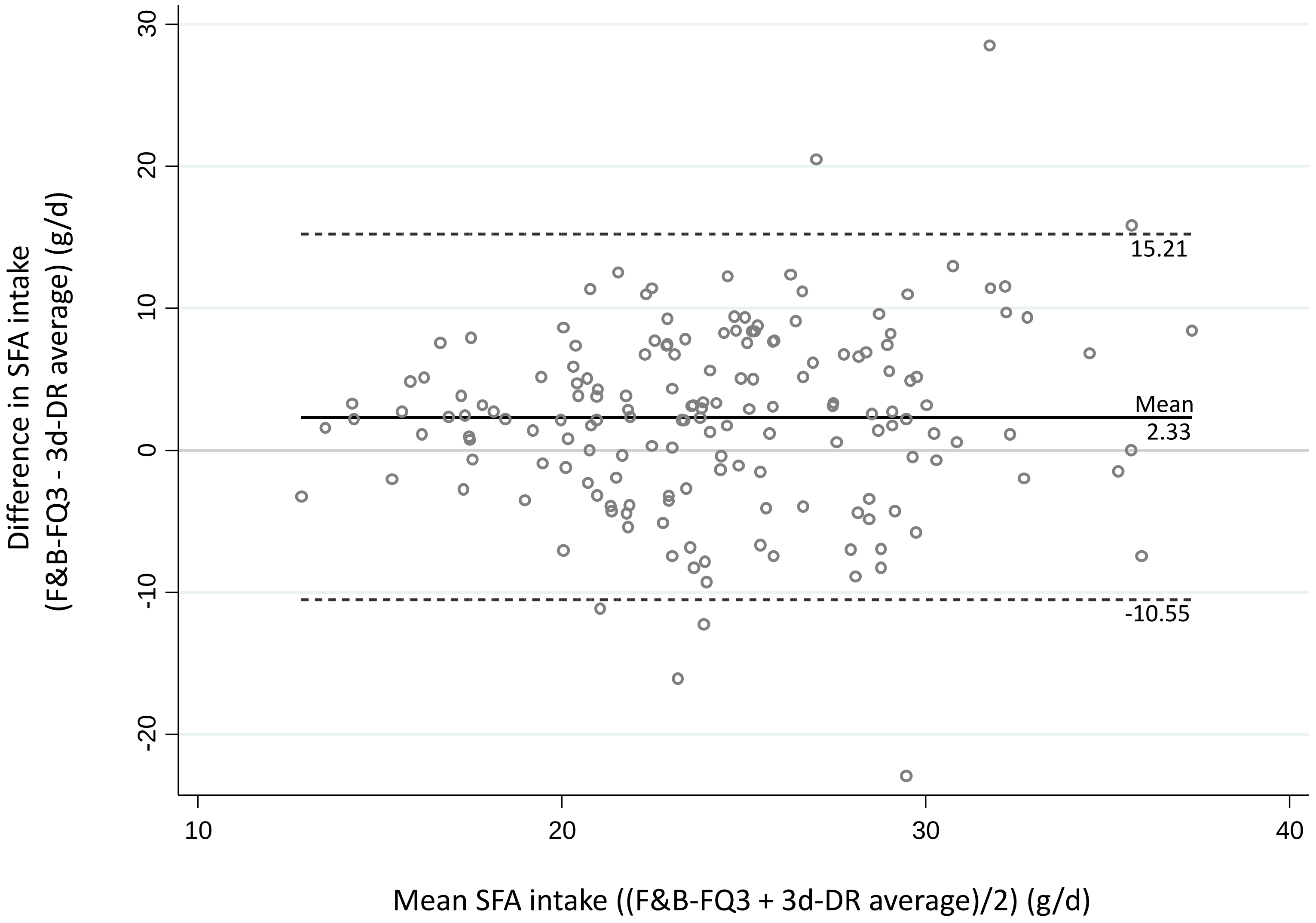
Difference in SFA intake
(F&B-FQ3 - 3d-DR average) (g/d)
30
20
10
0
-10
-20
15.21
Mean
2.33
-10.55
10
20
30
40
Mean SFA intake ((F&B-FQ3 + 3d-DR average)/2) (g/d)

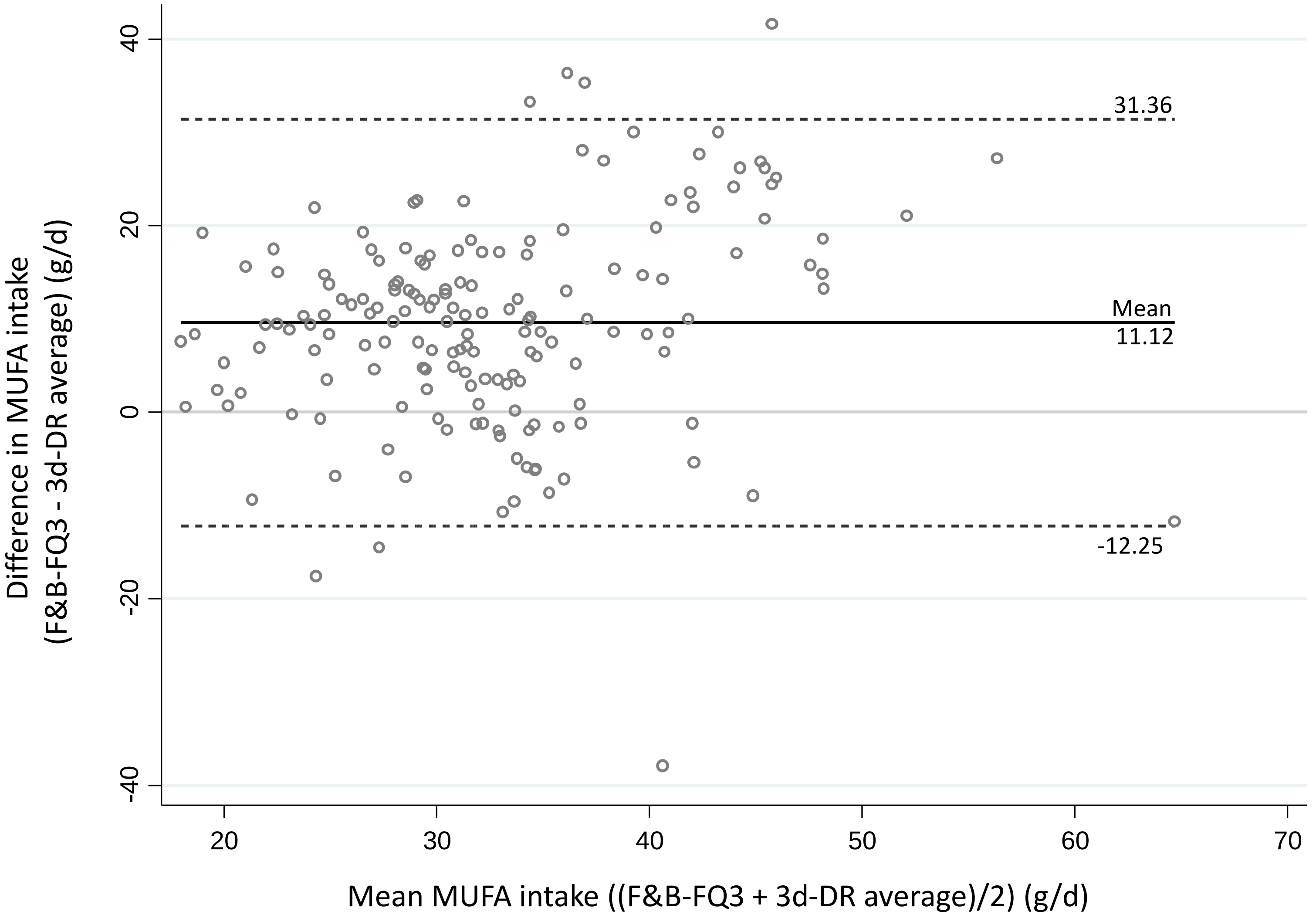

Difference in MUFA intake
(F&B-FQ3 - 3d-DR average) (g/d)
40
20
0
-20
-40
31.36
Mean
11.12
-12.25
20
30
40
50
60
70
Mean MUFA intake ((F&B-FQ3 + 3d-DR average)/2) (g/d)

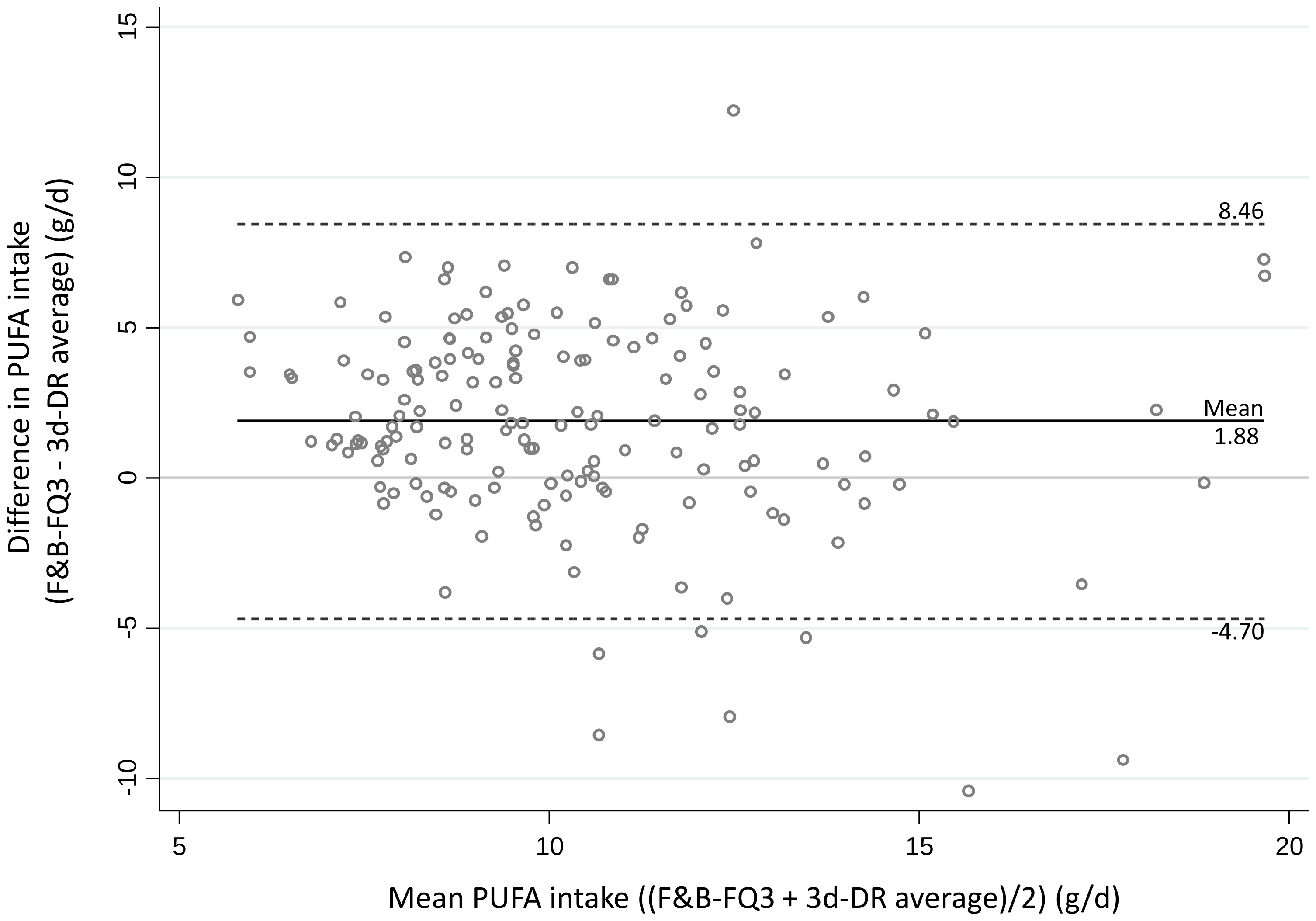

Difference in PUFA intake (F&B-FQ3 - 3d-DR average) (g/d)
15
10
5
0
-5
-10
8.46
Mean
1.88
-4.70
5
10
15
20
Mean PUFA intake ((F&B-FQ3 + 3d-DR average)/2) (g/d)

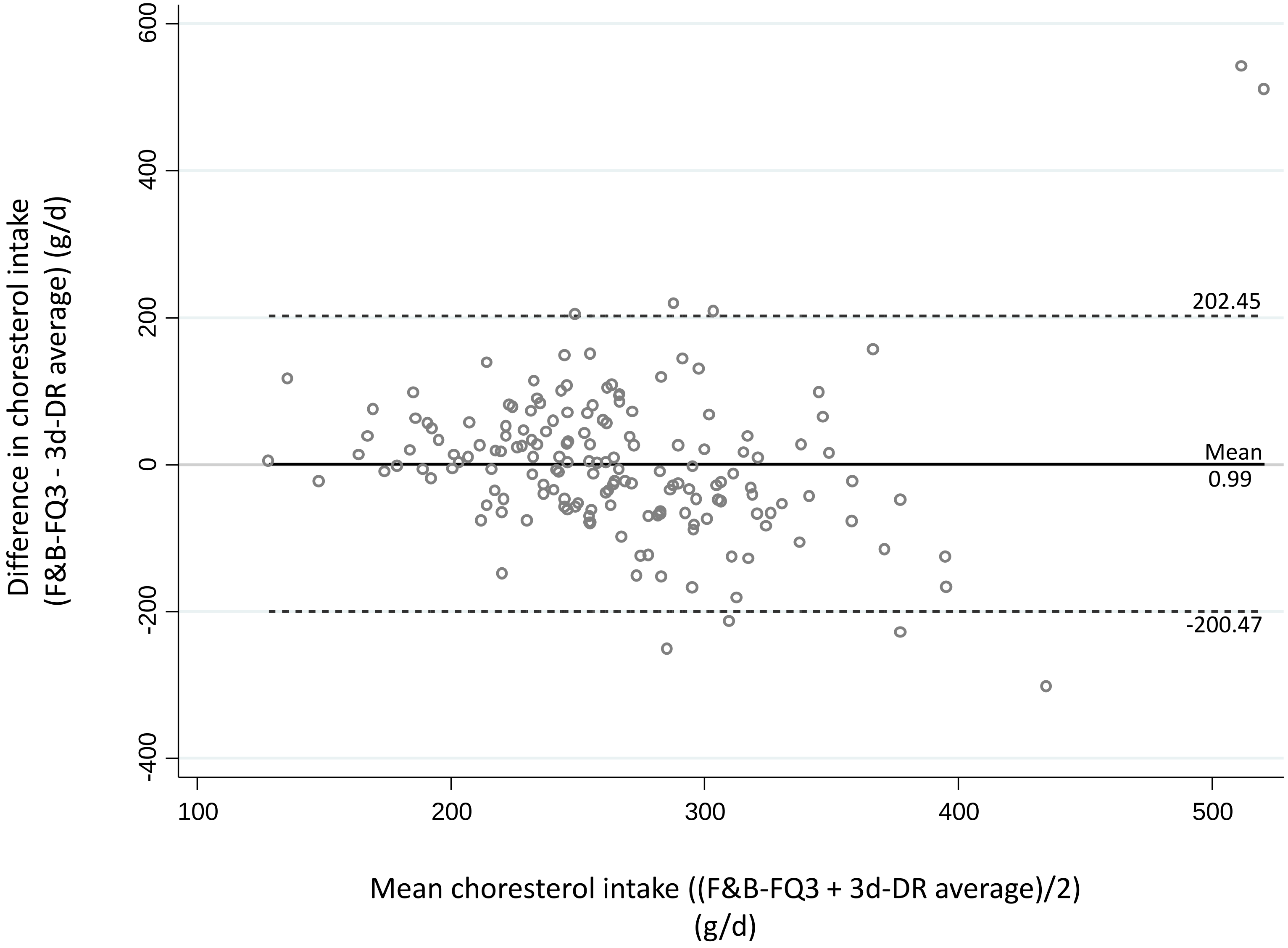

Difference in choresterol intake (F&B-FQ3 - 3d-DR average) (g/d)
600
400
200
0
-200
-400
202.45
Mean
0.99
-200.47
100
200
300
400
500
Mean choresterol intake ((F&B-FQ3 + 3d-DR average)/2) (g/d)

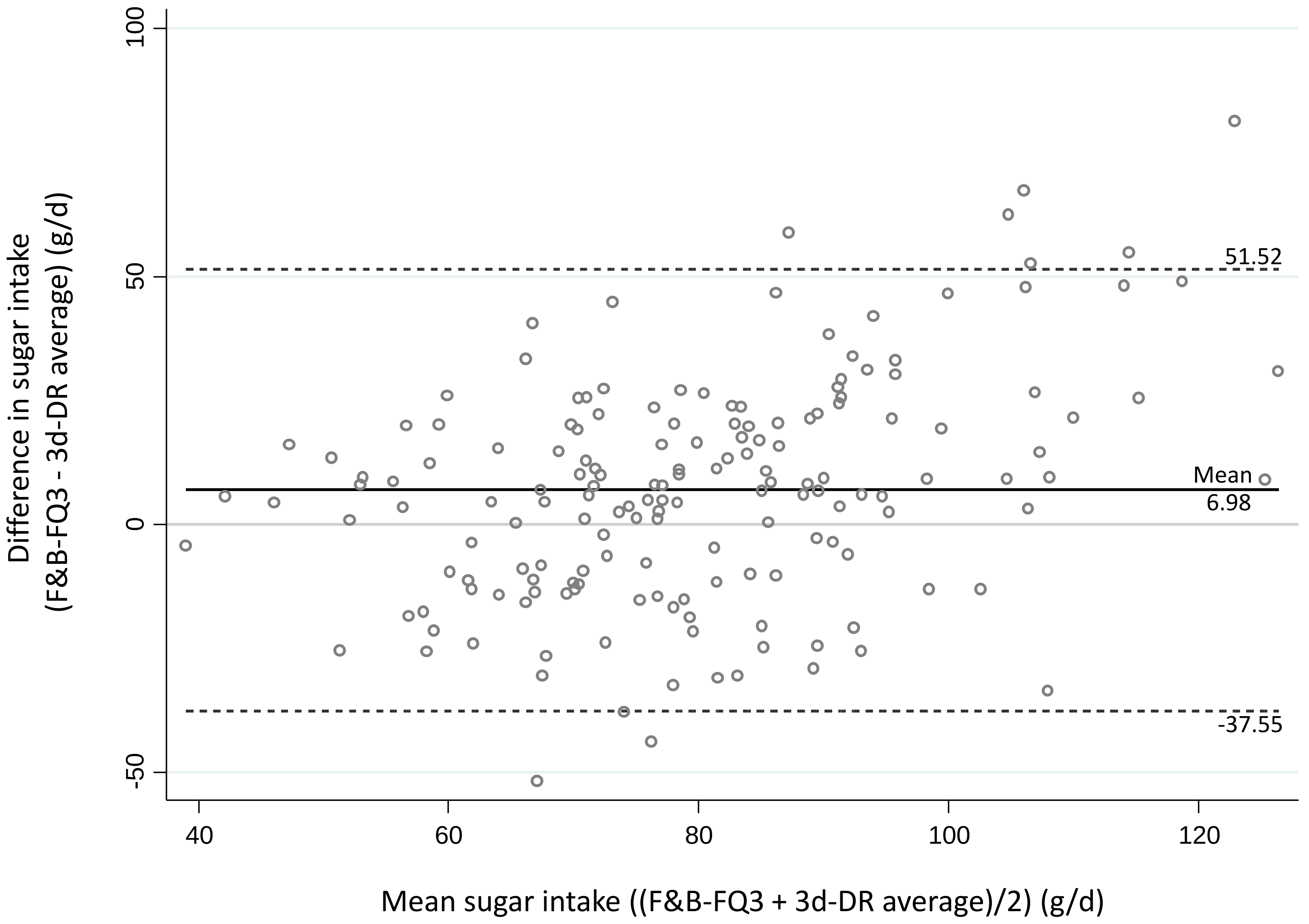

Difference in sugar intake
(F&B-FQ3 - 3d-DR average) (g/d)
100
50
0
-50
51.52
Mean
6.98
-37.55
40
60
80
100
120
Mean sugar intake ((F&B-FQ3 + 3d-DR average)/2) (g/d)

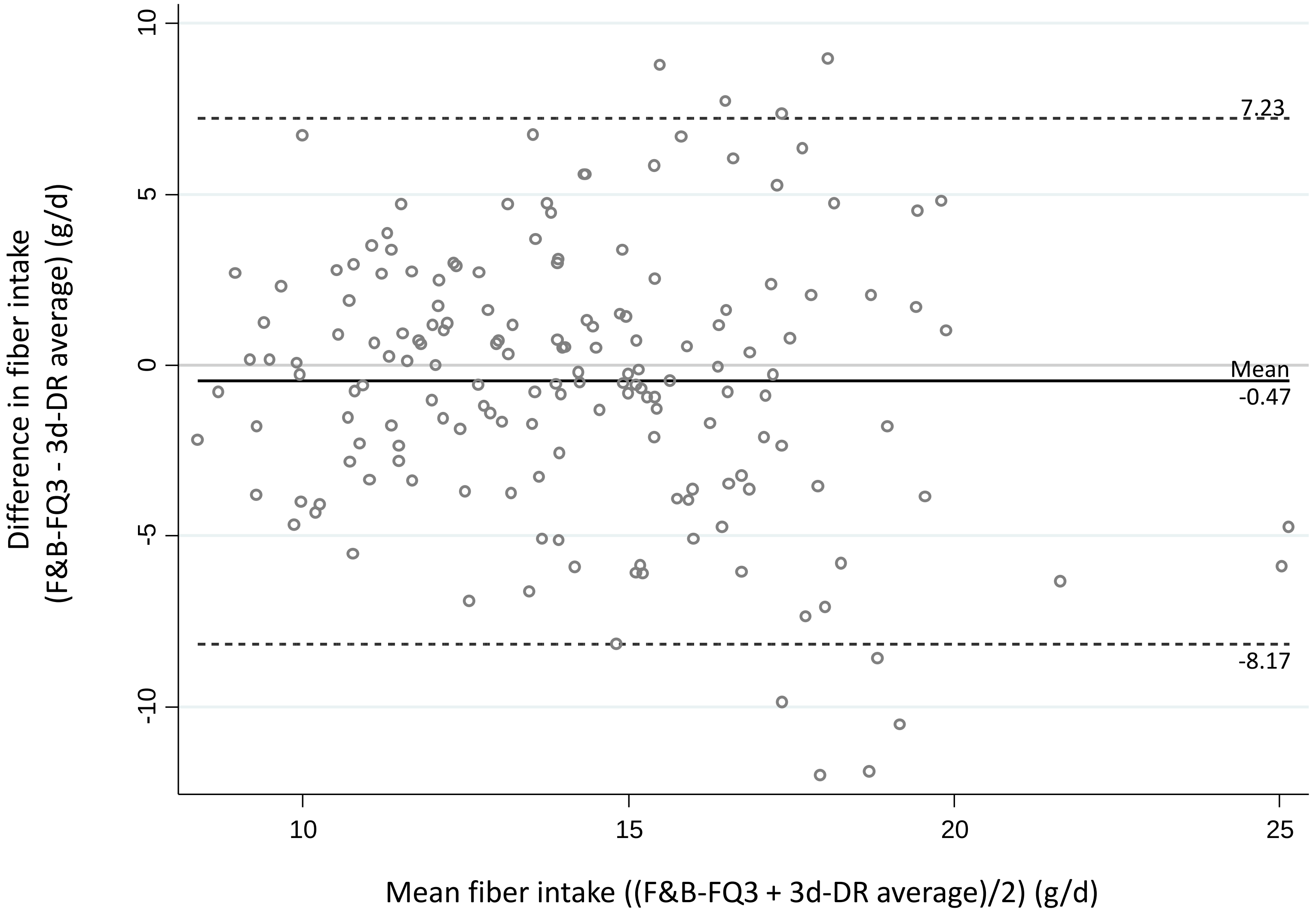

Difference in fiber intake
(F&B-FQ3 - 3d-DR average) (g/d)
10
5
0
-5
-10
7.23
Mean
-0.47
-8.17
10
15
20
25
Mean fiber intake ((F&B-FQ3 + 3d-DR average)/2) (g/d)

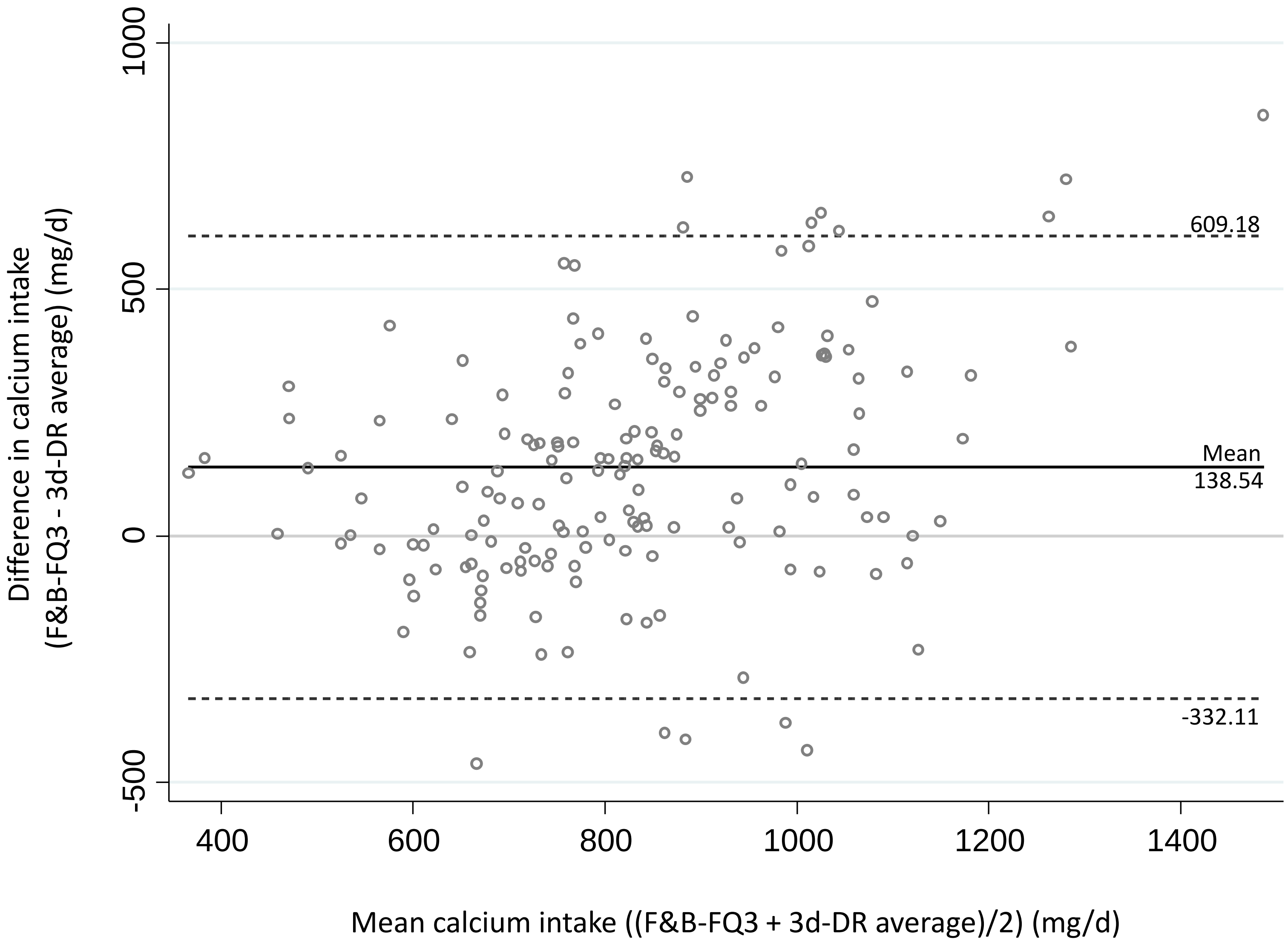

Difference in calcium intake
(F&B-FQ3 - 3d-DR average) (mg/d)
1000
500
0
-500
609.18
Mean
138.54
-332.11
400
600
800
1000
1200
1400
Mean calcium intake ((F&B-FQ3 + 3d-DR average)/2) (mg/d)

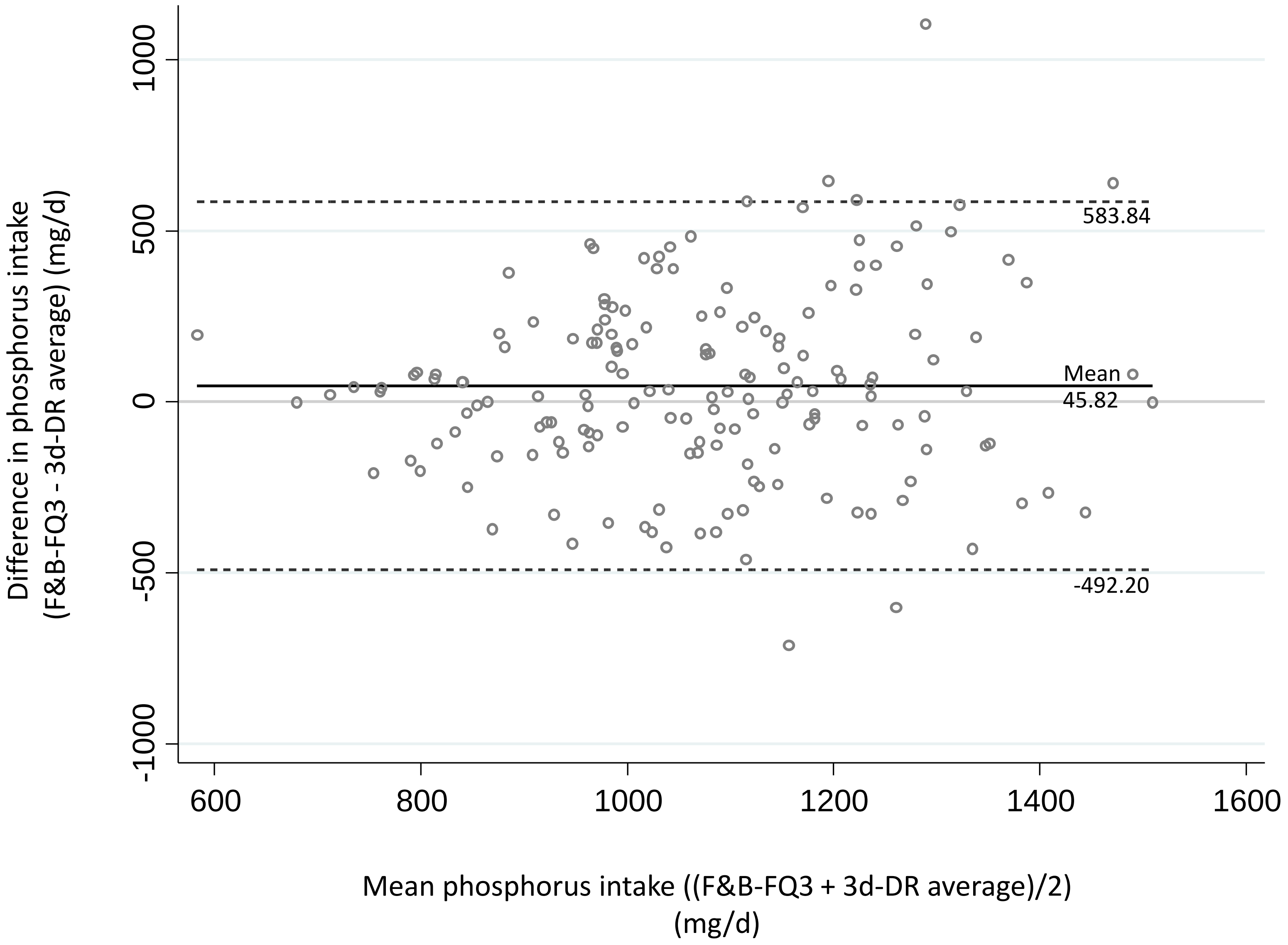
Difference in phosphorus intake
(F&B-FQ3 - 3d-DR average) (mg/d)
1000
500
0
-500
-1000
583.84
Mean
45.82
-492.20
600
800
1000
1200
1400
1600
Mean phosphorus intake ((F&B-FQ3 + 3d-DR average)/2)
(mg/d)

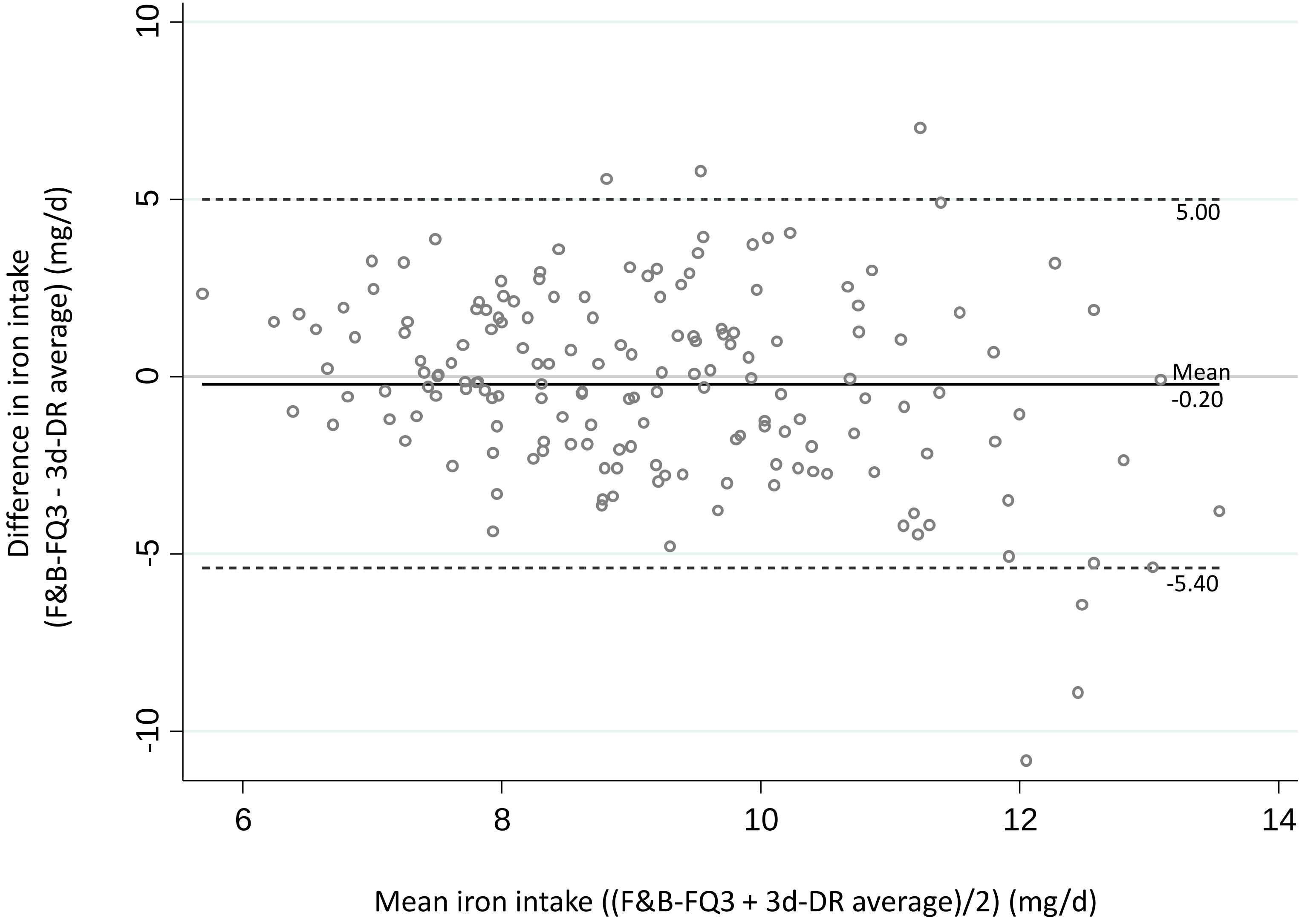

10
5
0
-5
-10
Difference in iron intake
(F&B-FQ3 - 3d-DR average) (mg/d)
5.00
Mean
-0.20
-5.40
6
8
10
12
14
Mean iron intake ((F&B-FQ3 + 3d-DR average)/2) (mg/d)

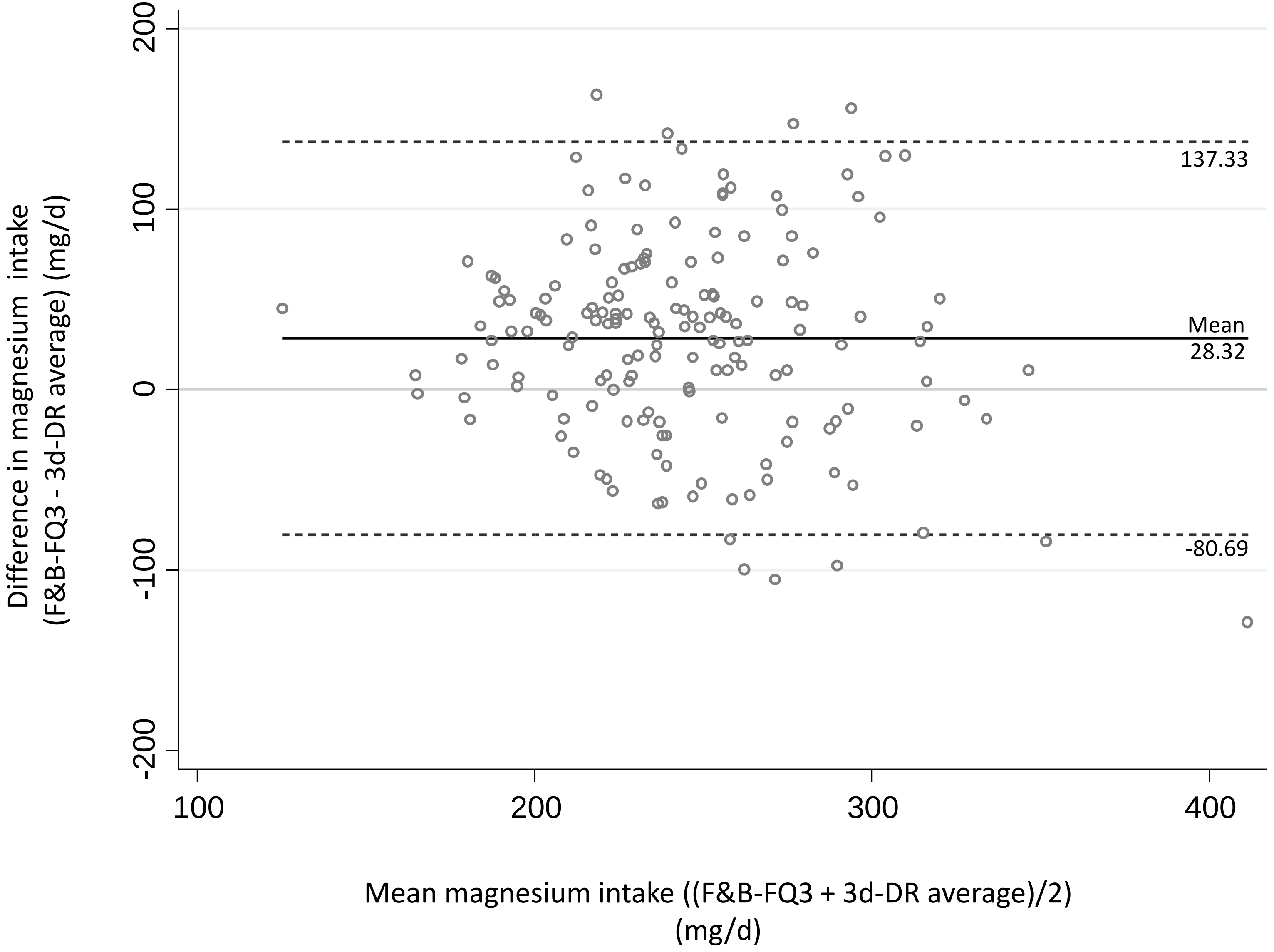

Difference in magnesium intake
(F&B-FQ3 - 3d-DR average) (mg/d)
200
100
0
-100
-200
137.33
Mean
28.32
-80.69
100
200
300
400
Mean magnesium intake ((F&B-FQ3 + 3d-DR average)/2)
(mg/d)

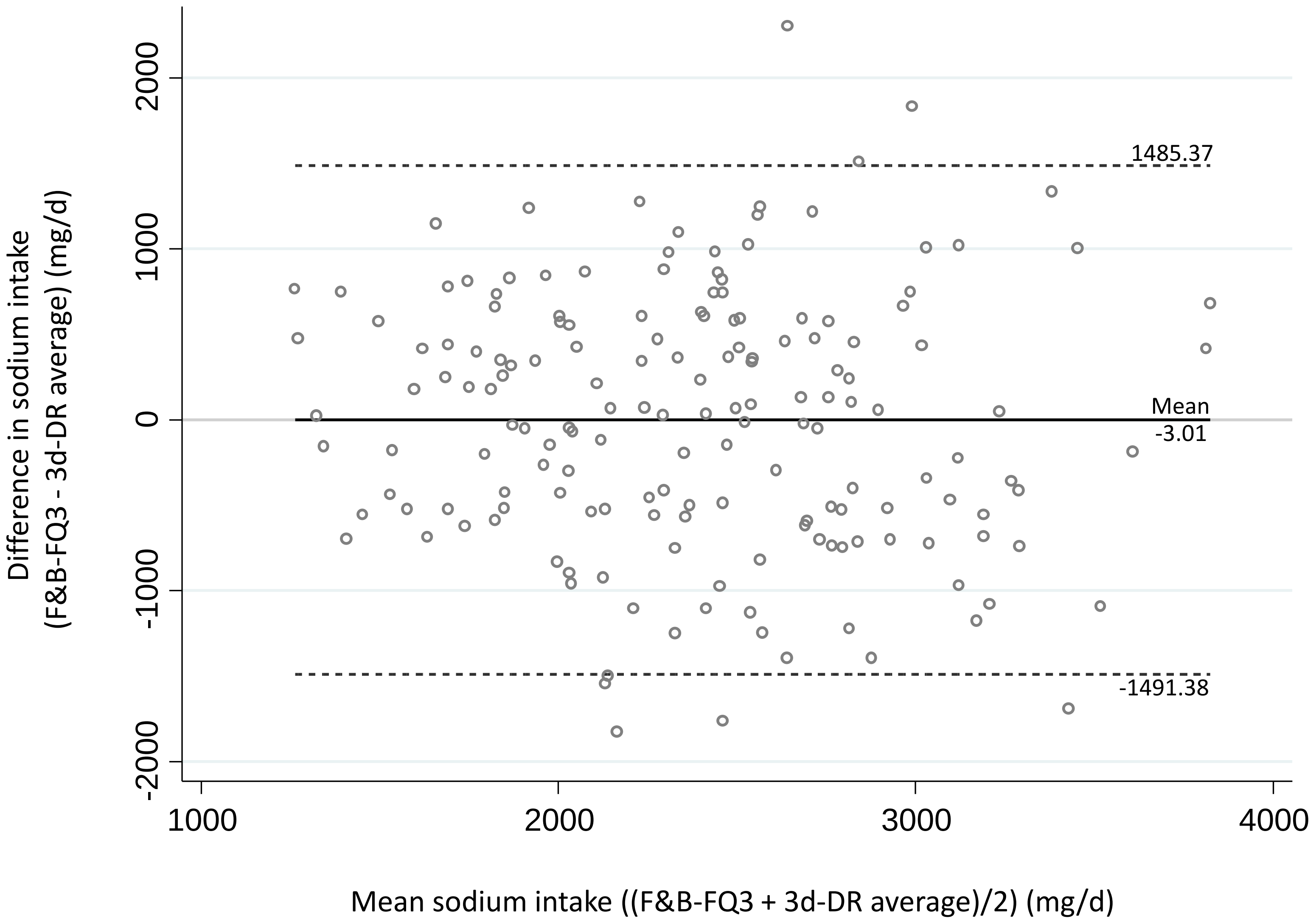

Difference in sodium intake
(F&B-FQ3 - 3d-DR average) (mg/d)
2000
1000
0
-1000
-2000
1485.37
Mean
-3.01
-1491.38
1000
2000
3000
4000
Mean sodium intake ((F&B-FQ3 + 3d-DR average)/2) (mg/d)

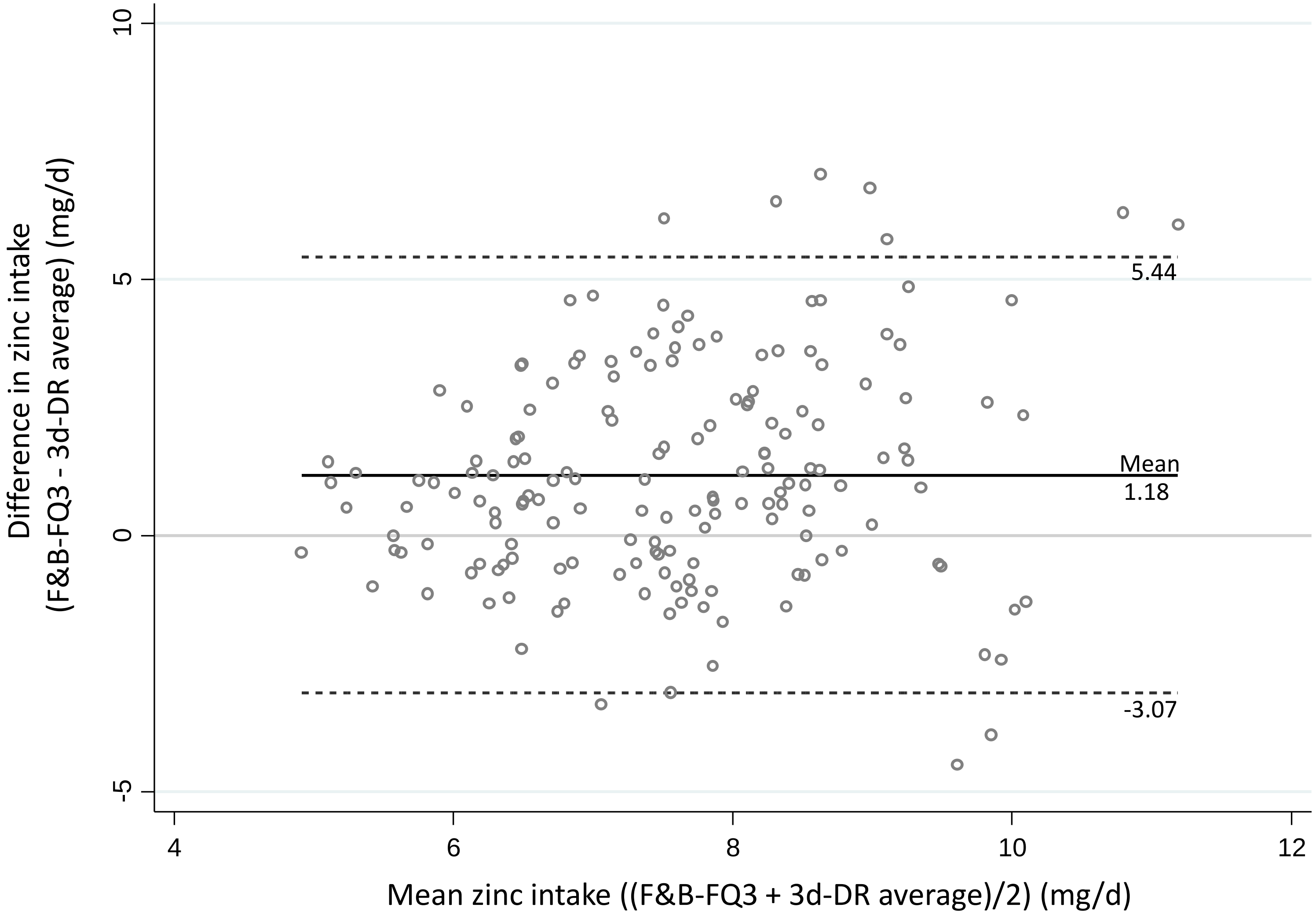

Difference in zinc intake
(F&B-FQ3 - 3d-DR average) (mg/d)
10
5
0
-5
5.44
Mean
1.18
-3.07
4
6
8
10
12
Mean zinc intake ((F&B-FQ3 + 3d-DR average)/2) (mg/d)

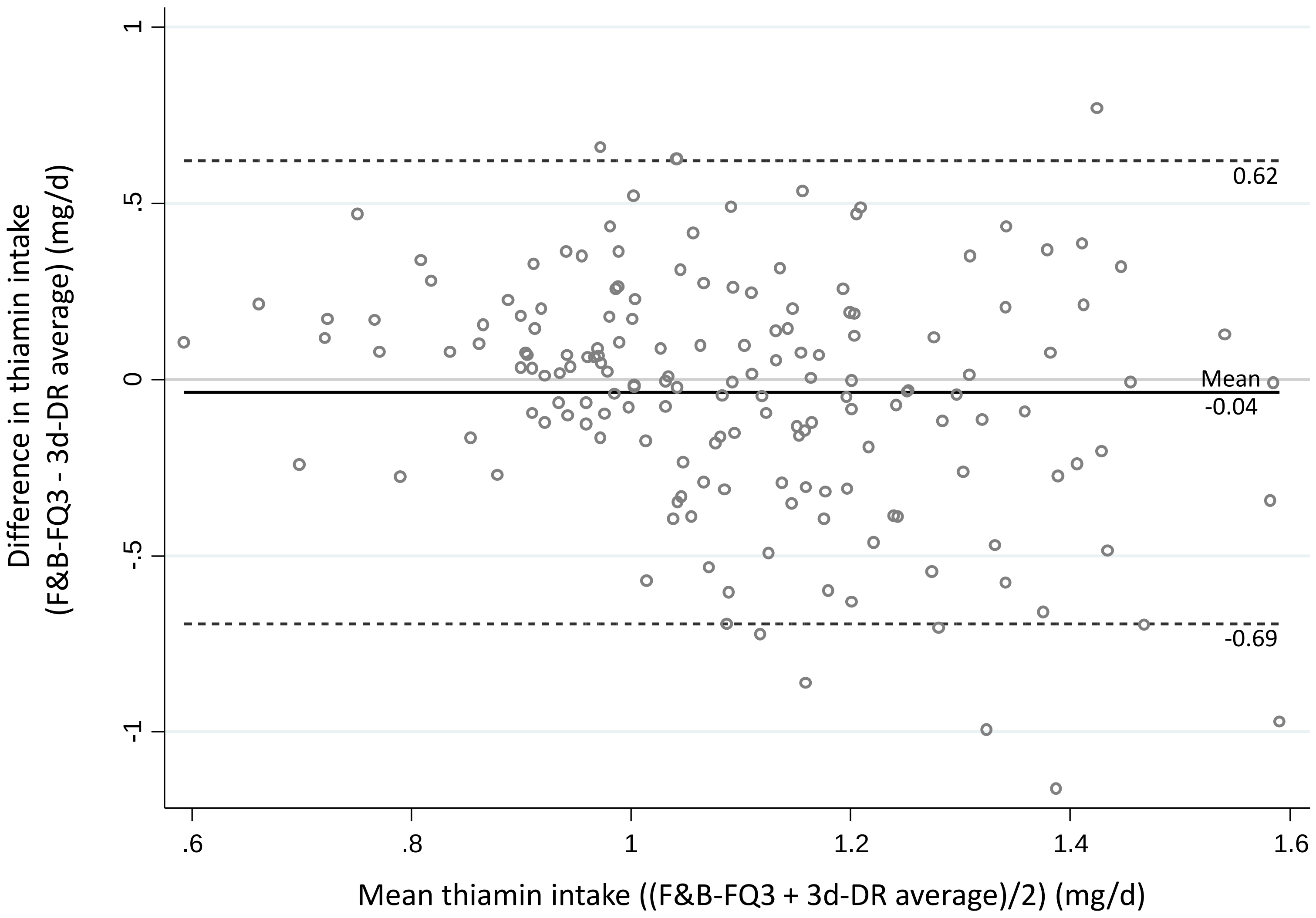
Difference in thiamin intake
(F&B-FQ3 - 3d-DR average) (mg/d)
Mean thiamin intake ((F&B-FQ3 + 3d-DR average)/2) (mg/d)
1
.5
0
-.5
-1
.6
.8
1
1.2
1.4
1.6
0.62
Mean
-0.04
-0.69

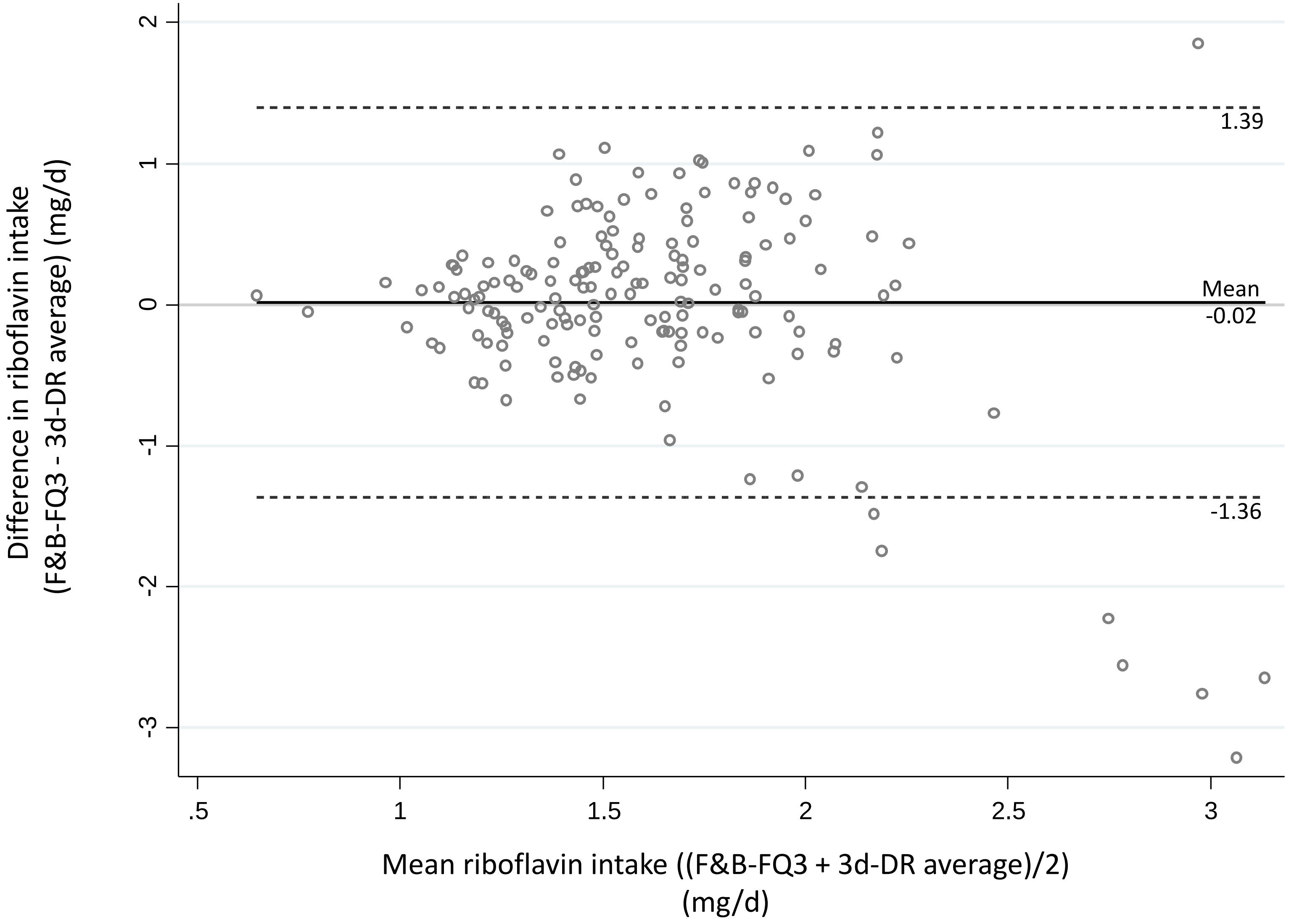

Difference in riboflavin intake
(F&B-FQ3 - 3d-DR average) (mg/d)
2
1
0
-1
-2
-3
1.39
Mean
-0.02
-1.36
.5
1
1.5
2
2.5
3
Mean riboflavin intake ((F&B-FQ3 + 3d-DR average)/2)
(mg/d)

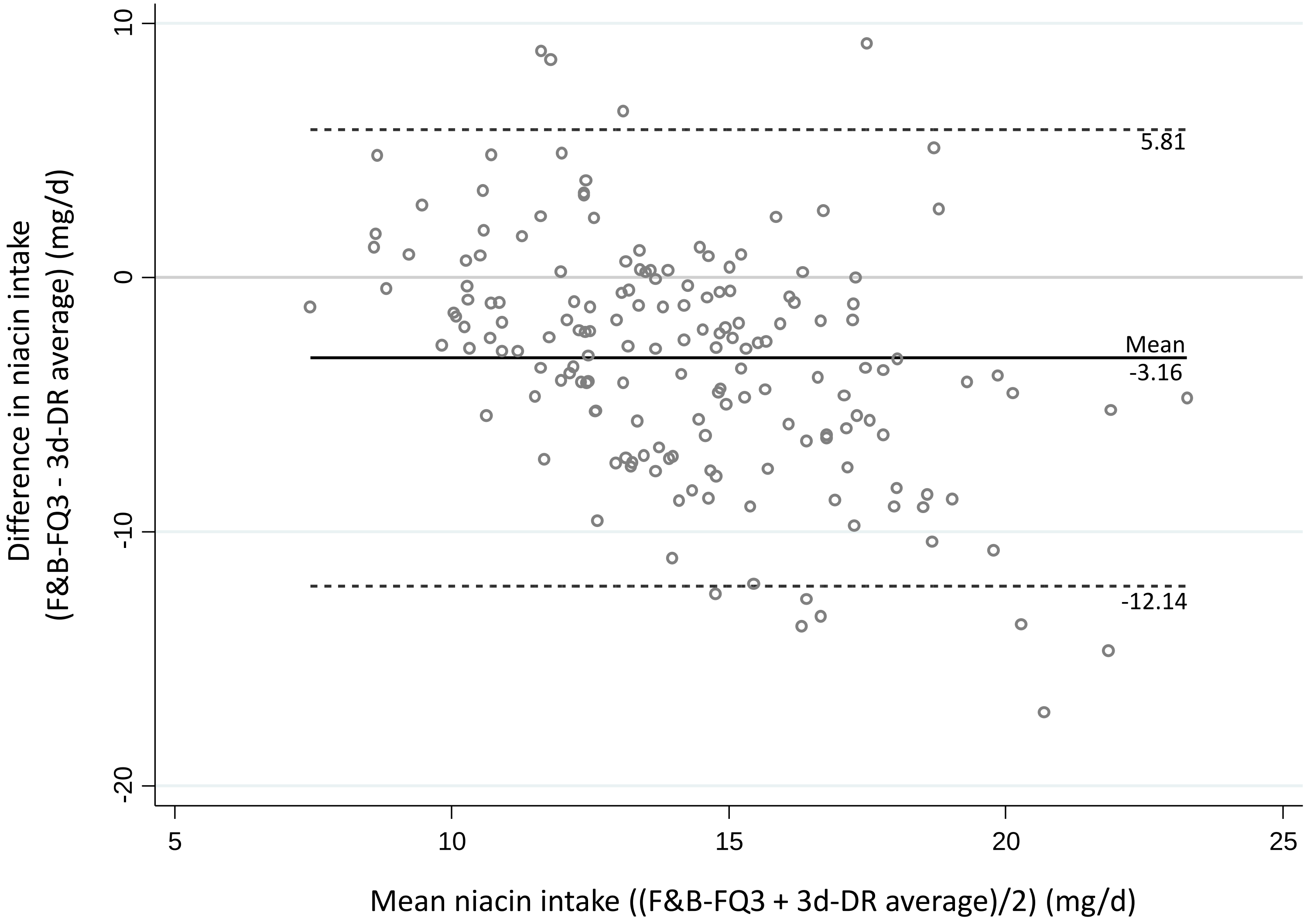

Difference in niacin intake
(F&B-FQ3 - 3d-DR average) (mg/d)
10
0
-10
-20
5.81
Mean
-3.16
-12.14
5
10
15
20
25
Mean niacin intake ((F&B-FQ3 + 3d-DR average)/2) (mg/d)

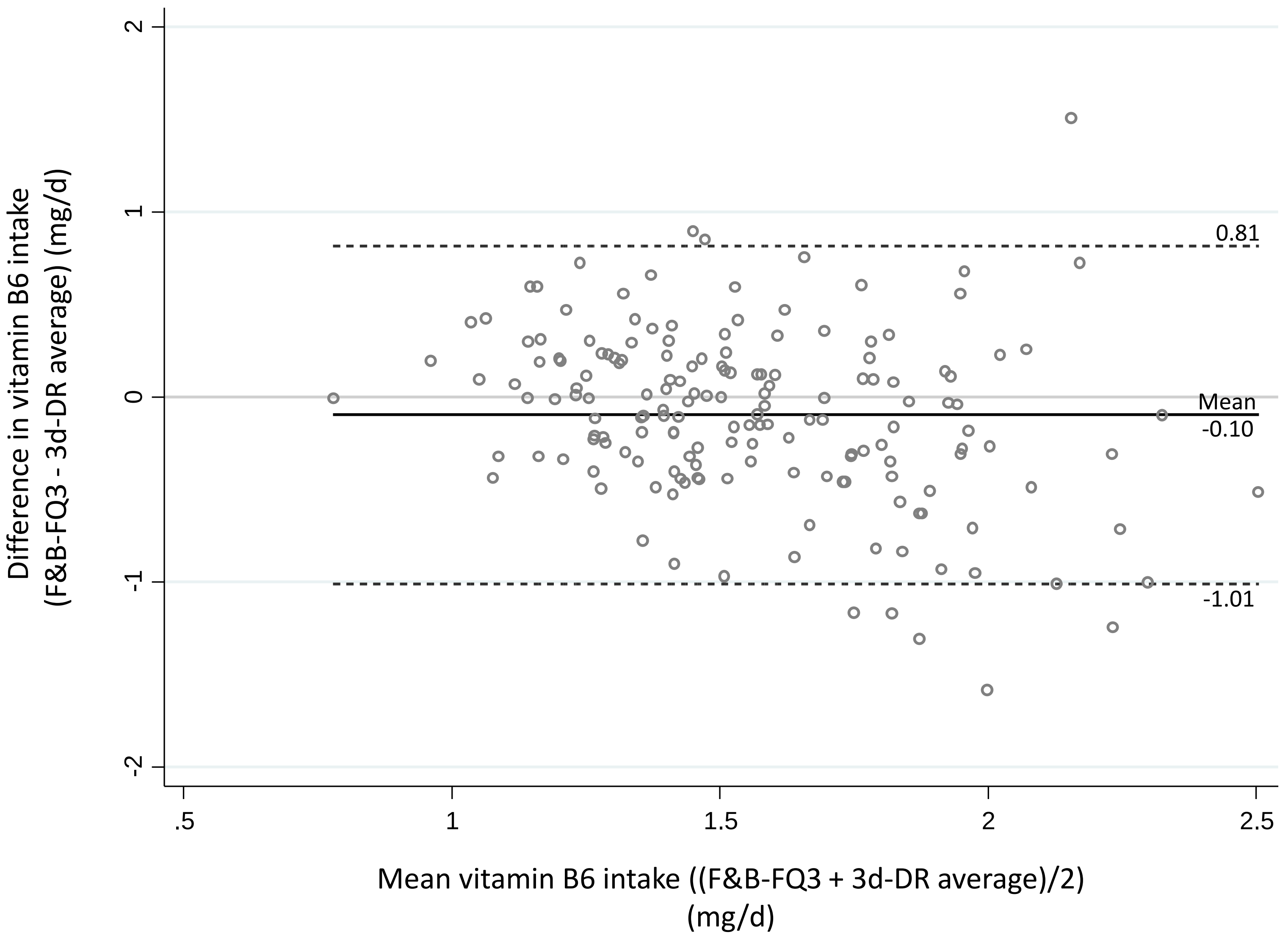

Difference in vitamin B6 intake
(F&B-FQ3 - 3d-DR average) (mg/d)
2
1
0
-1
-2
0.81
Mean
-0.10
-1.01
.5
1
1.5
2
2.5
Mean vitamin B6 intake ((F&B-FQ3 + 3d-DR average)/2)
(mg/d)

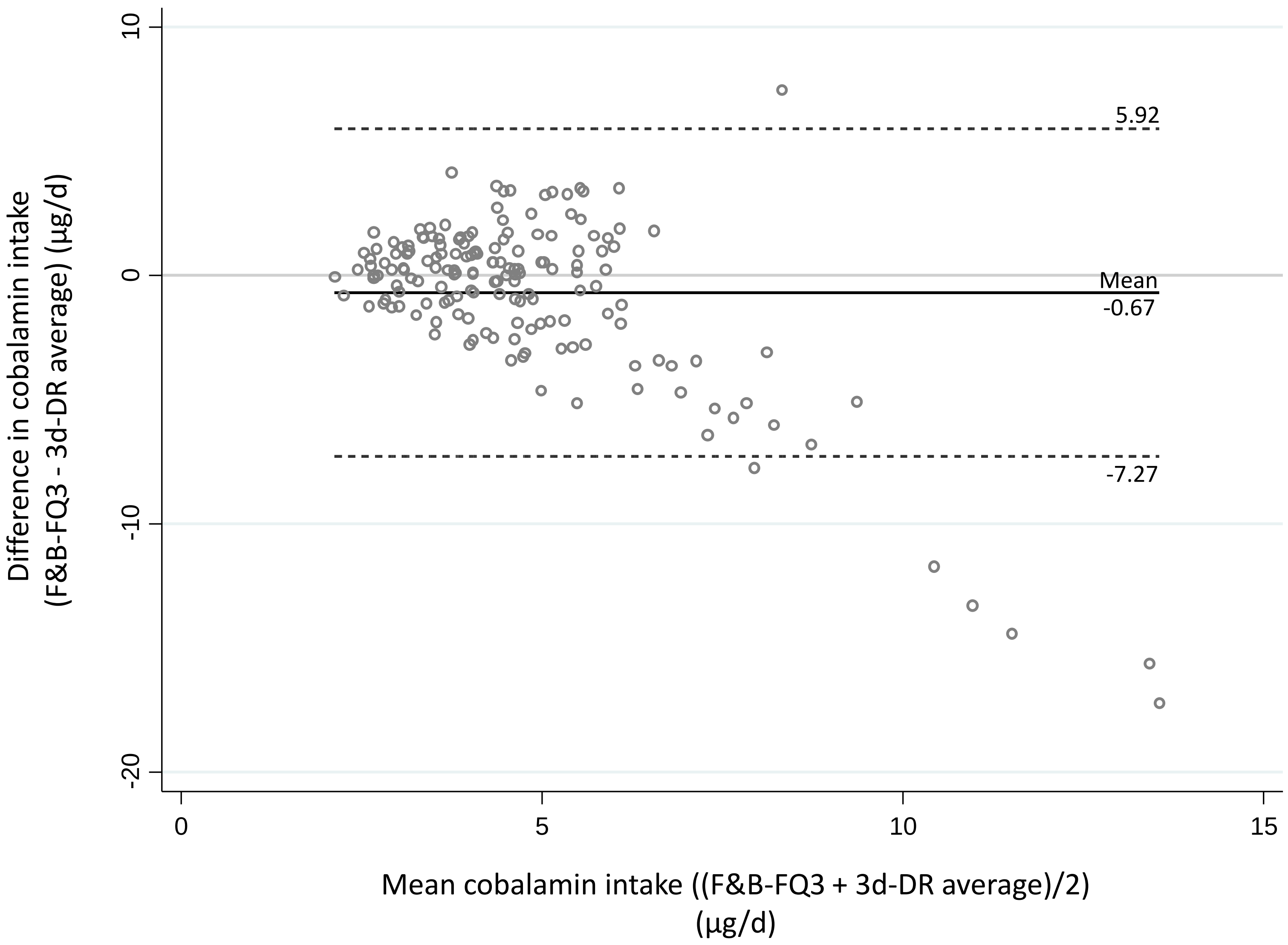
Difference in cobalamin intake
(F&B-FQ3 - 3d-DR average) (µg/d)
10
0
-10
-20
5.92
Mean
-0.67
-7.27
0
5
10
15
Mean cobalamin intake ((F&B-FQ3 + 3d-DR average)/2)
(µg/d)

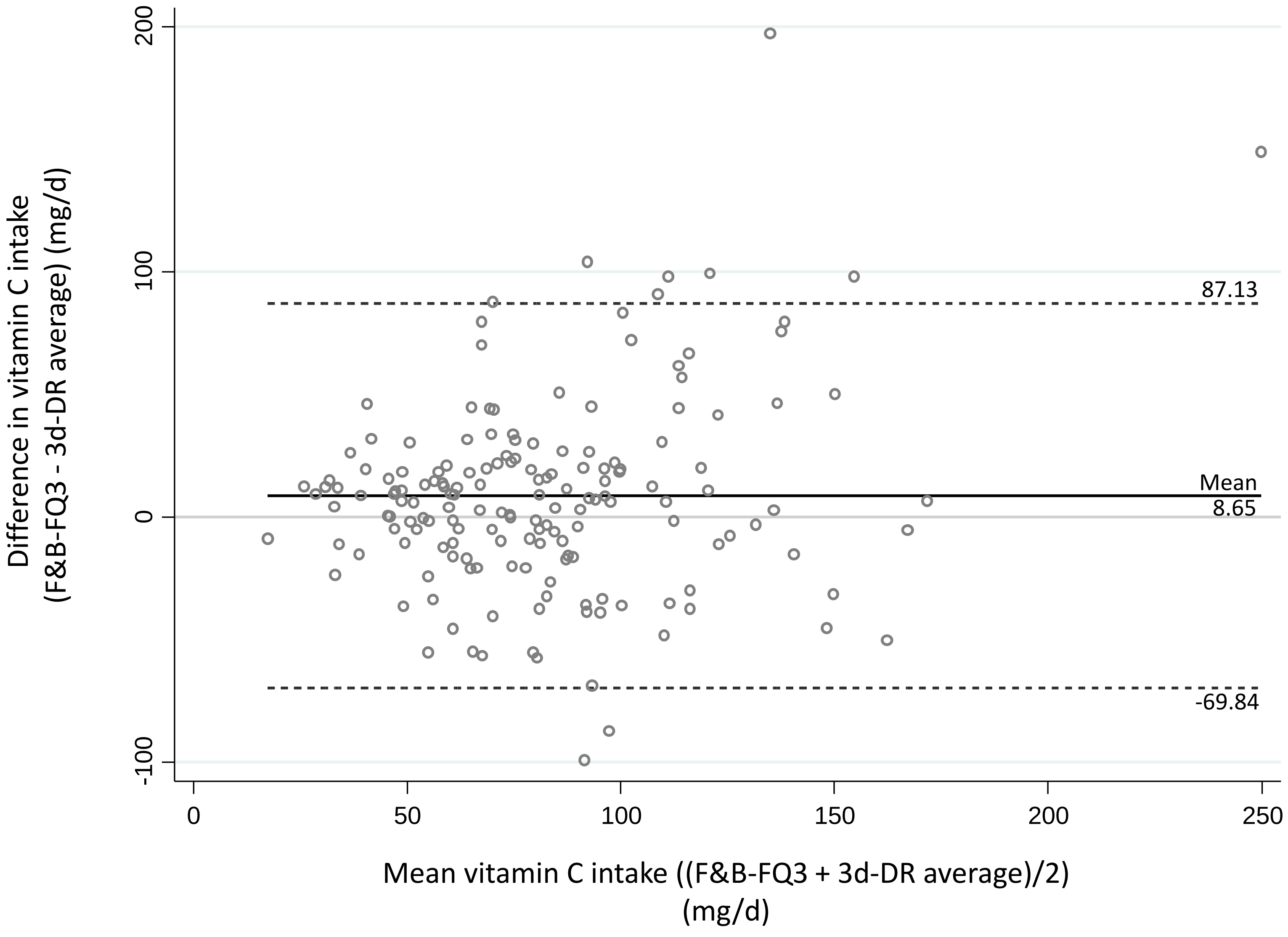

200
100
0
-100
0
50
100
150
200
250
87.13
Mean
8.65
-69.84
Difference in vitamin C intake
(F&B-FQ3 - 3d-DR average) (mg/d)
Mean vitamin C intake ((F&B-FQ3 + 3d-DR average)/2)
(mg/d)

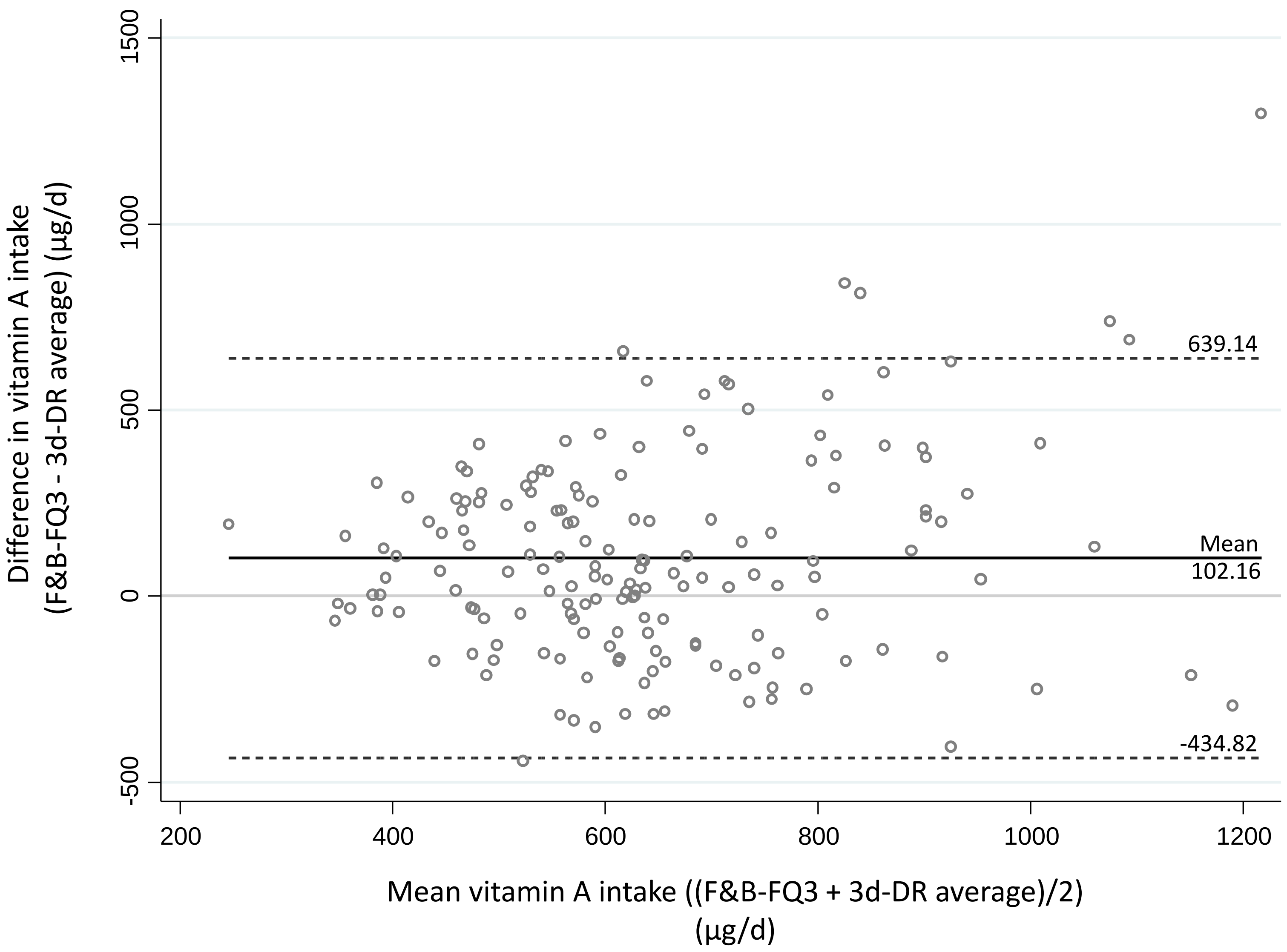

Difference in vitamin A intake
(F&B-FQ3 - 3d-DR average) (µg/d)
1500
1000
500
0
-500
639.14
Mean
102.16
-434.82
200
400
600
800
1000
1200
Mean vitamin A intake ((F&B-FQ3 + 3d-DR average)/2)
(µg/d)

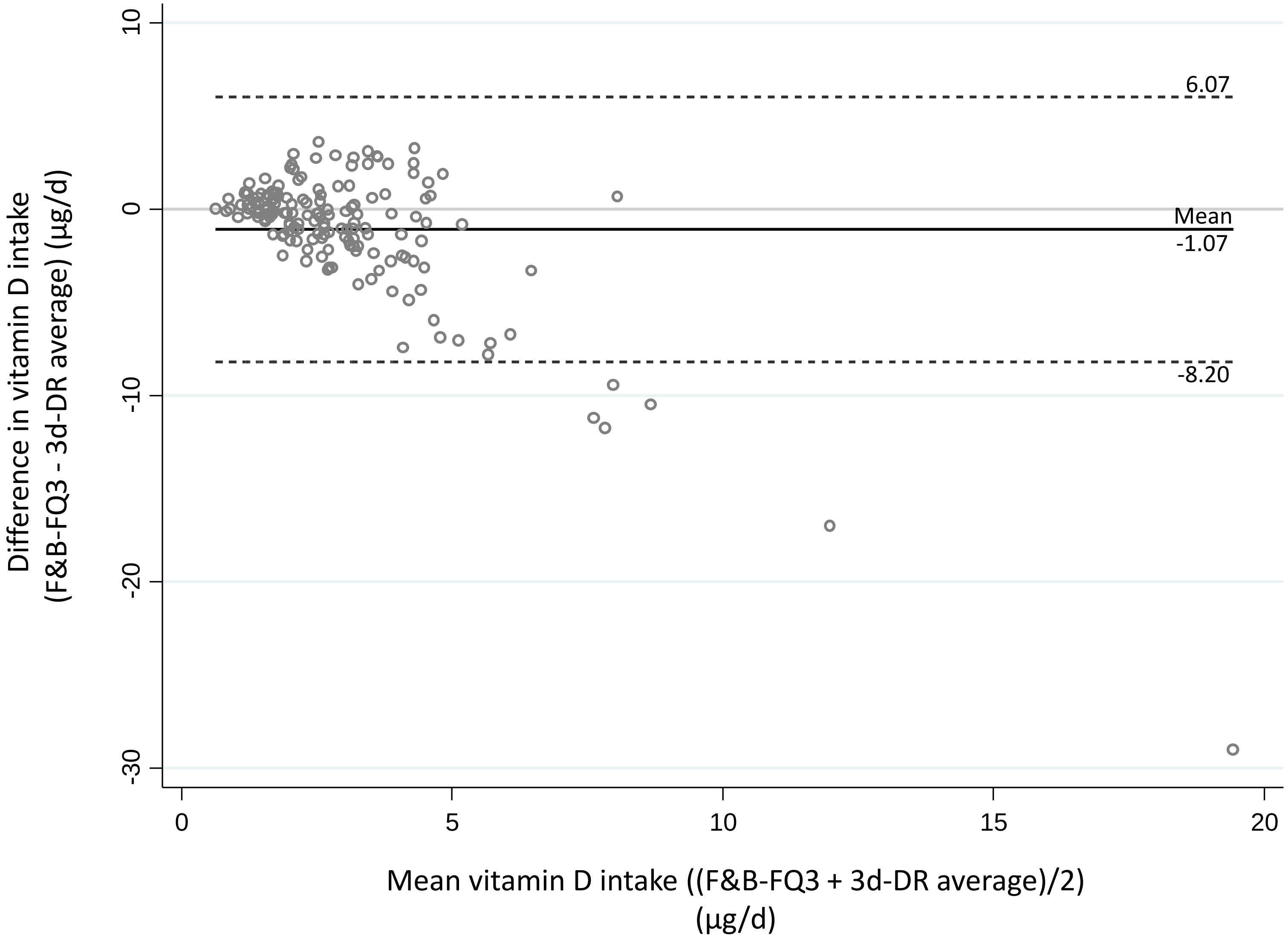

Difference in vitamin D intake
(F&B-FQ3 - 3d-DR average) (µg/d)
10
0
-10
-20
-30
6.07
Mean
-1.07
-8.20
0
5
10
15
20
Mean vitamin D intake ((F&B-FQ3 + 3d-DR average)/2)
(µg/d)

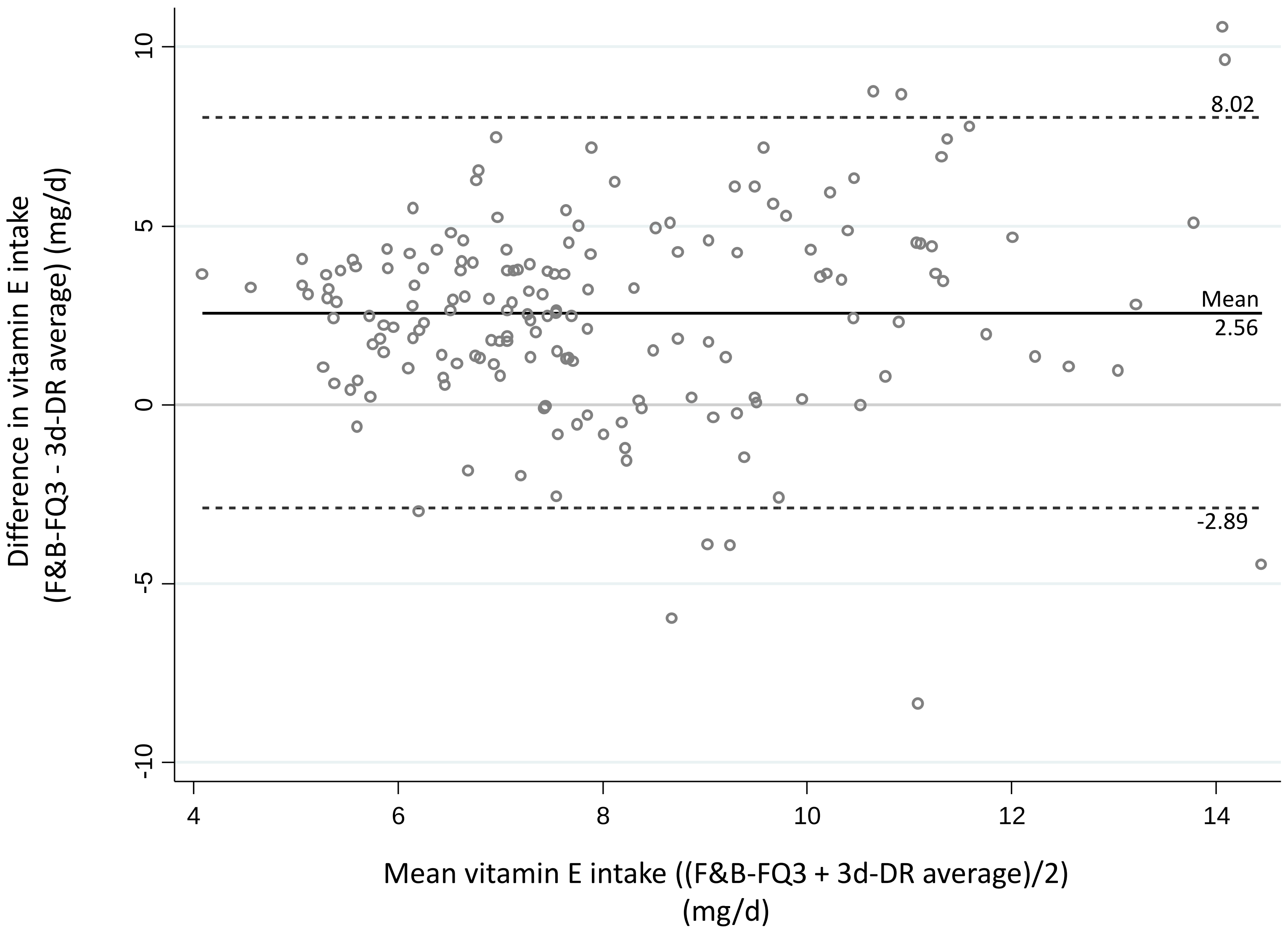
10
5
0
-5
-10
Difference in vitamin E intake
(F&B-FQ3 - 3d-DR average) (mg/d)
8.02
Mean
2.56
-2.89
4
6
8
10
12
14
Mean vitamin E intake ((F&B-FQ3 + 3d-DR average)/2)
(mg/d)

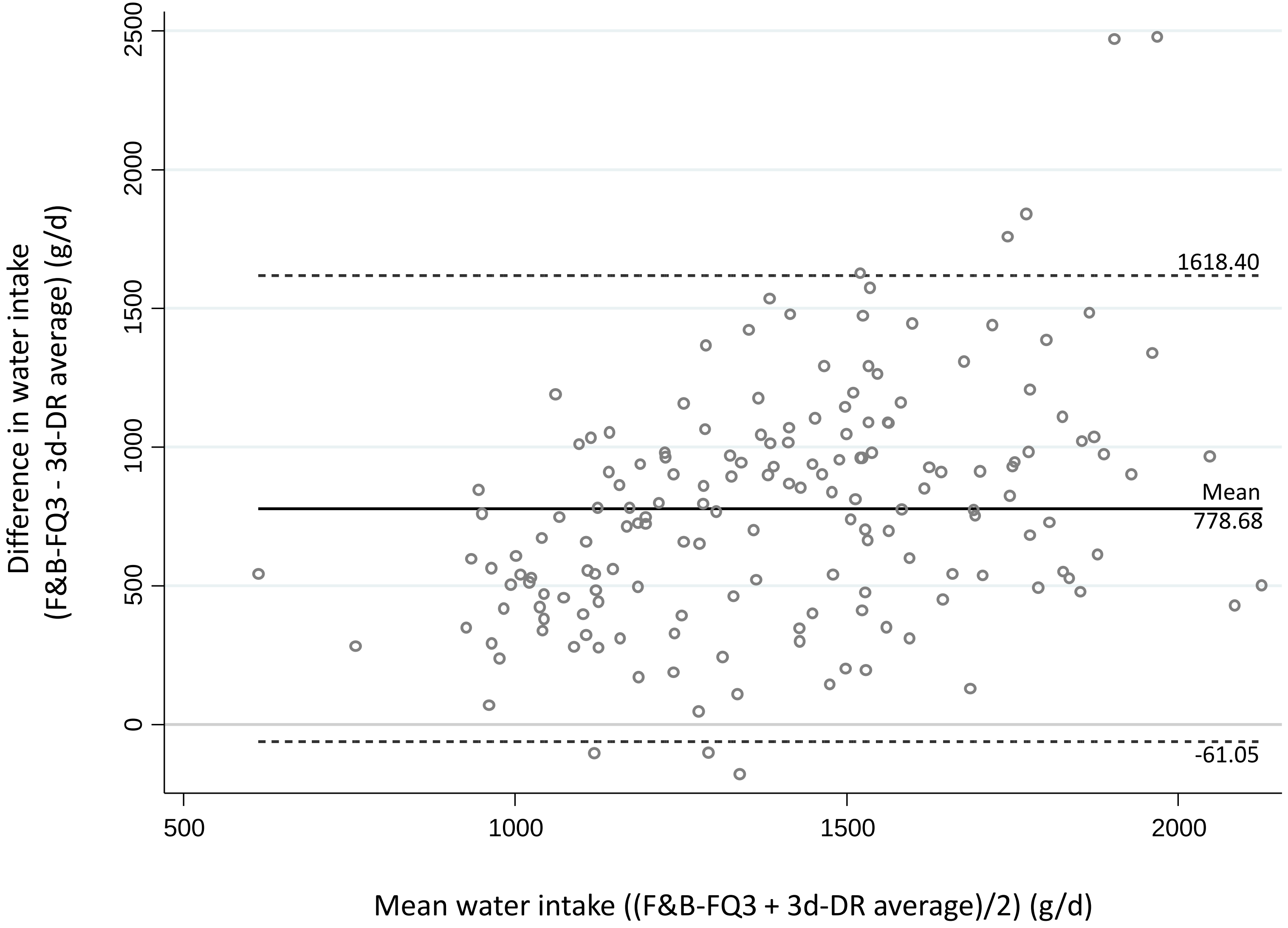

2500
2000
1500
1000
500
0
Difference in water intake
(F&B-FQ3 - 3d-DR average) (g/d)
1618.40
Mean
778.68
-61.05
500
1000
1500
2000
Mean water intake ((F&B-FQ3 + 3d-DR average)/2) (g/d)
